# Isotopic evidence of biotrophy and unusual nitrogen nutrition in soil‐dwelling Hygrophoraceae

**DOI:** 10.1111/1462-2920.14327

**Published:** 2018-10-17

**Authors:** Hans Halbwachs, Gary L. Easton, Roland Bol, Erik A. Hobbie, Mark H Garnett, Derek Peršoh, Liz Dixon, Nick Ostle, Peter Karasch, Gareth W. Griffith

**Affiliations:** ^1^ Bavarian Forest National Park Freyunger Str. 2, 94481, Grafenau Germany; ^2^ Institute of Biological, Environmental and Rural Sciences, Aberystwyth University Adeilad Cledwyn, Penglais, Aberystwyth, Ceredigion, SY23 3DD, Wales UK; ^3^ Institute of Bio‐ and Geosciences, Agrosphere (IBG‐3). Forschungszentrum Jülich GmbH Wilhelm‐Johnen‐Straße, 52428, Jülich Germany; ^4^ Earth Systems Research Center, Morse Hall University of New Hampshire 8 College Road, Durham NH, 03824‐3525 USA; ^5^ NERC Radiocarbon Facility Scottish Enterprise Technology Park Rankine Avenue, East Kilbride, G75 0QF Scotland, UK; ^6^ Department of Geobotany Ruhr‐Universität Bochum Gebäude ND 03/170, Universitätsstraße 150, 44780, Bochum Germany; ^7^ Sustainable Soils and Grassland Systems, Rothamsted Research North Wyke, Okehampton, Devon, EX20 2SB England, UK; ^8^ Lancaster Environment Centre Lancaster University Lancaster, LA1 4YQ England, UK; ^9^ German Mycological Society Kirchl 78. D‐94545, Hohenau Germany

## Abstract

Several lines of evidence suggest that the agaricoid, non‐ectomycorrhizal members of the family Hygrophoraceae (waxcaps) are biotrophic with unusual nitrogen nutrition. However, methods for the axenic culture and lab‐based study of these organisms remain to be developed, so our current knowledge is limited to field‐based investigations. Addition of nitrogen, lime or organophosphate pesticide at an experimental field site (Sourhope) suppressed fruiting of waxcap basidiocarps. Furthermore, stable isotope natural abundance in basidiocarps were unusually high in ^15^N and low in ^13^C, the latter consistent with mycorrhizal nutritional status. Similar patterns were found in waxcap basidiocarps from diverse habitats across four continents. Additional data from ^14^C analysis of basidiocarps and ^13^C pulse label experiments suggest that these fungi are not saprotrophs but rather biotrophic endophytes and possibly mycorrhizal. The consistently high but variable δ^15^N values (10–20‰) of basidiocarps further indicate that N acquisition or processing differ from other fungi; we suggest that N may be derived from acquisition of N via soil fauna high in the food chain.

## Originality‐significance statement

This manuscript presents the results of experiments to elucidate the nutritional biology of soil‐dwelling Hygrophoraceae (waxcaps). This major group of agaricoid fungi is primarily known from undisturbed grassland habitats where they are dominant components of the soil fungal community, more abundant than arbuscular mycorrhizal fungi (Detheridge *et al*., 2018). However, their resistance to axenic culture has hindered study of their biology, so they are often (by default) considered as saprotrophs. Here we combine field surveys assessing the effects of agricultural manipulations on fruiting with isotopic (^13^C, ^15^N and ^14^C) analyses to show that they are biotrophs, potentially forming mycorrhizal associations with host plants. Whilst the ^13^C and ^15^N patterns of their basidiocarps resemble ectomycorrhizal fungi more than saprotrophs, their consistently high δ^15^N values also indicate an unusual mode of nitrogen nutrition, for which we suggest some possible mechanisms.

## Introduction

Members of the agaric family Hygrophoraceae exhibit diverse nutritional strategies, ranging from lichenised forms associated with both green algae (*Lichenomphalia* spp.) and cyanobacteria (*Dictyonema* spp.) to ectomycorrhizal (ECM) taxa (*Hygrophorus* spp.) (Agerer, 2006; Seitzman *et al*., 2011; Lodge *et al*., 2014). However, the nutritional strategies of several genera within this family are less certain, notably the soil‐dwelling taxa *Hygrocybe*, *Cuphophyllus*, *Gliophorus*, *Humidicutis*, *Chromosera*, *Neohygrocybe* and *Porpolomopsis* (Griffith *et al*., 2002; Halbwachs *et al*., 2013a; Lodge *et al*., 2014). Though associated with undisturbed grasslands in Europe (Griffith *et al*., 2004; Halbwachs *et al*., 2013a), at a global level they are more commonly associated with forest habitats but not with tree species that form ectomycorrhizas (Seitzman *et al*., 2011; Lodge *et al*., 2014). Here we use the term ‘waxcap’ to refer to soil‐dwelling Hygrophoraceae not known to be ectomycorrhizal or lichenised (Boertmann, 2010).

In the European context, interest in the Hygrophoraceae stems mainly from their high conservation value, as they only fruit (often abundantly) in undisturbed grasslands. Waxcap basidiocarps are much rarer or absent in grasslands subject to agricultural intensification so these fungi have suffered large‐scale habitat loss, especially in lowland areas. Recent investigation of grassland fungi using DNA metabarcoding has shown that Hygrophoraceae are amongst the most abundant fungi in undisturbed grassland soils (Detheridge *et al*., 2018). However, the effects of fertilizer or lime additions, whilst widely reported to inhibit waxcap fruiting, have not been rigorously quantified. Thus, a better understanding of their nutritional requirements and ecological interactions would be beneficial to halt further losses.

Waxcaps are generally referred to as ‘saprotrophs’ in the mycological literature (Keizer, 1993; Tedersoo *et al*., 2010) and they are currently listed as such in FUNguild (Nguyen *et al*., 2015) (http://github.com/UMNFuN/FUNGuild), most likely by default (i.e. lack of published evidence to the contrary). However, several lines of evidence now suggest that they are biotrophic, similar to their lichenised and ectomycorrhizal relatives in family Hygrophoraceae, amongst which they are interspersed (Seitzman *et al*., 2011; Lodge *et al*., 2014). They have fastidious nutritional requirements, as evidenced by their recalcitrance to axenic culture and the failure of their spores to germinate on various agar media (Beisenherz, 2000; Griffith and Roderick, 2008; Roderick, 2009). They can colonize the root hairs of grassland plants and their DNA has been detected within plant tissues (Halbwachs *et al*., 2013b; Tello *et al*., 2014). Furthermore, their fruiting is inhibited by the killing of associated vegetation using herbicides (Griffith *et al*., 2014).

Evidence is accumulating that the diversity of mycorrhizal or biotrophic relationships between fungi and plants is greater than previously expected, now including diverse members of Sebacinales, Helotiales, Ceratobasidiales and others (Veldre *et al*., 2013; Behie and Bidochka, 2014; Weiss *et al*., 2016). For example, *Austroboletus* forms mycorrhizal associations with *Eucalyptus* without root penetration (Kariman *et al*., 2014) and *Cortinarius* spp. associate with *Carex* and other non‐shrubby hosts (Harrington and Mitchell, 2002). There is much discussion about whether all these should be considered mycorrhizal (Heijden *et al*., 2015), as for the great majority the reciprocal exchange of nutrients has yet to be demonstrated. Wilson (1995), in attempting to clarify the status of root‐associated fungi, suggested that the term ‘endophyte’ should be applied to fungi for which morphological root adaptations and reciprocal nutrient transfer had not been demonstrated. As the exact nature of the biotrophy of waxcap fungi is unclear, here we use the term endophyte (*sensu* Wilson), where endophytism acts as a symbiotic “waiting room” (as coined by Selosse and Martos, 2014) from which tighter mycorrhizal mutualism may evolve. Indeed, paraphyletic evolution of the ectomycorrhizal habit has occurred within the Hygrophoraceae, with members of *Hygrophorus* forming typical ectomycorrhizas with both deciduous and coniferous hosts (Lodge *et al*., 2014). This, and the phylogenetic position of waxcaps within Hygrophoraceae (Lodge *et al*., 2014), raise the possibility that the common ancestors of *Hygrophorus* and waxcaps were endophytic and that the former evolved the ectomycorrhizal habit following association with trees.

Stable isotope signatures of carbon and nitrogen (expressed as δ^13^C and δ^15^N) have been used to examine the nutritional strategies, trophic position and feeding behaviours of diverse organisms (Boecklen *et al*., 2011). Waxcap basidiocarps exhibit distinctive C and N isotopic natural abundance patterns, being depleted in ^13^C and enriched in ^15^N relative to confirmed saprotrophic macrofungi from the same habitats (Griffith *et al*., 2002; Seitzman *et al*., 2011). Ectomycorrhizal (ECM) basidiomycetes also exhibit ^13^C depletion and ^15^N enrichment relative to saprotrophic species (Taylor *et al*., 1997; Kohzu *et al*., 1999; Taylor *et al*., 2003).

For carbon isotopes, such patterns are explained by ^13^C partitioning within woody plants, with ^13^C preferentially partitioned to plant cellulose relative to soluble sugars, and these representing the primary carbon source for saprotrophic fungi and ECM fungi respectively (Kohzu *et al*., 1999). For nitrogen, ^14^N acquired by ECM fungi from sources in the soil is preferentially transferred to hosts, leading to ^15^N enrichment within the fungal mycelia (Hobbie and Högberg, 2012). However, ^15^N enrichment in basidiocarps varies widely among the diverse groups of ECM fungi. It is also noteworthy that in cases where plants obviously and heavily rely on ECM fungi as nitrogen source (full and partial mycoheterotrophs among the Orchidaceae and Ericaceae), these exhibit ^15^N enrichment rather than depletion (Gebauer and Meyer, 2003; Zimmer *et al*., 2007). Whilst internal isotopic fractionation can partially explain the observed differences in δ^13^C and δ^15^N profiles of basidiocarp tissues, isotopic analyses can additionally provide insight into which sources of C and N are being accessed (Gebauer and Taylor, 1999; Preiss and Gebauer, 2008). Thus we hypothesise that fungi whose tissues are particularly high in δ^15^N derive N from sources which are themselves high in ^15^N. This was shown using paired natural abundance and ^15^N tracer measurements on ectomycorrhizal fungi in a pine forest (Hobbie *et al*., 2014).

An additional method for determining the sources from which macrofungi derive organic carbon is via radiocarbon analysis. This method relies on the spike of atmospheric ^14^CO_2_ resulting from the hydrogen bomb tests of 1957–1963 which declined gradually over the following 50 years to near pre‐bomb levels due to photosynthetic uptake by plants. The steep annual decline in atmospheric ^14^CO_2_ allows accurate estimation of the date at which the carbon present in biological tissues had been fixed by photosynthesis by calibration with ^14^C in atmospheric CO_2_ and plant materials. Hobbie *et al*. (2002) and Chapela *et al*. (2001) have previously used this radiocarbon approach to test the mycorrhizal status of ectomycorrhizal fungi, finding that the ^14^C signature of accepted ectomycorrhizal species was similar to that found in atmospheric CO_2_ 0–2 years previously, whereas the ^14^C signature of saprotrophic species matched that of atmospheric CO_2_ from 2–50 years prior to sample collection.

Apart from the utility of greater understanding of the nutritional biology of waxcaps in conservation efforts, their unusual patterns of ^15^N enrichment (Griffith *et al*., 2002; Seitzman *et al*., 2011) suggests a hitherto unknown trophic strategy which merits further investigation. The negative effects of agricultural soil amendments on waxcap fruiting may be linked to changes in interactions with host plants or other members of the soil biota. In the present study, we present data from fruitbody surveys at a grassland experimental site where several agricultural treatments were applied, alongside isotopic analyses of basidiocarps from a broad range of global habitats, to test the hypothesis that waxcap fungi are mutualistic root endophytes and to determine what sources of soil N they may have accessed.

## Results

### *Effect of fertilizer/lime/biocide additions on waxcap fruiting*

**Figure 1:**
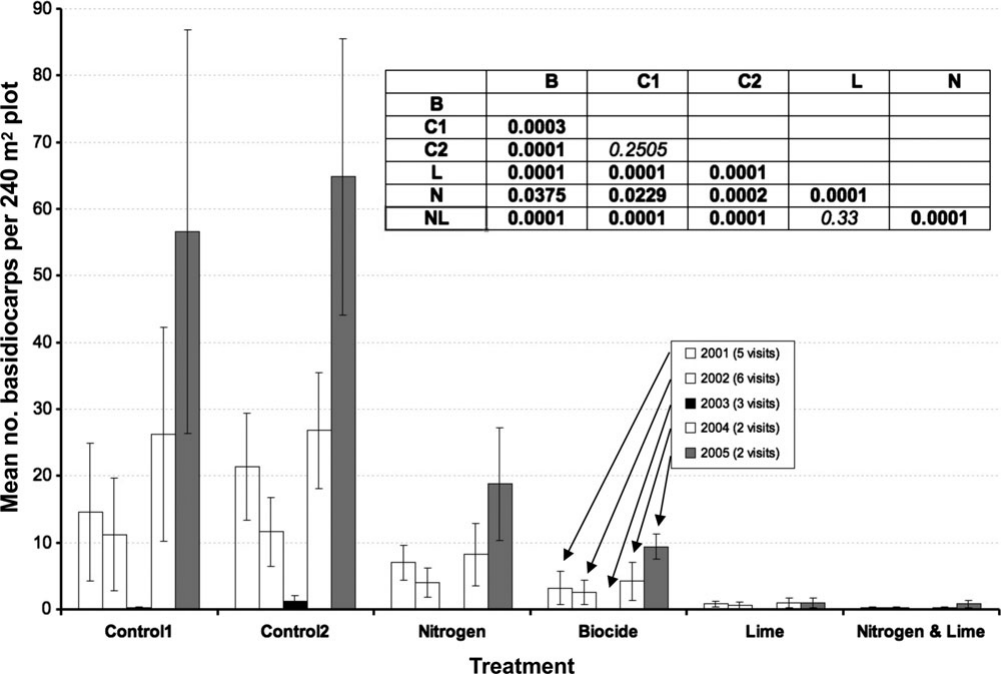
Effect of plot treatment on abundance of basidiocarps at Sourhope over the course of 18 autumn surveys in 2001–2005. (B, Biocide, C1/C2, control plots, L, lime, N, nitrogen and NL, nitrogen + lime; *n* = 5 per treatment). Error bars indicate standard deviation. Inset table shows treatment effect *P* values using PERMANOVA.

The negative effects of synthetic fertilizers on the fruiting of waxcaps have been anecdotally reported (Griffith *et al*., 2004; Boertmann, 2010) but hitherto not rigorously demonstrated. Therefore, the occurrence of waxcap basidiocarps was monitored over a 5‐year period at the Sourhope NERC Soil Biodiversity site. Twelve species of Hygrophoraceae from three genera (*Cuphophyllus* [3], *Gliophorus* [3] and *Hygrocybe* [6]) were found on the Sourhope plots with six additional species found outside plots but within the fenced 1 ha experimental area (Fig. 1; Supporting information S1). *Cuphophyllus pratensis* comprised 63% of the approximately 4000 basidiocarps recorded over the five survey years, followed in abundance by *Gliophorus laetus* (17%) and *Hygrocybe ceracea* (12%).

All treatments clearly affected basidiocarp abundance, with a threefold or greater reduction in basidiocarp abundance relative to controls for addition with nitrogen, lime, chlorpyrifos or nitrogen plus lime (Fig. 1). Fruiting varied widely among years due to climatic variations. Basidiocarp numbers between replicate plots also varied, probably due to the large size and longevity of these organisms; this may have masked any differences in the response of particular species or genera to the experimental treatments.

Whilst synthetic nitrogen fertilizer and the chlorpyrifos (Dursban) biocide were applied at standard agricultural levels, lime was applied at five times the usual rate, leading to a substantial increase in pH on limed plots, from 4.5 in 1999 to > 7 in 2003 (Supporting information S1). As the waxcap species present at Sourhope are also found in calcareous grasslands, the negative effect of lime application is unlikely to be due solely to intolerance of high pH. However, application of lime to acid soils also mobilizes key nutrients, notably P, and also alters core soil processes such as nitrification or nitrogen fixation. Furthermore, the additive effects of nitrogen and lime application (Fig. 1) suggest that the deleterious effect of lime on fruiting related to changes in N cycling in these soils, consistent with the findings of Kuan *et al*.(2006) at this site. Waxcap fruiting was substantially inhibited in plots with added chlorpyrifos.

### *^15^N/^13^C isotopic signatures of soil and vegetation*

**Figure 2:**
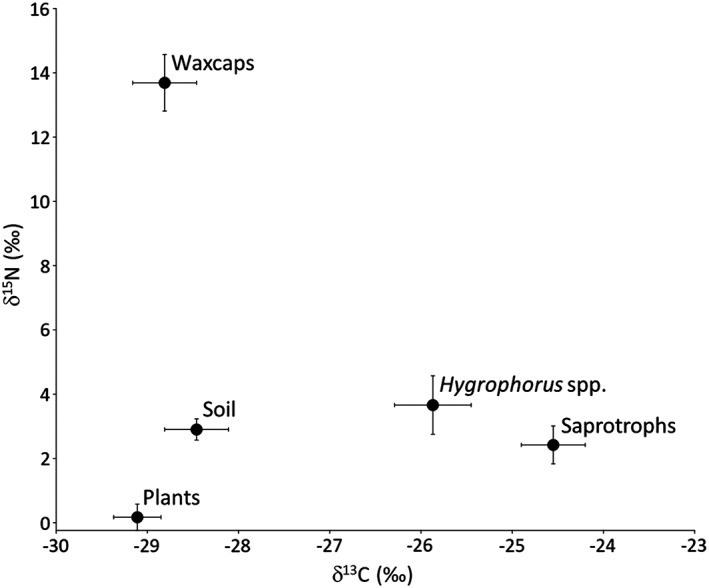
Comparison of δ^15^N and δ^13^C values in waxcap basidiocarps to ectomycorrhizal Hygrophoraceae (*Hygrophorus* spp.) and mean values for saprotrophic fungi at Sourhope and Amorbach. Mean values for soil (0–5 cm) and plant tissues are also shown. Raw isotopic data for basidiocarps is provided in Supporting Information S2 and for soil plants in Supporting Information S3. Error bars indicate standard error.

**Figure 3:**
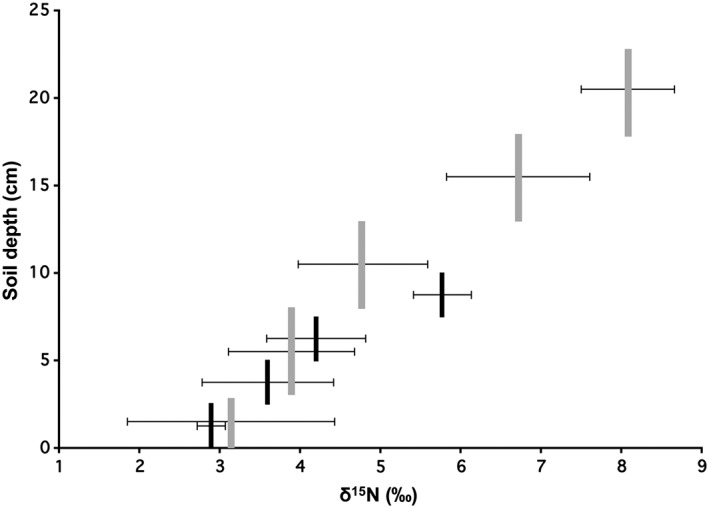
δ^15^N enrichment patterns (‰) along a soil depth gradient. Vertical bars indicate soil depth range sampled at Amorbach (grey bars) and Sourhope (black bars). X‐axis error bars indicate standard deviation.

To provide context for the isotopic profiles of waxcap and other basidiocarps, associated soil and vegetation were collected at Sourhope and Amorbach (the other main survey site) during basidiocarp field surveys. The δ^15^N values for vegetation were all close to 0‰ (−2 to +2‰), significantly lower in ^15^N than basidiocarps, whereas δ^13^C values resembled soil and vegetation values (Fig. 2; Supporting information S2). Soils at Sourhope and Amorbach increased in ^15^N and declined in total N content with greater depth (Figs. 2 and 3; Supporting information S2), following known patterns in higher latitudes (Evans, 2007; Clemmensen *et al*., 2013).

### *Natural abundance δ^15^N in waxcap basidiocarps*

#### *Sourhope*

During field surveys at Sourhope and other sites, all basidiocarps (waxcap and any others) were mapped. Representative samples from each plot and each survey visit were collected and dried, and a subset of these were sent for isotopic analysis. Basidiocarp caps collected from control plots at Sourhope had high δ^15^N values, ranging from 11.7 to 20.6‰ (mean = 15.2 ± 2.2‰; *n* = 42; Table 1; Supporting information S3), substantially greater than that generally found in basidiocarps of other agaric fungi that have been studied, including ECM taxa (Taylor *et al*., 1997; Kohzu *et al*., 1999; Hobbie and Agerer, 2010) but consistent with earlier studies of δ^15^N in waxcaps (Griffith *et al*., 2002; Seitzman *et al*., 2011). Known saprotrophic taxa were much lower in δ^15^N (12 spp.; mean = 1.8 ± 3.3‰; *n* = 17; Supporting information S3), the only sample within the range of the waxcaps being *Agaricus langei* (δ^15^N = 12.5‰).

**Table 1:** Summary of δ13C and δ15N values for waxcap basidiocarps

Species	Ecuador	England	Gabon	Germany (Bavaria)	Guyana	Iceland	Italy (Liguria)	New Zealand	Scotland (Sourhope)	Spain (La Palma)	USA (MA)	Wales
*C. aurantiopallens*								16.7; −29.1 (2)				
*C. canescens*											17.1; −28.5 (1)	
*C. colemanianus*										11.7; −28.6 (1)		
*C. flavipes*				14.4; −30.1 (1)								
*C. fornicatus*				19.7; −30.0 (1)								
*C. aurantiopallens*		9.6; −28.2 (2)									11.9; −28.6 (1)	13.7; −29.6 (1)
*C. muritaiensis*								14.7; −30.7 (1)				
*C. pratensis*		18.6±1.4; −29.3±0.9 (7)		16.2±0.7; −30.0±0.3 (25)		11.6; −30.8 (1)			15.6±2.1; −28.6±0.5 (28)	17.5; −29.5 (1)		15.6±1.1; −29.0±0.7 (11)
*C. virgineus*		15.4±1.0; −28.9±1.2 (4)		14.7±1.4; −29.4±0.9 (85)		12.4; −29.8 (1)	13.7±0.5; −29.0±0.5 (5)	15.7; −30.5 (1)	16.3; −27.7 (1)			13.7±2.0; −28.3±1.5 (9)
*G. laetus*									15.2±2.1 −28.4±0.7 3	14.1; −28.8 (1)	5.1; −27.6 (1)	
*G. lilacipes*								15.5; −30.6 (2)				
*G. luteoglutinosus*								17.6; −25.5 (1)				
*G. perplexus*										15.1; −28.3 (2)		
*G. psittacinus*		18.4; −29.4 (2)		16.0; −28.6 (1)		9.3; −29.7 (1)	17.4±0.4; −29.2±0.3 (4)		17.0±1.8; −27.5±0.3			
*Glio. sp. (HM020676)*											8.4; −27.4 (1)	
*G. versicolor*								13.7; −27.2 (1)				
*G. viridis*								13.6; −28.1 (2)				
*Hu. auratocephalus*											15.0±1.4; −27.2±0.7 (10)	
*Hu. conspicua*								15.9; −27.9 (1)				
*Hu. pura*								17.9; −23.9 (1)				
*Hu. rosella*								12.9; −28.1 (1)				
*Hy. aurantiosplendens*				13.1; −28.9 (1)								
*Hy. blanda*								15.0±5.9; −27.8±0.8 (3)				
*Hy. cantharellus*				**6.1; −31.2 (1)**				**1.8; −31.2 (1)**			**6.6; −28.5 (1)**	**1.2; −28.0 (2)**
*Hy. aurantiosplendens*						7.9; −30.3 (1)			12.3; −27.7 (2)			
*Hy. cerinolutea*								**4.1; −24.5 (1)**				
*Hy. chlorophana*				14.9±0.3; −28.9±0.4 (6)		8.9; −29.2 (2)						14.9±1.9; −28.4±1.3 (6)
*Hy. citrinovirens*		13.7; −28.3 (1)										13.6; −28.9 (1)
*Hy. coccinea*		14.2; −29.0 (1)		13.3±0.9; −30.0±0.8 (32)		9.0; −29.8 (1)				13.4±3.8; −28.1±0.6 (3)		11.9±1.5; −28.9±1.1 (3)
*Hy. conica*						11.4; −29.9 (1)			12.8±1.4; −29.4±0.6 (3)			
*Hy. firma*	11.0; −30.7 (1)							**4.0±1.9; −29.9±1.4 (4)**				
*Hy. glutinipes*												19.5; −28.1 (1)
*Hy. insipida*				12.1; −28.7 (1)						9.6; −28.5 (2)		
*Hy. julietae*								17.5; −28.7 (2)				
*Hy. keithgeorgei*								8.9; −27.7 (2)				
*Hy. lilaceolamellata*								10.2; −26.3 (2)				
*Hy. miniata*								**2.4; −27.0 (1)**		8.5; −28.9 (1)	6.2; −27.7 (1)	
*Hy. mucronella*				19.2; −28.9 (1)								
*Hy. chlorophana*								**5.6; −28.9 (1)**				
*Hy. punicea*		14.6±1.9; −29.3±0.8 (9)				9.7; −29.4 (1)						
*Hy. quieta*						12.6; −30.2 (1)	13.5±0.6; −28.2±0.2 (15)			16.8; −28.3 (2)		16.5; −27.3 (1)
*Hy. reidii*										6.9; −29.8 (1)	8.0±0.8;−28.8±0.8 (3)	
*Hy. rubrocarnosa*								**4.4; −31.0 (2)**				
*Hy. splendidissima*									12.1; −28.4 (1)			
*Hy. sp*.											10.0; −27.8 (1)	
*Hy. sp. (JHN1103)*	18.4; −24.2 (1)											
*Hy. sp. (JHN698)*	13.7; −28.5 (1)											
*Hy. sp. (TU112116)*			8.3; −30.0 (1)									
*Hy. sp. (TU112120)*			11.0; −31.6 (1)									
*Hy. sp. (TU112140)*			21.1; −29.7 (1)									
*Hy. sp. (TU112152)*			21.2; −28.9 (1)									
*Hy. sp. (TU112156)*			18.0; −29.4 (1)									
*Hy. sp. ("toe−head")*					8.0; −28.7 (1)							
*N. nitrata*								9.9; −29.1 (1)		13.6; −29.3 (2)		15.9; −29.5 (1)
*P. calyptriformis*		17.3; −29.5 (2)										15.4±1.2; −28.7±0.9 (6)

Data for 54 species from 12 countries are presented below (across six genera of Hygrophoraceae); Nine samples were not identified to species level). The total of 386 samples include 25 (in italic font) from two published studies (Gabon, Tedersoo et al., 2012; USA, Seitzmann et al., 2011). B16, with replicate number in brackets. Samples with δ15N values below 7.5‰, and below 5‰ are indicated in bold font and light or dark grey respectively.

The basidiocarps analysed included samples from a range of ages (unopened caps to fully mature) but δ^15^N did not differ significantly among different age classes (Supporting information S4a), as has been reported previously for ectomycorrhizal fungi (Taylor *et al*., 1997). As in other agaric fruitbodies (Taylor *et al*., 1997), the ^15^N enrichment was greater in cap tissue compared to stipe (Supporting information S4b), by 2.7 ± 1.7‰. This is consistent with the higher N content of caps compared to stipes (6.8 ± 1.53% N vs. 3.4 ± 0.8) and probably results from the greater protein content of the former, with the N component of the latter mainly present in the structural polymer chitin and glycoproteins.

**Figure 4:**
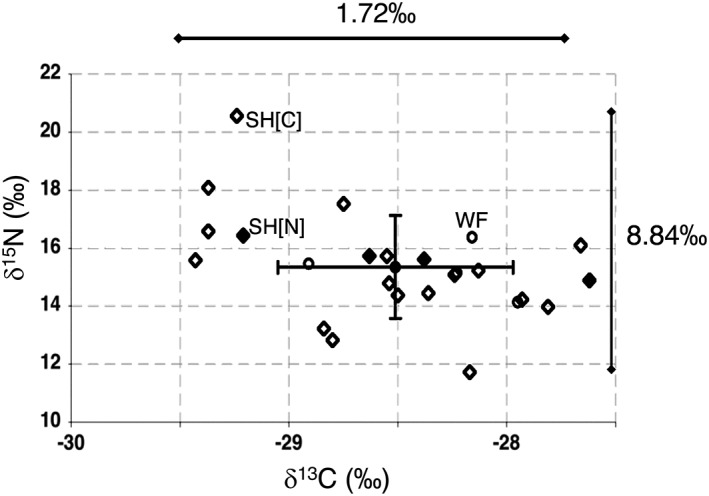
Isotopic profiles of *Cuphophyllus pratensis* are unaffected by fertilizer application (with a δ^15^N value of zero) suggesting that these fungi are unable to utilize inorganic‐N. Open and filled diamond symbols indicate samples from Sourhope, from control (SH [C]) and nitrogen‐treated (SH [N]) plots respectively. Open circles indicate samples from an unfertilised sheep‐grazed meadow in Wales (Waunfawr, Aberystwyth), shown for comparison. The X and Y error bars illustrate standard deviation for all samples. Isotopic profiles of *C. pratensis* at Sourhope were unaffected by fertilizer application.

At Sourhope, a few basidiocarps were found on plots treated with ammonium nitrate (Supporting information S3). As synthetic fertilizer made from N_2_ via the Haber‐Bosch process has a δ^15^N value close to zero (Bateman and Kelly, 2007), it might be expected that basidiocarps formed on these plots would accordingly be lower in δ^15^N. However, *C. pratensis* basidiocarps showed similarly enriched ^15^N profiles compared to those from control plots (mean 15.1 ± 1.8‰ vs. 15.2 ± 2.2‰ respectively; Fig. 4).

#### *Other sites in Europe*

**Figure 5:**
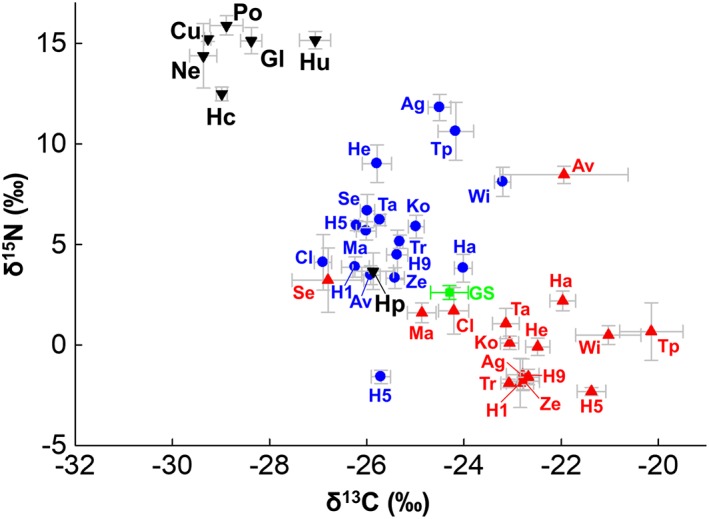
Comparison of intergeneric δ^15^N and δ^13^C variations in Hygrophoraceae genera across all sites and average δ^15^N and δ^13^C for ectomycorrhizal and saprotrophic fungi from 16 published woodland studies. Genera of family Hygrophoraceae are shown with inverted black triangles; Mean for grassland saprotrophic fungi (GS) is indicated with green square. Mean values for ectomycorrhizal fungi (blue circles) and woodland saprotrophic fungi (red triangles) from published studies are also shown. Error bars in grey indicate standard error. Hygrophoraceae genera are labelled as follows: *Cuphophyllus* (Cu), *Gliophorus* (Gl), *Humidicutis* (Hu), *Hygrocybe* (Hc), *Hygrophorus* (Hp), *Neohygrocybe* (Ne), *Porpolomopsis* (Po). Raw data and keys to published study labels (including numbers of replicates) are presented in Supporting Information S10. [Color figure can be viewed at http://wileyonlinelibrary.com]

Similar patterns for isotopic values of waxcap basidiocarps from Sourhope were found in further analysis of samples from upland and lowland grassland sites in Wales, England, Germany, Iceland and Italy (Table 1; Supporting information S3), including a wider range of species (23 species in 5 genera; Fig. 5). All showed very similar patterns of ^15^N enrichment in Wales (14.9 ± 2.0‰; *n* = 36; 10 sites), England (16.3 ± 2.4‰; *n* = 26; 4 sites), Bavaria (14.6 ± 1.7‰; *n* = 155; 2 sites) and Liguria (14.2 ± 1.5‰; *n* = 24; 2 sites) but samples from Iceland were less highly enriched (10.2 ± 1.7‰; *n* = 10; 2 sites). Excluding Iceland, there was consistent ^15^N enrichment, with all but one (*H. cantharellus*, Bavaria; 6.1‰) of the 302 basidiocarps sampled having a δ^15^N signature greater than 10‰ (range 10.9–20.9‰). There was neither significant difference in δ^15^N values for samples from the five genera and 21 species within grassland Hygrophoraceae, nor did grassland type significantly affect δ^15^N (lowland vs. upland).

In Europe, waxcaps are only rarely encountered beyond grassland habitats but four samples from UK woodlands were similarly enriched in ^15^N (14.2 ± 1.9‰; *n* = 4) to grassland samples. However, two samples of *C. lacmus* from a stand of pure heather on Lundy Island were slightly lower in δ^15^N (ca. 9.5‰), whilst two samples of *H. cantharellus* from a boggy upland site vegetated with *Sphagnum* moss had very low δ^15^N values (0.5 and 1.8‰) (Supporting information S3, and 5). In previous work, the ectomycorrhizal genus *Hygrophorus* (Taylor *et al*., 2003; Trudell *et al*., 2004) was much less enriched in ^15^N (all <9.9‰; mean 3.7 ± 4.2‰; *n* = 21; 2 sites) than other Hygrophoraceae. This was the case here for three additional UK and New Zealand *Hygrophorus* specimens.

Saprotrophic taxa from grassland sites across Europe were much lower in δ^15^N (3.0 ± 2.7‰; *n* = 50; Supporting information S3) than waxcaps, consistent with findings for saprotrophs from Sourhope.

#### *Outside Europe*

Species associated with undisturbed grasslands in northern Europe are also found in other continents, usually in woodland habitats. Dried basidiocarp samples were obtained from three areas outside Europe:

- subtropical laurel (laurisilva) montane forest in the Canary Islands (La Palma; dominated by *Laurus novocanariensis* and *Persea indica*, both Lauraceae, with low levels of ground vegetation, mostly ferns, no ground‐dwelling mosses);
- broadleaf‐podocarp forest habitats in New Zealand (Bay of Plenty region, North Island), mostly secondary forest dominated by *Beilschmiedia tawa*, *B. taraire* (Lauraceae), *Cyathea* spp. (Cyatheaceae; tree ferns), *Vitex lucens* (Lamiaceae), *Dysoxylum spectabile* (Meliaceae) or *Leptospermum scoparium* (Myrtaceae), with some remaining canopy trees (*Podocarpus* spp., *Dacrycarpus dacrydoides*, *Dacrydium cupressoides*). (Barton, 1972);
- primary lowland moist evergreen forest in Amazonian Ecuador (Cuyabeno Forest Reserve) (Lodge and Cantrell, 1995);

Published data for waxcaps are also available for two additional sites in Africa (primary lowland rainforest, Gabon; (Tedersoo *et al*., 2012) and coniferous forest/swamp in the United States (Harvard Forest, Massachusetts; (Seitzman *et al*., 2011); data from these studies was also included in our analyses (Table 1).

Across all five areas, δ^15^N values were high for waxcap basidiocarps and mean values were similar to those found in European grasslands: New Zealand (11.16 ± 5.9‰; *n* = 32; range 1.79–20.7‰); La Palma: (13.1 ± 3.7‰; *n* = 16; range 7.0–21.0‰); Ecuador (14.4 ± 3.8‰; *n* = 3; range 11.1–18.4); Gabon: (14.6. ± 6.2‰; (range 10–22‰; *n* = 5 from a 20 ha plot). However, in New Zealand and La Palma, both areas where the vegetation is dominated by non‐ectomycorrhizal Lauraceae, the range of δ^15^N values was particularly high and included some samples where δ^15^N enrichment was much lower than found in European grasslands. These lower (< 10‰) values were all found in members of genus *Hygrocybe* Subgenus *Pseudohygrocybe*, Sections *Coccineae* or *Firmae* [*sensu* (Lodge *et al*., 2014); 3/16 in La Palma and 13/32 in NZ]. Some of these New Zealand *Hygrocybe* spp. had very low δ^15^N (< 2.5; 4/32 samples).

#### *Within‐site variation*

Within the most intensively studied northern European grassland, basidiocarp δ^15^N values varied widely, even for a single species at a single site. For example, δ^15^N values for *C. pratensis* (*n* = 29) at Sourhope varied from 11.7 to 20.6‰ within a 1 ha area. At other sites, δ^15^N varied substantially even within clusters of adjacent basidiocarps across smaller areas (1–2 m^2^): 3.4‰ range for *C. virgineus* (*n* = 20), 3.0‰ for *H. coccinea* (*n* = 16) and 2.0‰ for *H. quieta* (*n* = 15) (Supporting information S3). At Sourhope the position of all *C. pratensis* basidiocarps was accurately mapped, so we tested whether levels of enrichment correlated with topological or other features of the Sourhope site (Supporting information S6). However, no correlations were detected.

### *δ^13^C and ^14^C analyses*

#### *δ^13^C natural abundance*

Waxcap basidiocarps were lower in δ^13^C than basidiocarp cap tissues of ectomycorrhizal and saprotrophic fungi reported here and elsewhere. However, across all samples, δ^13^C values for waxcaps were distributed within a small range (−26.5 to −31.3‰ across all European samples; 4.8‰ range) (Fig. 5; Supporting information S3). The data presented here for more than 20 species in 6 genera and including data from several previous studies show that mean δ^13^C values varied relatively little across the diverse range of habitats [from −27.7 ± 0.9‰ (USA) to −30.0 ± 0.3‰ (Spessart, Germany)]. Samples from New Zealand varied widely in δ^13^C values (−23.9 to −32.1‰) and were over‐represented relative to other areas amongst the more extreme δ^13^C values.

#### *^14^C radiocarbon analysis*

The ^14^C content of three Hygrophoraceae samples were analysed, alongside two samples of saprotrophic fungi (*Agaricus campestris* and *Cystoderma amianthinum*) which commonly co‐occur with waxcaps. Samples collected during the period 1970–1985 (from the RBG Kew Fungarium) were selected, as the steeper gradient then of the atmospheric ^14^C curve allowed more accurate dating. The calibrated midpoint ages for *A. campestris* and *C. amianthinum* were 5.0 and 4.4 years, suggesting that basidiocarp carbon was derived from organic matter formed several years earlier (Table 2). However, for the three Hygrophoraceae, the midpoint ^14^C age was younger, in two cases a few months but for the third it was 1.8 years.

**Table 2:** ^14^C radiocarbon data. Radiocarbon data for UK samples of three Hygrophoraceae and two grassland saprotrophs.

Species	KewlD	Collection date	Location (lat/long)	% Modern	SD	Max age	Min age	Midpoint	δ^13^C
						(year)	(year)	(year)	(‰)
*Cuphophyllus pratensis*	K(M)94710	1‐Oct‐80	Lower Soudley, Forest of Dean (51.7901,‐2.4887)	127.99	0.43	1.5	−1.0	0.3	−28.7
*Cuphophyllus pratensis*	K(M)62869	6‐Sep‐75	Coral Beach, Dunvegan, Skye (57.5001,‐6.6342)	141.63	0.50	2.8	0.9	1.8	−29.2
Porpolomopsis calyptriformis	K(M)60408	18‐Nov‐82	Down House, Kent (51.3312, 0.0533)	124.34	0.54	2.2	−0.9	0.7	−28.9
*Agaricus campestris*	K(M)131768	15‐Sep‐84	Redlynch, Salisbury (50.9905, −1.7113)	129.28	0.39	5.8	4.2	5.0	−24.7
*Cystoderma amianthinum*	K(M): 101520	26‐Oct‐75	Armanthwaite, Cumbria (54.8346, –2.7870)	150.38	0.41	5.1	3.8	4.4	−23.6

## Discussion

### *Host range of waxcap fungi*

In Europe, waxcap fungi are predominantly associated with grassland habitats (Halbwachs *et al*., 2013a). This partly relates to the unusual nature of European grasslands, which are largely sub‐climax ecosystems, in which succession to woodland has been prevented by human activity or large mammalian herbivores (Vera, 2000), whereas afforestation of grassland ecosystems in other continents is prevented by low or episodic rainfall. In the temperate and boreal systems dominated by trees which host ECM fungi, waxcaps are encountered only rarely (Hesler and Smith, 1963). However, forest systems at lower latitudes are generally dominated by non‐ECM tree species and waxcaps are widespread in such habitats. In the present study, many samples were obtained from forests dominated by non‐ECM (and predominantly AM‐associated; Wang and Qiu, 2006; Tedersoo and Nara, 2010) Lauraceae in New Zealand and the Canary Islands. Quantitative data was not obtained but waxcaps are clearly more commonly encountered here than in more northerly ECM‐dominated habitats. Toju *et al*. (2014) detected several *Hygrocybe* spp. in Japanese forests containing significant amounts of Lauraceae (19%; *Neolitsia*, *Litsea*, *Machilus*), all from the roots of non‐fagaceous (non‐ECM) plants. Thus waxcaps appear to avoid ectomycorrhizal plants (Halbwachs *et al*., 2013a), a phenomenon also seen in some European grasslands when ectomycorrhizal shrubs such as *Helianthemum* are present (Griffith *et al*., 2013). If mycorrhizal, then these observations suggest that waxcaps cannot associate with ECM hosts or cannot compete with ECM fungi.

The absence of any consistent group of host plants, in contrast to ECM and ericaceous mycorrhizal fungi, is the main reason that waxcaps have been classified as saprotrophs. Suggestions of their putative hosts have often pointed to bryophytes (Griffith *et al*., 2002; Seitzman *et al*., 2011; Lodge *et al*., 2014), as some waxcaps, mostly in *Hygrocybe* Section *Cocciniae* (Lodge *et al*., 2014), commonly associate with *Sphagnum*, especially in boreal locations (Boertmann, 2010). The lowest δ^15^N (0–2‰) values for European waxcaps were for two *H. cantharellus* samples in *Sphagnum*. Several of the *Hygrocybe* samples analysed by Seitzman *et al*. (2011), also from a site dominated by *Sphagnum,* were also low in δ^15^N (6.2–7.4‰). *H. cantharellus* is found with *Sphagnum* in New Zealand (http://www.kaimaibush.co.nz/Fungi/Hygrocybe1.html) and several *Hygrocybe* spp. from this area had unusually low δ^15^N, however, precise substrate details are not available for our samples. Thus the association of certain *Hygrocybe* spp. with *Sphagnum*, and the low δ^15^N values for these at diverse locations, suggests a biotrophic interaction atypical of those more commonly found amongst waxcaps. This may relate to the important role of cyanobacteria in the nitrogen nutrition of *Sphagnum* (Berg *et al*., 2013).

Mosses are also abundant in many European ‘waxcap’ grasslands, with *Rhytidiadelphus squarrosus* being the second most commonly associated species (after *Agrostis capillaris*) in Welsh grasslands (Griffith *et al*., 2014). At Sourhope, this moss comprised 17% of total cover in control plots (Supporting information S1), however, at other waxcap‐rich grasslands in the UK, for example Park Grass (Silvertown *et al*., 2006), mosses are present at only low abundance, as they are from laurisilvan forests and tropical wet forests. Killing of mosses with FeSO_4_ did not affect waxcap fruiting (Griffith *et al*., 2014). In laurel forests at La Palma where waxcaps are commonly found, mosses are largely absent. Taken together these lines of evidence suggest that the putative hosts may include both mosses and a potentially diverse array of higher plants.

### *The biotrophic status of waxcap fungi*

The additional δ^13^C natural abundance data presented here confirm for many waxcap species from diverse habitats across the world that the basidiocarps of these fungi are consistently more depleted in ^13^C (mostly −26 to −30‰) than are those of ECM (Taylor *et al*., 1997; Taylor *et al*., 2003). They are also depleted in ^13^C relative to saprotrophic taxa examined here from grassland habitats across the UK (−24.9 ± 1.6‰), as well as saprotrophs from other, mostly woodland, habitats (Kohzu *et al*., 1999; Taylor *et al*., 2003). This is consistent with the possibility that waxcaps, like other members of the Hygrophoraceae, are biotrophs deriving organic C from plant hosts rather than from soil organic matter.

We did not undertake extensive analyses of associated soil and vegetation but at Sourhope δ^13^C values for the dominant plant *Agrostis capillaris* (mean leaf and root δ^13^C –27.8‰ and − 27.3‰, respectively; *n* = 3; Supporting information S6) and soil (−26 to −27‰ at Sourhope and increasing with soil depth; Figs. 2 and 3; Supporting information S6) were depleted in ^13^C relative to basidiocarps of saprotrophs at Sourhope (−25.1 ± 1.4‰; *n* = 43), consistent with the preferential loss of ^12^C as CO_2_ during microbial catabolism of organic matter (Kohzu *et al*., 1999). In contrast, mean δ^13^C values for waxcap tissues at Sourhope (−28.4 ± 0.7‰) were, however, similar to vegetation and slightly depleted in ^13^C relative to soil by ca. 2‰. If waxcaps are saprotrophic, it is difficult to explain why they are depleted in ^13^C relative to soil, and distinct from known saprotrophs. Thus the most parsimonious explanation for the ^13^C natural abundance patterns reported here is that that they derive C directly from plant photosynthate.

We used the steep annual decline in atmospheric ^14^CO_2_ during the 1970–1980s to estimate accurately the date at which the carbon present in fungarium basidiocarps had been fixed by photosynthesis. The ^14^C age of the waxcap samples analysed was 0–2 years, whereas for two saprotrophic grassland fungi, the ^14^C age was 4–5 years. These ^14^C dates for waxcaps are consistent with the use of recently fixed C such as photosynthate, and not consistent with the use of soil organic matter which contains C fixed many years earlier (Chapela *et al*., 2001; Hobbie *et al*., 2002). Fungal colonization of senescent or recently dead roots cannot be excluded, since this would also have a recent ^14^C signature. Hobbie *et al*. (2002) found that fresh conifer needles were unexpectedly enriched in ^14^C, dating to 0–3 years prior to collection, and they discussed the various factors that could account for both this and the similar dating of ^14^C in basidiocarps of known mycorrhizal species, including anaplerotic fixation of CO_2_ (Wingler *et al*., 1996), internal recycling of C within mycelial systems and uptake of C from soil amino acids by fungi. However, Treseder *et al*. (2006) found no evidence of transfer of ^14^C from labelled litter to ECM fungi.

As part of the NERC Soil Biodiversity Initiative, a ^13^CO_2_ pulse labelling facility was established at Sourhope during 2000–2002 (Staddon *et al*., 2003). We collected basidiocarps formed near the pulse points to test whether any ^13^CO_2_ fixed by plants within the pulse domes was later incorporated into waxcap basidiocarps formed nearby. A single heavily‐labelled basidiocarp of *C. pratensis* (δ^13^C 92.3‰; Supporting information S7) was detected 20 cm from one pulse dome (14 days after the 4–6 h pulse). Johnson *et al*. (2002) established in‐growth cores (with root but not hyphal in‐growth prevented by 35 μm mesh) within the pulse domes and showed that ^13^CO_2_ from microbial respiration within the cores was significantly reduced if cores had been rotated to disrupt hyphal connections. They ascribed this respiration to arbuscular mycorrhizal fungi (AMF) but this respiration could also have been by mycelia of waxcaps, a possibility supported by DNA metabarcoding studies which consistently find AMF abundance (< 5%) in undisturbed grasslands to be lower than that of waxcaps and other fungal groups (Geml *et al*., 2014; Jumpponen and Jones, 2014; Detheridge *et al*., 2018). Treonis *et al*. (2004) used the same pulsing system to monitor incorporation of ^13^C from labelled CO_2_ into microbial phospholipid fatty acids (PLFAs). The most heavily labelled PLFA was 18:2ω6,9 which is the dominant PLFA in basidiomycetes (> 80% of total PLFAs) and present at only low abundance (< 2%) in AMF (Baldrian *et al*., 2013). These observations are consistent with the other isotopic data presented above, suggesting that waxcaps can access recently fixed photosynthate.

### *Isotopic enrichment of ^15^N in waxcap basidiocarps*

The consistently high level of ^15^N enrichment reported here (Table 1), significantly higher than values for soil and vegetation which we also analysed (Fig. 4; Supporting information S6), is not found in any other group of basidiomycetes, though similarly high values are found for some ascomycetes (*Tuber* spp.) associated with mycoheterotrophic orchids (Schiebold *et al*., 2017). More than > 40% of the > 250 samples reported here were enriched beyond 15‰ and most samples below 10‰ are either associated with *Sphagnum* or are within *Hygrocybe* Section *Cocciniae*, as described above. ECM basidiocarps enriched in δ^15^N are widely reported (mostly falling within the range + 3 to +9‰), however, values above 10‰ are unusual (Trudell *et al*., 2004). There has been much discussion about why ECM basidiocarps are enriched in ^15^N, and also why ECM fungi belonging to different genera are variably enriched in ^15^N relative to atmospheric N_2_ and the bulk substrate (Trudell *et al*., 2004; Hobbie and Agerer, 2010). One probable factor for some ^15^N enrichment in ECM fungi is the preferential supply of lighter ^14^N to the host due to fractionation against ^15^N during transamination (glutamine–glutamate shuttle) at the mycorrhizal interface (Emmerton *et al*., 2001; Hobbie *et al*., 2012). Such transamination reactions discriminate strongly against ^15^N (Handley and Raven, 1992).

Transamination from glutamine is also involved in chitin synthesis (via glucosamine:fructose‐6‐phosphate aminotransferase; EC2.6.1.16; (Ram *et al*., 2004) and the fractionation against ^15^N during glucosamine synthesis explains the lower ^15^N enrichment of more chitin‐rich stipe tissues in waxcaps (by 0.7–5.4‰; Supporting information S4), relative to the more protein‐rich cap tissues. Similar differences in δ^15^N between caps and stipes are reported for basidiocarps of many other fungi including ECM (Taylor *et al*., 1997; Hobbie *et al*., 2012). Although the N content of chitin is low (6.3%) compared to protein (ca. 15%), it comprises a significant proportion of total biomass in filamentous fungi (5%–10% of cell dry weight; (Free, 2013) and thus represents an important pool of N. The dynamics of chitin catabolism and internal recycling in fungi are not well understood (Merzendorfer, 2011) but recent genome data can provide valuable insights. For example, the model ECM species *Laccaria bicolor* contains genes encoding chitinase/hexosaminidase (EC3.2.1.14/EC3.2.1.52) which mediates release of N‐acetylglucosamine monomers but it lacks the requisite genes for further breakdown [N‐acetylglucosamine‐6‐phosphate deacetylase (EC3.5.1.25) and glucosamine‐6‐phosphate deaminase (EC3.5.99.6)] which are present in the genomes of most saprotrophic fungi hitherto studied (http://www.genome.jp/kegg). This suggests, for this species at least, that once incorporated into chitin, this N is not released back to the protein/inorganic pool.

To explain the wide range of taxon‐specific δ^15^N values in the basidiocarps of ECM fungi, Hobbie and Agerer (2010) correlated these differences with the nature and extent of the underlying mycelial system (http://www.deemy.de; (Rambold and Agerer, 1997). Taxa having more extensive exploratory mycelia formed basidiocarps which were more enriched in ^15^N (Supporting information S8). They postulated that the wide variation in δ^15^N values could be explained using a two‐pool model of distribution of N within mycelial systems, one comprising immobile ^15^N‐depleted chitin and the other mobile ^15^N‐enriched protein, as described above. They proposed that the loss of ^15^N‐depleted N to the host plant and its immobilization in chitin leads to ^15^N enrichment of the protein pool which is translocated for basidiocarp formation (Supporting information S8). Thus, high δ^15^N values for basidiocarps could be explained by high levels of transfer of N to hosts or by high investment in cell wall material (i.e. extensive mycelial systems). Whether this is a feature of all mycorrhizal association is unclear as both (Merckx *et al*. (2010) and Courty *et al*. (2015) did not find evidence of such ^15^N partitioning in AMF symbioses.

Recent DNA metabarcoding analysis of undisturbed grasslands in the UK has shown that waxcaps alongside Clavariaceae comprise 40%–70% of the total fungal biomass present in these soils, with waxcap mycelia present at ca. 0.5–1.5 mg.g^−1^ dw. soil (Detheridge *et al*., 2018). This is consistent with observations of large numbers of waxcap basidiocarps, sometimes forming large fairy rings (Griffith *et al*., 2014). In contrast, ECM‐forming Hygrophoraceae (*Hygrophorus* spp.) form only limited mycelial networks (http://www.deemy.de; (Agerer, 2006), consistent with their low δ^15^N signatures (Seitzman *et al*., 2011).

For some ECM, ^15^N enrichment of basidiocarps has been linked to ^15^N depletion in tissues of the host plants (Högberg *et al*., 1996; Hobbie and Högberg, 2012). However, this may prove challenging to demonstrate for waxcaps due to their apparently diverse host associations and the absence of any visible soil mycelial networks (Halbwachs *et al*., 2013b). Nevertheless, δ^15^N values for European waxcaps were 12.5‰ higher than those of saprotrophs inhabiting the same grasslands. The latter obtain their nitrogen from plant litter and soil with lower ^15^N content (Gebauer and Taylor, 1999) and the disparity suggests that waxcaps use nitrogen sources different from those accessed by saprotrophic fungi.

### *N sources accessed by waxcaps*

It has long been suggested that one reason for the drastic reduction in the numbers of ‘waxcap’ grasslands in Europe is the widespread use of synthetic fertilizers in agriculture. However, this has not hitherto been rigorously demonstrated. At the Sourhope field experiment, application of ammonium nitrate at standard agricultural rates caused a threefold reduction in the abundance of waxcap basidiocarps and even greater reduction when applied in conjunction with lime (Fig. 1).

The fact that these differences were consistently found over a five‐year period suggests that such changes are linked to long term decline of fungal mycelium in soil. ECM fruiting declined along a nitrogen deposition gradient (< 15 kg N.ha^−1^ years^−1^), (Lilleskov *et al*. (2001, 2002) with the more nitrogen‐sensitive species, for example *Cortinarius* spp., preferentially using organic N sources. The authors also observed that basidiocarp δ^15^N values were positively correlated with mineral N levels in soil. However, our data did not reveal any such correlation, with δ^15^N values of waxcap basidiocarps similar for N‐amended plots and from control plots (Fig. 4). As noted above, the species examined by Lilleskov *et al*. (2002) were all culturable and able to use ammonium as sole N source. Waxcaps remain uncultured, so it has not been possible to test directly their ability to utilize inorganic N, though our field data suggest not. Nitrate or ammonium (or the redox intermediates of these) could be toxic to waxcaps but it is also possible that other soil microbes out‐competed waxcaps for ammonium and nitrate in the amended plots. The synergistic inhibition by nitrogen and lime on waxcap fruiting points towards out‐competition for resources by bacterial populations, as the ratio of fungi to bacteria (F:B ratio; (Bardgett *et al*., 1996) is known to correlate positively with pH. Dawson *et al*. (2003) reported a fourfold increase in bacterial numbers on N/L plots compared to control plots at Sourhope, whereas populations of culturable fungi remained constant, as did fungal PLFA levels (Murray *et al*., 2006).

The fractionation mechanisms described above and the Hobbie/Agerer two‐pool model provide only a partial explanation of the high δ^15^N values of waxcap basidiocarps. The lines of evidence set out above suggest that waxcaps rely on sources of organic N. Bulk SOM at Sourhope and Amorbach were enriched in ^15^N (6–8‰; Figs. 2 and 3), with the level of enrichment increasing with soil depth (linked to a decrease in total N content). It is possible that waxcaps access N from aged/recalcitrant ^15^N‐enriched sources, such as complex heterocyclic polymers in deeper soil horizons (Dijkstra *et al*., 2006; Evans, 2007; Hobbie and Ouimette, 2009). Such sources would at least partly originate from organisms high in the food chain: the higher the ^15^N signature of an organism, the higher its position in the food chain (Griffith, 2004; Boecklen *et al*., 2011). We consider it highly unlikely that N is obtained from any plants, since these have consistently low δ^15^N values (−3 to +5‰; Supporting information S2; (Temperton *et al*., 2012). However, more plausible alternate sources could also include microbe‐derived N in the form of highly recycled amino acids, amino sugars or protein‐N continuously recycled and mopped up by microbes (as N fragments or whole amino acids) such that the lifetime of these labile compounds in soils is thereby extended (Gleixner *et al*., 2002; Bol *et al*., 2009). Such an argument would explain their occurrence only in undisturbed grasslands where soil processes over decadal timescales have accumulated old organic matter reserves in deeper soil strata. However, this explanation ignores the higher organic N content towards the upper, more accessible surface horizons.

The fivefold reduction in waxcap fruiting at Sourhope following application of the pesticide chlorpyrifos offers potential insight into the N metabolism of waxcaps. This acetylcholinesterase inhibitor is toxic to invertebrates, with for instance earthworms exhibiting sublethal effects at standard application rates (Pelosi *et al*., 2014). There is no evidence of direct inhibition of fungal growth/differentiation by chlorpyrifos at the levels applied here (338 mg.m‐^2^.year^−1^; Supporting information S1), so this finding suggests a hitherto undescribed interaction between waxcaps and soil animals. Dawson *et al*. (2003) quantified microbial PLFA at Sourhope and reported a substantial (sevenfold) decrease in fungal biomass on biocide plots. This raises the possibility that waxcaps may derive N from soil invertebrates and are inhibited in fruiting (and possibly mycelial growth) by the absence of such food sources. Several agaric fungi can derive N from invertebrates (Barron and Thorn, 1987), including the ectomycorrhizal *Laccaria laccata*, which can consume collembolans and transfer N from such sources to host plants (Klironomos and Hart, 2001). Similarly, *Metarrhizium* spp. and other claviceptaceous entomopathogens can transfer insect‐derived N to diverse host plants (Behie *et al*., 2012).

Nitrogen derived from soil animals may also explain not only the highly elevated δ^15^N values of grassland waxcap basidiocarps but also the high level of localized variability of these δ^15^N patterns. At Sourhope, δ^15^N values for *C. pratensis* across control plots varied by 8‰ (12–20‰), similar to that found for this species at a global scale. Even for basidiocarps forming part of the same fairy ring there was 3–4‰ variation (Supporting information S3), suggesting a high level of spatial heterogeneity in the N sources accessed. The only known insect‐associated fungus at Sourhope was *Cordyceps militaris*, a pathogen of lepidopteran larvae. Ascocarps of this species from control plots at Sourhope were high and variable in δ^15^N (8.0 ± 2.4‰, n = 3; Supporting information S3).

A meta‐analysis of δ^15^N values in soil invertebrates by Tiunov (2007) suggested that many are elevated in ^15^N, ranging from +6.5 to +9.3‰, with predators occupying higher trophic levels in food webs being more enriched in ^15^N (Supporting information S9). With regards to which invertebrates might be the source of waxcap N, surveys of soil invertebrates in biocide plots at Sourhope found severe effects on certain groups (e.g. tipulids) and lesser negative effects on other taxa (enchytraeids, nematodes, tardigrades) (Dawson *et al*., 2003; Cole *et al*., 2006). The most abundant invertebrates are earthworms and dipteran larvae, which together typically accounted for >70% of total animal biomass (Murray *et al*., 2009). Bishop *et al*. (2008) found the endogeic species *Allolobophora chlorotica* to be the most abundant earthworm at Sourhope (40 individuals.m^−2^ on control plots), whilst Neilson *et al*. (2000) found this species to be the most highly enriched in ^15^N (8–10‰) of all the earthworm species at several of the grassland sites they studied. Similarly, Schmidt *et al*. (2004) found that this and other endogeic earthworms and Enchytraeidae were the most highly enriched in ^15^N (11–15‰; Supporting information S9).

We estimated invertebrate biomass in Sourhope control plots using published data (earthworms, collembolans, enchytraeids; Table 1 in (Cole *et al*. [2006] and tipulids; Fig. 1 in (Dawson *et al*. [2003]) to be ca. 100–200 g fresh weight m^−2^, accounting for the substantial (>2‐fold) seasonal variation that was recorded. This corresponds to 1.75–3.5 g N.m^−2^ (assuming 83% water content and 10% N content of dw), ca. 1% of the total N pool [for Sourhope soils (0–10 cm; 60 kg dry weight.m^−2^ at 0.4% N) =240 g N.m^−2^]. We further estimated the total N content in waxcap biomass to be ca. 3 g.m^−2^ [based on 0.25 basidiocarps.m^−2^, with dry wt 2.5 g each (*C. pratensis*) and 5% N content (Supporting information S3), assuming 1% of biomass in basidiocarp]. These estimates suggest that the likely N demand from waxcaps and the N available via soil invertebrates are of similar magnitude.

### *Conclusions*

Here, we provide evidence from isotopic studies and field experiments that not only do these fungi obtain C directly from plant photosynthate but that they are potentially mycorrhizal. The diversity of habitats occupied by waxcap fungi suggests that they can form associations with a diverse range of host plants and their absence from habitats dominated by ECM suggests that they are outcompeted in such habitats. The N nutrition of waxcaps is clearly unusual but their elevated δ^15^N signatures were remarkably conserved at a global level, with rare exceptions restricted to a few species within genus *Hygrocybe* and possible association with bryophyte hosts. Within the context of their elevated δ^15^N signatures, we were unable to pinpoint the cause of the high variability in δ^15^N but we propose that this is linked to their ability to utilize diverse N sources which vary in δ^15^N, and which include soil invertebrates. However, it is also possible hitherto undiscovered uptake or loss processes leading to extreme levels of ^15^N fractionation may be involved. In the absence of any axenic/monoxenic culture system for waxcaps, it is likely that further elucidation of the mechanisms involved must be obtained from genomic data and compound‐specific isotopic investigations.

## Experimental methods

### *Field sites and plot treatments at Sourhope*

An upland grassland (Rigg Foot, Sourhope, Kelso, Scotland; Lat/Long: 55.470°, −2.231°; altitude: 310 m; the focus of the NERC Soil Biodiversity Programme) was the main field site used in this study. The site had previously been under pasture for at least 50 years with no fertilizer additions and is classified as U4d (*Festuca‐Agrostis‐Galium*) in the UK National Vegetation classification (Supporting information S1). The soils are acid brown earths, derived from andesitic lavas of Old Red Sandstone.

The experiment comprised four treatments (five replicates each; 12 × 20 m plots): Control (C; no additions), nitrogen fertilizer [N; granular NH_4_NO_3_ (24 g.m^−2^. years^−1^), two equal applications in May and July]; lime (L; 600 g.m^−2^, each spring); biocide [B; Dursban 4 (Chlorpyrifos), five monthly application from May to September (0.15 ml.m^−2^)]; Nitrogen/Lime (NL; as above combined) (Fitter *et al*., 2005). Grass was cut to 6 cm at 3 week intervals (six cuts from May to September) and clippings were removed (Supporting information S1). Basidiocarp surveys were conducted over a five‐year period (2001–2005) with 2–5 visits annually (12–26 days apart) between early September and early November. Names used for fungal species are in agreement with IndexFungorum.

### *Sampling and isotopic analyses*

Isotopic analyses were conducted on cap (pileus) tissues of dried basidiocarps. Samples observed during field surveys were picked, dried within 24 h of collection in a ventilated drying cabinet at <40°C and stored desiccated at the Aberystwyth University Fungarium (code ABS). Identification of samples was based on macroscopic and microscopic examination according to Boertmann (2010). Subsamples of cap tissue were excised from these and ground for isotopic analysis. Further details of sampling locations and the species analysed are given in Supporting information S6. At two sites (Amorbach and Sourhope), soil cores were taken in a W‐shaped pattern (30 cm distance between cores; 3–5 replicates). Samples were separated by depth into 25–30 mm segments down to the bedrock (Sourhope) or up to 23 cm (Amorbach). Cores were dried overnight at 60 °C and ground to a fine powder. Samples of associated higher plants were also collected adjacent to soil cores, dried and ground.

Isotope analyses were conducted by continuous flow‐isotope ratio mass spectrometry (CF‐IRMS), using an automated N/C analysis‐mass spectrometry (ANCA‐MS) system (Europa 20/20, Crewe, UK) at Lancaster, North Wyke, by Conflo III Interface. Recent samples from Italy, Liguria, La Palma and northern Bavaria were analysed at the Bayreuth University (for details refer to http://www.bayceer.uni-bayreuth.de/ibg/en/ausstattung/geraet/geraet_detail.php?id_obj=65905). Values were referenced against atmospheric nitrogen and V‐PDB limestone standards.

### *^14^C analysis*

To obtain basidiocarp samples corresponding to a period when atmospheric radiocarbon (^14^C) levels were elevated, three Hygrophoraceae samples were chosen from the Mycological Herbarium at the Royal Botanic Gardens (RBG), Kew. Samples were taken from grassland sites but avoided urban areas, where the ^14^C‐dead CO_2_ from localized fossil fuel burning could cause distortion of data. The samples were analysed to quantify ^14^C content. Basidiocarp cap segments were subjected to a weak acid wash (0.5 M HCl) to remove potential contaminants. The samples were then combusted in sealed quartz tubes and sample CO_2_ cryogenically recovered, graphitised (Slota *et al*., 1987) and measured for ^14^C concentration by accelerator mass spectrometry (AMS) at the Scottish Universities Environmental Research Centre, East Kilbride, UK. Following convention, ^14^C results were normalized to a δ^13^C of −25‰, and expressed as percentage modern (ratio of sample ^14^C content relative to the oxalic acid international standard; (Stuiver and Polach, 1977). Radiocarbon concentrations were used to determine the age of initial carbon fixation from the atmosphere by matching sample percentage modern results with a record of atmospheric ^14^C content over the last 60 years. This calibration was performed using ‘Calibomb’ software (Reimer *et al*., 2004) using the northern hemisphere atmospheric ^14^CO_2_ dataset (Levin *et al*., 2008).

## Supporting information


**Suppdata 1.** Details of Sourhope surveys
**Suppdata 2.** δ^13^C and δ^15^N values of soils and vegetation at Amorbach and Sourhope.
**Suppdata 3.** Sampling locations for basidiocarp isotope analyses
**Suppdata 4.** δ^15^N / δ^13^C in basidiocarps of different ages between cap vs. stipe tissues.
**Suppdata 5.** Images of *C. lacmus* and *H. cantharellus* growing in heather/moss
**Suppdata 6.** Spatial distribution of *C. pratensis* and *C. virgineus* basidiocarps at Sourhope
**Suppdata 7**. Data from the seven waxcap Basidiocarps close to pulse sites
**Suppdata 8**. Summary of Hobbie and Agerer theoretical pattern of N isotope fractionation in mycorrhizal systems
**Suppdata 9**. δ^15^N values in soil invertebrates from published studies
**Suppdata 10**. Raw data and references for published studies relating to Fig. 3

## References

[emi14327-bib-0001] Agerer, R. (2006) Fungal relationships and structural identity of their ectomycorrhizae. Mycol Prog 5: 67–107.

[emi14327-bib-0002] Baldrian, P. , Větrovský, T. , Cajthaml, T. , Dobiášová, P. , Petránková, M. , Šnajdr, J. , and Eichlerová, I. (2013) Estimation of fungal biomass in forest litter and soil. Fungal Ecol 6: 1–11.

[emi14327-bib-0003] Bardgett, R. D. , Hobbs, P. J. , and Frostegard, A. (1996) Changes in soil fungal: bacterial biomass ratios following reductions in the intensity of management of an upland grassland. Biol Fertil Soils 22: 261–264.

[emi14327-bib-0004] Barron, G. L. , and Thorn, R. G. (1987) Destruction of nematodes by species of *Pleurotus* . Can J Bot 65: 774–778.

[emi14327-bib-0005] Barton, I. L. (1972) On the vegetation of the Hunua ranges, Auckland. N Z J Bot 10: 8–26.

[emi14327-bib-0006] Bateman, A. S. , and Kelly, S. D. (2007) Fertilizer nitrogen isotope signatures. Isot Environ Health Stud 43: 237–247. 10.1080/10256010701550732 17786669

[emi14327-bib-0007] Behie, S. , Zelisko, P. , and Bidochka, M. (2012) Endophytic insect‐parasitic fungi translocate nitrogen directly from insects to plants. Science 336: 1576–1577. 22723421 10.1126/science.1222289

[emi14327-bib-0008] Behie, S. W. , and Bidochka, M. J. (2014) Nutrient transfer in plant–fungal symbioses. Trends Plant Sci 19: 734–740. 25022353 10.1016/j.tplants.2014.06.007

[emi14327-bib-0009] Beisenherz, M. (2000) Untersuchungen zur Ökologie und Systematik der Gattung *Hygrocybe* (Agaricales). Diss Univ Regensburg Auszüge, Regensb Mykol Schr 10: 3–65.

[emi14327-bib-0010] Berg, A. , Danielsson, Ö. , and Svensson, B. H. (2013) Transfer of fixed‐N from N_2_‐fixing cyanobacteria associated with the moss *Sphagnum riparium* results in enhanced growth of the moss. Plant Soil 362: 271–278.

[emi14327-bib-0011] Bishop, H. O. , Grieve, I. C. , Chudek, J. A. , and Hopkins, D. W. (2008) Liming upland grassland: the effects on earthworm communities and the chemical characteristics of carbon in casts. Eur J Soil Sci 59: 526–531.

[emi14327-bib-0012] Boecklen, W. J. , Yarnes, C. T. , Cook, B. A. , and James, A. C. (2011) On the use of stable isotopes in trophic ecology. Annu Rev Ecol Evol Syst 42: 411–440.

[emi14327-bib-0013] Boertmann, D. (2010) The Genus Hygrocybe, 2nd ed: Danish Mycological Society, Tilst, Denmark.

[emi14327-bib-0014] Bol, R. , Gleixner, G. , Poirier, N. , and Balesdent, J. (2009) Molecular turnover time of SOM in particle size fractions of an arable soil. Rapid Commun Mass Spectrom 23: 2551–2558. 19603490 10.1002/rcm.4124

[emi14327-bib-0015] Clemmensen, K. , Bahr, A. , Ovaskainen, O. , Dahlberg, A. , Ekblad, A. , Wallander, H. , *et al* (2013) Roots and associated fungi drive long‐term carbon sequestration in boreal forest. Science 339: 1615–1618. 23539604 10.1126/science.1231923

[emi14327-bib-0016] Cole, L. , Bradford, M. A. , Shaw, P. J. , and Bardgett, R. D. (2006) The abundance, richness and functional role of soil meso‐and macrofauna in temperate grassland—a case study. Appl Soil Ecol 33: 186–198.

[emi14327-bib-0017] Courty, P.‐E. , Doubkova, P. , Calabrese, S. , Niemann, H. , Lehmann, M. F. , Vosatka, M. , and Selosse, M.‐A. (2015) Species‐dependent partitioning of C and N stable isotopes between arbuscular mycorrhizal fungi and their C3 and C4 hosts. Soil Biol Biochem 82: 52–61.

[emi14327-bib-0018] Chapela, I. H. , Osher, L. J. , Horton, T. R. , and Henn, M. R. (2001) Ectomycorrhizal fungi introduced with exotic pine plantations induce soil carbon depletion. Soil Biol Biochem 33: 1733–1740.

[emi14327-bib-0019] Dawson, L. A. , Grayston, S. J. , Murray, P. J. , Cook, R. , Gange, A. C. , Ross, J. M. , *et al* (2003) Influence of pasture management (nitrogen and lime addition and insecticide treatment) on soil organisms and pasture root system dynamics in the field. Plant Soil 255: 121–130.

[emi14327-bib-0020] Detheridge, A. P. , Comont, D. , Callaghan, T. M. , Bussell, J. , Brand, G. , Gwynn‐Jones, D. , *et al* (2018) Vegetation and edaphic factors influence rapid establishment of distinct fungal communities on former coal‐spoil sites. Fungal Ecol 33: 92–103.

[emi14327-bib-0021] Dijkstra, P. , Ishizu, A. , Doucett, R. , Hart, S. C. , Schwartz, E. , Menyailo, O. V. , and Hungate, B. A. (2006) ^13^C‐ and ^15^N natural abundance of the soil microbial biomass. Soil Biol Biochem 38: 3257–3266.

[emi14327-bib-0022] Emmerton, K. S. , Callaghan, T. V. , Jones, H. E. , Leake, J. R. , Michelsen, A. , and Read, D. J. (2001) Assimilation and isotopic fractionation of nitrogen by mycorrhizal and nonmycorrhizal subarctic plants. New Phytol 151: 513–524.

[emi14327-bib-0023] Evans, R. D. (2007) Soil nitrogen isotope composition . In Stable isotopes in Ecology and Environmental Science, pp. 83–98. John Wiley & Sons, New York.

[emi14327-bib-0024] Fitter, A. , Gilligan, C. , Hollingworth, K. , Kleczkowski, A. , Twyman, R. , and Pitchford, J. (2005) Biodiversity and ecosystem function in soil. Funct Ecol 19: 369–377.

[emi14327-bib-0025] Free, S. J. (2013) Fungal cell wall organization and biosynthesis. Adv Genet 81: 33–82. 23419716 10.1016/B978-0-12-407677-8.00002-6

[emi14327-bib-0026] Gebauer, G. , and Taylor, A. F. S. (1999) ^15^N natural abundance in fruit bodies of different functional groups of fungi in relation to substrate utilization. New Phytol 142: 93–101.

[emi14327-bib-0027] Gebauer, G. , and Meyer, M. (2003) ^15^N and ^13^C natural abundance of autotrophic and myco‐heterotrophic orchids provides insight into nitrogen and carbon gain from fungal association. New Phytol 160: 209–223. 10.1046/j.1469-8137.2003.00872.x 33873535

[emi14327-bib-0028] Geml, J. , Gravendeel, B. , van der Gaag, K. J. , Neilen, M. , Lammers, Y. , Raes, N. , *et al* (2014) The contribution of DNA metabarcoding to fungal conservation: diversity assessment, habitat partitioning and mapping red‐listed fungi in protected coastal *Salix repens* communities in The Netherlands. PLoS One 9: e99852. 24937200 10.1371/journal.pone.0099852 PMC4061046

[emi14327-bib-0029] Gleixner, G. , Poirier, N. , Bol, R. , and Balesdent, J. (2002) Molecular dynamics of organic matter in a cultivated soil. Org Geochem 33: 357–366.

[emi14327-bib-0030] Griffith, G. W. (2004) The use of stable isotopes in fungal ecology. Mycologist 18: 177–183.

[emi14327-bib-0031] Griffith, G. W. , and Roderick, K. (2008)Saprotrophic basidiomycetes in grasslands: distribution and function . In Ecology of Saprotrophic Basidiomycetes British Mycological Society Symposia Series, Boddy L., Frankland J. C., and van West P. (eds). London: Elsevier Ltd, pp. 277–299.

[emi14327-bib-0032] Griffith, G. W. , Easton, G. L. , and Jones, A. W. (2002) Ecology and diversity of waxcap (*Hygrocybe* spp.) fungi. Bot J Scotl 54: 7–22.

[emi14327-bib-0033] Griffith, G. W. , Bratton, J. L. , and Easton, G. L. (2004) Charismatic megafungi: the conservation of waxcap grasslands. Br Wildl 15: 31–43.

[emi14327-bib-0034] Griffith, G. W. , Graham, A. , Woods, R. G. , Easton, G. L. , and Halbwachs, H. (2014) Effect of biocides on the fruiting of waxcap fungi. Fungal Ecol 7: 67–69.

[emi14327-bib-0035] Griffith, G. W. , Gamarra, J. P. , Holden, E. M. , Mitchel, D. , Graham, A. , Evans, D. A. , *et al* (2013) The international conservation importance of welsh 'waxcap' grasslands. Mycosphere 4: 969–984.

[emi14327-bib-0037] Halbwachs, H. , Karasch, P. , and Griffith, G. W. (2013a) The diverse habitats of *Hygrocybe* – peeking into an enigmatic lifestyle. Mycosphere 4: 773–792.

[emi14327-bib-0038] Halbwachs, H. , Dentinger, B. T. , Detheridge, A. P. , Karasch, P. , and Griffith, G. W. (2013b) Hyphae of waxcap fungi colonise plant roots. Fungal Ecol 6: 487–492.

[emi14327-bib-0039] Handley, L. L. , and Raven, J. A. (1992) The use of natural abundance of nitrogen isotopes in plant physiology and ecology. Plant Cell Environ 15: 965–985.

[emi14327-bib-0040] Harrington, T. J. , and Mitchell, D. T. (2002) Colonization of root systems of *Carex flacca* and *C. pilulifera* by *Cortinarius* (*Dermocybe*) *cinnamomeus* . Mycol Res 106: 452–459.

[emi14327-bib-0041] Heijden, M. G. , Martin, F. M. , Selosse, M. A. , and Sanders, I. R. (2015) Mycorrhizal ecology and evolution: the past, the present, and the future. New Phytol 205: 1406–1423. 25639293 10.1111/nph.13288

[emi14327-bib-0042] Hesler, L. R. , and Smith, A. H. (1963) North American Species of Hygrophoraceae. Knoxville, Tennessee: University of Tennessee Press.

[emi14327-bib-0043] Hobbie, E. A. , and Ouimette, A. P. (2009) Controls of nitrogen isotope patterns in soil profiles. Biogeochemistry 95: 355–371.

[emi14327-bib-0044] Hobbie, E. A. , and Agerer, R. (2010) Nitrogen isotopes in ectomycorrhizal sporocarps correspond to belowground exploration types. Plant Soil 327: 71–83.

[emi14327-bib-0045] Hobbie, E. A. , and Högberg, P. (2012) Nitrogen isotopes link mycorrhizal fungi and plants to nitrogen dynamics. New Phytol 196: 367–382. 22963677 10.1111/j.1469-8137.2012.04300.x

[emi14327-bib-0046] Hobbie, E. A. , Sánchez, F. S. , and Rygiewicz, P. T. (2012) Controls of isotopic patterns in saprotrophic and ectomycorrhizal fungi. Soil Biol Biochem 48: 60–68.

[emi14327-bib-0047] Hobbie, E. A. , Weber, N. S. , Trappe, J. M. , and van Klinken, G. J. (2002) Using radiocarbon to determine the mycorrhizal status of fungi. New Phytol 156: 129–136.

[emi14327-bib-0048] Hobbie, E. A. , Diepen, L. T. , Lilleskov, E. A. , Ouimette, A. P. , Finzi, A. C. , and Hofmockel, K. S. (2014) Fungal functioning in a pine forest: evidence from a ^15^N‐labeled global change experiment. New Phytol 201: 1431–1439. 24304469 10.1111/nph.12578

[emi14327-bib-0049] Högberg, P. , Hogbom, L. , Schinkel, H. , Högberg, M. , Johannisson, C. , and Wallmark, H. (1996) ^15^N abundance of surface soils, roots and mycorrhizas in profiles of European forest soils. Oecologia 108: 207–214. 28307831 10.1007/BF00334643

[emi14327-bib-0050] Johnson, D. , Leake, J. R. , Ostle, N. , Ineson, P. , and Read, D. J. (2002) *In situ* ^13^CO_2_ pulse‐labelling of upland grassland demonstrates a rapid pathway of carbon flux from arbuscular mycorrhizal mycelia to the soil. New Phytol 153: 327–334.

[emi14327-bib-0051] Jumpponen, A. , and Jones, K. L. (2014) Tallgrass prairie soil fungal communities are resilient to climate change. Fungal Ecol 10: 44–57.

[emi14327-bib-0052] Kariman, K. , Barker, S. J. , Jost, R. , Finnegan, P. M. , and Tibbett, M. (2014) A novel plant–fungus symbiosis benefits the host without forming mycorrhizal structures. New Phytol 201: 1413–1422. 24279681 10.1111/nph.12600

[emi14327-bib-0053] Keizer, P. J. (1993)The influence of nature management on the macromycete fungi . In Fungi in Europe: Investigations, Recording and Conservation, Pegler D. N., Boddy L., Ing B., and Kirk P. M. (eds). Kew: Royal Botanic Gardens, pp. 251–269.

[emi14327-bib-0054] Klironomos, J. N. , and Hart, M. M. (2001) Food‐web dynamics: animal nitrogen swap for plant carbon. Nature 410: 651–652. 10.1038/35070643 11287942

[emi14327-bib-0055] Kohzu, A. , Yoshioka, T. , Ando, T. , Takahashi, M. , Koba, K. , and Wada, E. (1999) Natural ^13^C and ^15^N abundance of field‐collected fungi and their ecological implications. New Phytol 144: 323–330.

[emi14327-bib-0056] Kuan, H. , Fenwick, C. , Glover, L. A. , Griffiths, B. , and Ritz, K. (2006) Functional resilience of microbial communities from perturbed upland grassland soils to further persistent or transient stresses. Soil Biol Biochem 38: 2300–2306.

[emi14327-bib-0057] Levin, I. , Hammer, S. , Kromer, B. , and Meinhardt, F. (2008) Radiocarbon observations in atmospheric CO2: determining fossil fuel CO_2_ over Europe using Jungfraujoch observations as background. Sci Total Environ 391: 211–216. 18037473 10.1016/j.scitotenv.2007.10.019

[emi14327-bib-0058] Lilleskov, E. A. , Fahey, T. J. , and Lovett, G. M. (2001) Ectomycorrhizal fungal aboveground community change over an atmospheric nitrogen deposition gradient. Ecol Appl 11: 397–410.

[emi14327-bib-0059] Lilleskov, E. A. , Hobbie, E. A. , and Fahey, T. J. (2002) Ectomycorrhizal fungal taxa differing in response to nitrogen deposition also differ in pure culture organic nitrogen use and natural abundance of nitrogen isotopes. New Phytol 154: 219–231.

[emi14327-bib-0060] Lodge, D. J. , and Cantrell, S. (1995) Diversity of litter agarics at Cuyabeno, Ecuador: calibrating sampling efforts in tropical rainforest. Mycologist 9: 149–151.

[emi14327-bib-0061] Lodge, D. J. , Padamsee, M. , Matheny, P. B. , Aime, M. C. , Cantrell, S. A. , Boertmann, D. , *et al* (2014) Molecular phylogeny, morphology, pigment chemistry and ecology in Hygrophoraceae (Agaricales). Fungal Divers 64: 1–99.

[emi14327-bib-0062] Merckx, V. , Stöckel, M. , Fleischmann, A. , Bruns, T. D. , and Gebauer, G. (2010) ^15^N and ^13^C natural abundance of two mycoheterotrophic and a putative partially mycoheterotrophic species associated with arbuscular mycorrhizal fungi. New Phytol 188: 590–596. 20618915 10.1111/j.1469-8137.2010.03365.x

[emi14327-bib-0063] Merzendorfer, H. (2011) The cellular basis of chitin synthesis in fungi and insects: common principles and differences. Eur J Cell Biol 90: 759–769. 21700357 10.1016/j.ejcb.2011.04.014

[emi14327-bib-0064] Murray, P. J. , Clegg, C. D. , Crotty, F. V. , de la Fuente Martinez, N. , Williams, J. K. , and Blackshaw, R. P. (2009) Dissipation of bacterially derived C and N through the meso‐and macrofauna of a grassland soil. Soil Biol Biochem 41: 1146–1150.

[emi14327-bib-0065] Murray, P. J. , Cook, R. , Currie, A. F. , Dawson, L. A. , Gange, A. C. , Grayston, S. J. , and Treonis, A. M. (2006) Interactions between fertilizer addition, plants and the soil environment: implications for soil faunal structure and diversity. Appl Soil Ecol 33: 199–207.

[emi14327-bib-0066] Neilson, R. , Boag, B. , and Smith, M. (2000) Earthworm δ^13^C and δ^15^N analyses suggest that putative functional classifications of earthworms are site‐specific and may also indicate habitat diversity. Soil Biol Biochem 32: 1053–1061.

[emi14327-bib-0036] Nguyen, N. H. , Song, Z. , Bates, S. T. , Branco, S. , Tedersoo, L. , Menke, J. , *et al* (2015) FUNGuild: an open annotation tool for parsing fungal community datasets by ecological guild. Fungal Ecol. 20: 214–248.

[emi14327-bib-0067] Pelosi, C.l. , Barot, S.b. , Capowiez, Y. , Hedde, M.l. , and Vandenbulcke, F. (2014) Pesticides and earthworms. A review. Agronomy Sust Dev 34: 199–228.

[emi14327-bib-0068] Preiss, K. , and Gebauer, G. (2008) A methodological approach to improve estimates of nutrient gains by partially myco‐heterotrophic plants. Isot Environ Health Stud 44: 393–401. 10.1080/10256010802507458 19061069

[emi14327-bib-0069] Ram, A. F. J. , Arentshorst, M. , Damveld, R. A. , Klis, F. M. , and van den Hondel, C. A. (2004) The cell wall stress response in *Aspergillus Niger* involves increased expression of the glutamine: fructose‐6‐phosphate amidotransferase‐encoding gene (*gfaA*) and increased deposition of chitin in the cell wall. Microbiology 150: 3315–3326. 15470111 10.1099/mic.0.27249-0

[emi14327-bib-0070] Rambold, G. , and Agerer, R. (1997) DEEMY: the concept of a characterization and determination system for ectomycorrhizae. Mycorrhiza 7: 113–116.

[emi14327-bib-0071] Reimer, P. J. , Brown, T. A. , and Reimer, R. W. (2004) Discussion: reporting and calibration of post‐bomb ^14^C data. Radiocarbon 46: 1299–1304.

[emi14327-bib-0072] Roderick, K. (2009) The ecology of grassland macrofungi. PhD thesis. IBERS: Aberystwyth University.

[emi14327-bib-0073] Schiebold, J. M.‐I. , Bidartondo, M. I. , Karasch, P. , Gravendeel, B. , and Gebauer, G. (2017) You are what you get from your fungi: nitrogen stable isotope patterns in Epipactis species. Ann Bot 119: 1085–1095. 28334113 10.1093/aob/mcw265 PMC5604585

[emi14327-bib-0074] Schmidt, O. , Curry, J. P. , Dyckmans, J. , Rota, E. , and Scrimgeour, C. M. (2004) Dual stable isotope analysis (δ^13^C and δ^15^N) of soil invertebrates and their food sources. Pedobiologia 48: 171–180.

[emi14327-bib-0075] Seitzman, B. H. , Ouimette, A. , Mixon, R. L. , Hobbie, E. A. , and Hibbett, D. S. (2011) Conservation of biotrophy in *Hygrophoraceae* inferred from combined stable isotope and phylogenetic analyses. Mycologia 103: 280–290. 21139028 10.3852/10-195

[emi14327-bib-0076] Selosse, M.‐A. , and Martos, F. (2014) Do chlorophyllous orchids heterotrophically use mycorrhizal fungal carbon? Trends Plant Sci 19: 683–685. 25278267 10.1016/j.tplants.2014.09.005

[emi14327-bib-0077] Silvertown, J. , Poulton, P. R. , Johnston, E. , Edwards, G. , Heard, M. , and Biss, P. M. (2006) The park grass experiment 1856–2006: its contribution to ecology. J Ecol 94: 801–814.

[emi14327-bib-0078] Slota, P. , Jull, A. J. T. , Linick, T. , and Toolin, L. J. (1987) Preparation of small samples for ^14^C accelerator targets by catalytic reduction of CO. Radiocarbon 29: 303–306.

[emi14327-bib-0079] Staddon, P. L. , Ramsey, C. B. , Ostle, N. , Ineson, P. , and Fitter, A. H. (2003) Rapid turnover of hyphae of mycorrhizal fungi determined by AMS microanalysis of C‐14. Science 300: 1138–1140. 12750519 10.1126/science.1084269

[emi14327-bib-0080] Stuiver, M. , and Polach, H. A. (1977) Reporting of ^14^C data. Radiocarbon 19: 355–363.

[emi14327-bib-0081] Taylor, A. F. S. , Fransson, P. M. , Högberg, P. , Högberg, M. N. , and Plamboeck, A. H. (2003) Species level patterns in ^13^C and ^15^N abundance of ectomycorrhizal and saprotrophic fungal sporocarps. New Phytol 159: 757–774. 10.1046/j.1469-8137.2003.00838.x 33873595

[emi14327-bib-0082] Taylor, A. F. S. , Högbom, L. , Högberg, M. , Lyon, A. J. E. , Nasholm, T. , and Högberg, P. (1997) Natural ^15^N abundance in fruit bodies of ectomycorrhizal fungi from boreal forests. New Phytol 136: 713–720. 10.1046/j.1469-8137.1997.00788.x 33863102

[emi14327-bib-0083] Tedersoo, L. , and Nara, K. (2010) General latitudinal gradient of biodiversity is reversed in ectomycorrhizal fungi. New Phytol 185: 351–354. 20088976 10.1111/j.1469-8137.2009.03134.x

[emi14327-bib-0084] Tedersoo, L. , Naadel, T. , Bahram, M. , Pritsch, K. , Buegger, F. , Leal, M. , *et al* (2012) Enzymatic activities and stable isotope patterns of ectomycorrhizal fungi in relation to phylogeny and exploration types in an afrotropical rain forest. New Phytol 195: 832–843. 22758212 10.1111/j.1469-8137.2012.04217.x

[emi14327-bib-0085] Tedersoo, L. , Nilsson, R. H. , Abarenkov, K. , Jairus, T. , Sadam, A. , Saar, I. , *et al* (2010) 454 pyrosequencing and sanger sequencing of tropical mycorrhizal fungi provide similar results but reveal substantial methodological biases. New Phytol 188: 291–301. 20636324 10.1111/j.1469-8137.2010.03373.x

[emi14327-bib-0086] Tello, S. A. , Silva‐Flores, P. , Agerer, R. , Halbwachs, H. , Beck, A. , and Peršoh, D. (2014) *Hygrocybe virginea* is a systemic endophyte of *Plantago lanceolata* . Mycol Prog 13: 471–475.

[emi14327-bib-0087] Temperton, V. M. , Märtin, L. L. , Luecke, A. , Röder, D. , and Kiehl, K. (2012) Effects of four different restoration treatments on the natural abundance of ^15^N stable isotopes in plants. Front Plant Sci 3: 70. 22645597 10.3389/fpls.2012.00070 PMC3355755

[emi14327-bib-0088] Tiunov, A. V. (2007) Stable isotopes of carbon and nitrogen in soil ecological studies. Biol Bull 34: 395–407. 17966909

[emi14327-bib-0089] Toju, H. , Sato, H. , and Tanabe, A. S. (2014) Diversity and spatial structure of belowground plant–fungal symbiosis in a mixed subtropical forest of ectomycorrhizal and arbuscular mycorrhizal plants. PLoS One 9: e86566. 24489745 10.1371/journal.pone.0086566 PMC3904951

[emi14327-bib-0090] Treonis, A. M. , Ostle, N. J. , Stott, A. W. , Primrose, R. , Grayston, S. J. , and Ineson, P. (2004) Identification of groups of metabolically‐active rhizopshere microorganisms by stable isotope probing of PLFAs. Soil Biol Biochem 36: 533–537.

[emi14327-bib-0091] Treseder, K. K. , Torn, M. S. , and Masiello, C. A. (2006) An ecosystem‐scale radiocarbon tracer to test use of litter carbon by ectomycorrhizal fungi. Soil Biol Biochem 38: 1077–1082.

[emi14327-bib-0092] Trudell, S. A. , Rygiewicz, P. T. , and Edmonds, R. L. (2004) Patterns of nitrogen and carbon stable isotope ratios in macrofungi, plants and soils in two old growth conifer forests. New Phytol 164: 317–335. 10.1111/j.1469-8137.2004.01162.x 33873563

[emi14327-bib-0093] Veldre, V. , Abarenkov, K. , Bahram, M. , Martos, F. , Selosse, M.‐A. , Tamm, H. , *et al* (2013) Evolution of nutritional modes of Ceratobasidiaceae (Cantharellales, Basidiomycota) as revealed from publicly available ITS sequences. Fungal Ecol 6: 256–268.

[emi14327-bib-0094] Vera, F. W. M. (2000) Grazing Ecology and Forest History. New York: CABI Publishing.

[emi14327-bib-0095] Wang, B. , and Qiu, Y. L. (2006) Phylogenetic distribution and evolution of mycorrhizas in land plants. Mycorrhiza 16: 299–363. 16845554 10.1007/s00572-005-0033-6

[emi14327-bib-0096] Weiss, M. , Waller, F. , Zuccaro, A. , and Selosse, M. (2016) Sebacinales‐one thousand and one interactions with land plants. New Phytol 211: 20–40. 27193559 10.1111/nph.13977

[emi14327-bib-0097] Wingler, A. , Wallenda, T. , and Hampp, R. (1996) Mycorrhiza formation on Norway spruce (*Picea abies*) roots affects the pathway of anaplerotic CO_2_ fixation. Physiol Plant 96: 699–705.

[emi14327-bib-0098] Wilson, D. (1995) Endophyte: the evolution of a term, and clarification of its use and definition. Oikos 73: 274–276.

[emi14327-bib-0099] Zimmer, K. , Hynson, N. A. , Gebauer, G. , Allen, E. B. , Allen, M. F. , and Read, D. J. (2007) Wide geographical and ecological distribution of nitrogen and carbon gains from fungi in pyroloids and monotropoids (Ericaceae) and in orchids. New Phytol 175: 166–175. 17547676 10.1111/j.1469-8137.2007.02065.x

