# Supplementary material for: Isotopic evidence of biotrophy and unusual nitrogen nutrition in soil‐dwelling Hygrophoraceae
Source: Environ Microbiol. 2018 Oct 17;20(10):3573–88. doi: 10.1111/1462-2920.14327 (PMC6849620; doi:10.1111/1462-2920.14327)
Supplement: Supplementary file 1 — Suppdata 1. Details of Sourhope surveys Suppdata 2. δ13C and δ15N values of soils and vegetation at Amorbach and Sourhope. Suppdata 3. Sampling locations for basidiocarp isotope analyses Suppdata 4. δ15N / δ13C in basidiocarps of different ages between cap vs. stipe tissues. Suppdata 5. Images of C. lacmus and H. cantharellus growing in heather/moss Suppdata 6. Spatial distribution of C. pratensis and C. virgineus basidiocarps at Sourhope Suppdata 7. Data from the seven waxcap Basidiocarps close to pulse sites Suppdata 8. Summary of Hobbie and Agerer theoretical pattern of N isotope fractionation in mycorrhizal systems Suppdata 9. δ15N values in soil invertebrates from published studies Suppdata 10. Raw data and references for published studies relating to Fig. 3 [file EMI-20-3573-s001.pdf]

Suppdata 1: Details of Sourhope surveys

TREATMENT	PLOT NO.	*C. lacmus*	*C. pratensis*	*C. virgineus*	*G. irrigatus*	*G. laetus*	*G. psittacinus*	*H. ceracea*	*H. conica*	*H. insipida*	*H. quieta*	*H. reidii*	*H. splendid-issima*
**Control 1**	**1F**	0	42	0	0	26	1	0	1	0	0	4	0
**Control 1**	**2B**	0	698	0	0	42	4	155	8	0	0	16	0
**Control 1**	**3D**	0	145	0	0	0	1	17	8	0	0	0	0
**Control 1**	**4D**	0	65	0	0	36	0	0	0	0	0	0	0
**Control 1**	**5A**	0	55	3	0	51	0	14	5	0	2	22	0
**Control 2**	**1D**	1	38	0	1	37	3	0	0	0	0	1	0
**Control 2**	**2F**	0	403	0	0	221	4	99	2	0	0	7	0
**Control 2**	**3E**	0	384	0	0	2	7	0	1	0	0	0	0
**Control 2**	**4A**	0	58	0	0	130	0	21	9	1	0	52	0
**Control 2**	**5F**	0	153	6	0	38	0	0	19	0	0	0	0
**Biocide**	**1E**	0	4	0	0	0	0	0	4	0	0	0	0
**Biocide**	**2D**	0	11	0	0	1	10	19	7	0	0	1	0
**Biocide**	**3C**	0	2	0	0	4	1	0	2	0	0	10	0
**Biocide**	**4B**	0	16	0	0	3	0	2	0	0	0	0	0
**Biocide**	**5E**	0	84	0	0	2	0	5	7	0	1	0	0
**Lime**	**1B**	0	2	0	0	0	0	0	0	0	0	2	0
**Lime**	**2C**	0	1	0	0	0	0	0	3	0	0	0	0
**Lime**	**3B**	0	2	3	0	0	0	0	4	0	0	0	0
**Lime**	**4F**	0	0	0	0	0	0	0	0	0	0	0	0
**Lime**	**5B**	0	5	1	0	0	0	0	11	0	0	0	0
**Nitrogen**	**1C**	3	111	0	0	43	6	110	0	0	0	0	0
**Nitrogen**	**2A**	0	17	0	0	11	0	16	1	0	0	0	3
**Nitrogen**	**3F**	0	84	0	0	0	5	0	0	0	0	0	0
**Nitrogen**	**4E**	0	4	0	0	1	0	0	0	0	0	0	0
**Nitrogen**	**5C**	0	24	7	0	15	0	1	2	0	0	0	0
**Nitrogen & Lime**	**1A**	0	0	0	0	0	0	2	3	0	0	0	0
**Nitrogen & Lime**	**2E**	0	0	2	0	0	0	0	0	0	0	0	0
**Nitrogen & Lime**	**3A**	0	0	3	0	0	0	0	3	0	0	0	0
**Nitrogen & Lime**	**4C**	0	0	0	0	0	0	0	0	0	0	0	0
**Nitrogen & Lime**	**5D**	0	0	3	0	0	0	0	0	0	0	0	0
	**TOTAL**	4	2408	28	1	663	42	461	100	1	3	115	3

Species	13-Sep-01	1-Oct-01	18-Oct-01	30-Oct-01	15-Nov-01	29-Aug-02	12-Sep-02	26-Sep-02	11-Oct-02	25-Oct-02	7-Nov-02	25-Sep-03	10-Oct-03	5-Nov-03	30-Sep-04	21-Oct-04	27-Sep-05	21-Oct-05	No. visits (/18) where species was found
Cuphophyllus virgineus	1	1	1	0	0	0	0	0	0	1	1	0	0	0	1	1	1	1	**7**
Cuphophyllus lacmus	0	0	0	0	0	0	0	0	0	0	0	0	0	0	1	1	0	1	**2**
Cuphophyllus pratensis	1	1	1	1	0	1	1	1	1	1	1	1	1	1	1	1	1	1	**17**
Gliophorus irrigata	0	0	0	0	0	0	1	0	1	0	0	0	0	0	1	1	0	0	**1**
Gliophorus psittacina	1	1	0	0	0	0	0	1	1	0	0	0	0	0	1	1	1	1	**7**
Hygrocybe ceracea	0	1	1	1	0	0	1	1	1	0	0	0	0	0	1	1	1	1	**11**
Hygrocybe conica	0	1	1	1	0	0	1	1	1	0	0	0	0	0	1	1	1	1	**10**
Hygrocybe insipida	0	0	0	0	0	0	0	0	0	0	0	0	0	0	0	0	0	1	**1**
Hygrocybe laeta	1	1	1	1	0	0	1	1	1	1	0	0	0	0	1	1	1	1	**12**
Hygrocybe quieta	0	0	0	0	0	0	0	0	0	0	0	0	0	0	1	0	0	1	**2**
Hygrocybe reidii	1	1	0	1	0	1	1	1	1	0	0	0	1	0	1	1	1	1	**11**
Hygrocybe splendidissima	0	0	1	0	0	0	0	0	0	1	0	0	0	0	1	1	1	1	**3**
No. species Hygrophoraceae	**5**	**7**	**6**	**5**	**0**	**2**	**6**	**6**	**7**	**4**	**2**	**1**	**2**	**1**	**11**	**10**	**8**	**11**	

Details of vegetation analyses and site management regimes at the Sourhope site are given in Burt-Smith, G. (2001; Report III. Results from the Sourhope Field Experiment: 1999-2001. unpublished; 24pp; available from http://soilbionerc.ac.uk/Download/Report - 3 - allpdf) and Burt-Smith, G. (2003; NERC Soil Biodiversity Thematic Programme. Report III. Results from the Sourhope field experiment 1999-2003. http://users.aber.ac.uk/gwg/pdf/NERC Soil Biodiversity Programme 1998-2004 datasets.zip) respectively.

Suppdata 2. $\delta^{13}C$ and $\delta^{15}N$ values of associated (A) soil and (B)plants tissues from waxcap grasslands in UK, Germany and Iceland.

A) SOIL	Soil Depth	Replicate	Country	Site	Lat/Long	Coll Date	δ15N (‰)	δ13C (‰)	Total N (%)	Total C (%)	C/N ratio
SOIL	0-2.5cm	Core A	Scotland	Sourhope	55.4700, -2.2312	1-Jun-02	2.80	-27.00	nd	nd	nd
SOIL	0-2.5cm	Core B	Scotland	Sourhope	55.4700, -2.2312	1-Jun-02	2.79	-26.80	nd	nd	nd
SOIL	0-2.5cm	Core C	Scotland	Sourhope	55.4700, -2.2312	1-Jun-02	3.10	-27.80	nd	nd	nd
SOIL	2.5-5.0cm	Core A	Scotland	Sourhope	55.4700, -2.2312	1-Jun-02	4.30	-27.20	nd	nd	nd
SOIL	2.5-5.0cm	Core B	Scotland	Sourhope	55.4700, -2.2312	1-Jun-02	2.70	-26.40	nd	nd	nd
SOIL	2.5-5.0cm	Core C	Scotland	Sourhope	55.4700, -2.2312	1-Jun-02	3.80	-27.30	nd	nd	nd
SOIL	5.0-7.5cm	Core A	Scotland	Sourhope	55.4700, -2.2312	1-Jun-02	4.02	-26.80	nd	nd	nd
SOIL	5.0-7.5cm	Core B	Scotland	Sourhope	55.4700, -2.2312	1-Jun-02	4.89	-26.20	nd	nd	nd
SOIL	5.0-7.5cm	Core C	Scotland	Sourhope	55.4700, -2.2312	1-Jun-02	3.70	-26.50	nd	nd	nd
SOIL	7.5-10.0cm	Core A	Scotland	Sourhope	55.4700, -2.2312	1-Jun-02	5.40	-26.10	nd	nd	nd
SOIL	7.5-10.0cm	Core B	Scotland	Sourhope	55.4700, -2.2312	1-Jun-02	6.12	-26.00	nd	nd	nd
SOIL	7.5-10.0cm	Core C	Scotland	Sourhope	55.4700, -2.2312	1-Jun-02	5.80	-26.30	nd	nd	nd
SOIL	0-3 cm	Site 1_A0	Germany	Amorbach	49.6460, 9.2010	12-Apr-09	5.29	-29.47	0.498	5.63	11.32
SOIL	0-3 cm	Site 1_B0	Germany	Amorbach	49.6460, 9.2010	12-Apr-09	3.18	-29.52	0.361	4.17	11.55
SOIL	0-3 cm	Site 1_C0	Germany	Amorbach	49.6460, 9.2010	12-Apr-09	2.91	-29.26	0.349	3.83	10.96
SOIL	0-3 cm	Site 1_D0	Germany	Amorbach	49.6460, 9.2010	12-Apr-09	2.32	-29.79	0.463	5.55	12.01
SOIL	0-3 cm	Site 1_E0	Germany	Amorbach	49.6460, 9.2010	12-Apr-09	2.00	-29.51	0.495	5.71	11.53
SOIL	3-8 cm	Site 1_A1	Germany	Amorbach	49.6460, 9.2010	12-Apr-09	3.22	-29.03	0.385	4.06	10.55
SOIL	3-8 cm	Site 1_B1	Germany	Amorbach	49.6460, 9.2010	12-Apr-09	4.04	-28.90	0.330	3.37	10.22
SOIL	3-8 cm	Site 1_C1	Germany	Amorbach	49.6460, 9.2010	12-Apr-09	4.96	-28.36	0.212	2.01	9.50
SOIL	3-8 cm	Site 1_D1	Germany	Amorbach	49.6460, 9.2010	12-Apr-09	4.22	-28.48	0.253	2.46	9.72
SOIL	3-8 cm	Site 1_E1	Germany	Amorbach	49.6460, 9.2010	12-Apr-09	3.03	-28.80	0.364	4.06	11.16
SOIL	8-13 cm	Site 1_A2	Germany	Amorbach	49.6460, 9.2010	12-Apr-09	4.99	-28.22	0.253	2.47	9.75
SOIL	8-13 cm	Site 1_B2	Germany	Amorbach	49.6460, 9.2010	12-Apr-09	4.03	-28.84	0.244	2.50	10.25
SOIL	8-13 cm	Site 1_C2	Germany	Amorbach	49.6460, 9.2010	12-Apr-09	5.98	-28.39	0.216	2.00	9.25
SOIL	8-13 cm	Site 1_D2	Germany	Amorbach	49.6460, 9.2010	12-Apr-09	4.05	-28.54	0.246	2.38	9.68
SOIL	8-13 cm	Site 1_E2	Germany	Amorbach	49.6460, 9.2010	12-Apr-09	4.87	-28.26	0.170	1.56	9.16
SOIL	13-18 cm	Site 1_A3	Germany	Amorbach	49.6460, 9.2010	12-Apr-09	7.11	-26.87	0.116	1.11	9.62
SOIL	13-18 cm	Site 1_B3	Germany	Amorbach	49.6460, 9.2010	12-Apr-09	6.10	-27.34	0.138	1.27	9.22
SOIL	13-18 cm	Site 1_C3	Germany	Amorbach	49.6460, 9.2010	12-Apr-09	7.34	-27.08	0.130	1.20	9.22
SOIL	13-18 cm	Site 1_D3	Germany	Amorbach	49.6460, 9.2010	12-Apr-09	5.47	-27.77	0.155	1.42	9.16
SOIL	13-18 cm	Site 1_E3	Germany	Amorbach	49.6460, 9.2010	12-Apr-09	7.55	-26.95	0.126	1.03	8.16
SOIL	18-23 cm	Site 1_A4	Germany	Amorbach	49.6460, 9.2010	12-Apr-09	8.27	-26.73	0.066	0.60	9.12
SOIL	18-23 cm	Site 1_B4	Germany	Amorbach	49.6460, 9.2010	12-Apr-09	7.34	-26.30	0.072	0.69	9.54
SOIL	18-23 cm	Site 1_C4	Germany	Amorbach	49.6460, 9.2010	12-Apr-09	8.83	-26.26	0.056	0.51	9.13
SOIL	18-23 cm	Site 1_D4	Germany	Amorbach	49.6460, 9.2010	12-Apr-09	7.68	-26.40	0.066	0.60	9.05
SOIL	18-23 cm	Site 1_E4	Germany	Amorbach	49.6460, 9.2010	12-Apr-09	8.29	-26.74	0.067	0.62	9.24
SOIL	0-3 cm	1	Iceland	Dalvík	65.8912,-18.6200	1-Sep-16	1.01	-27.82	0.56	10.09	15.45
SOIL	0-3 cm	1	Iceland	Seyðisfjörður	65.2735,-14.0124	1-Sep-16	3.55	-27.68	0.28	5.87	17.83

B) VEGETATION	Replicate	Country	Site	Lat/Long	Coll Date	δ15N (‰)	δ13C (‰)	Total N (%)	Total C (%)	C/N ratio
Agrostis capillaris (leaf)	1	Scotland	Sourhope	55.4700, -2.2312	1-Jun-02	2.70	-27.80	nd	nd	nd
Agrostis capillaris (leaf)	2	Scotland	Sourhope	55.4700, -2.2312	1-Jun-02	3.10	-28.00	nd	nd	nd
Agrostis capillaris (leaf)	3	Scotland	Sourhope	55.4700, -2.2312	1-Jun-02	1.80	-27.70	nd	nd	nd
Agrostis capillaris (fine, living root)	1	Scotland	Sourhope	55.4700, -2.2312	1-Jun-02	3.02	-27.40	nd	nd	nd
Agrostis capillaris (fine, living root)	2	Scotland	Sourhope	55.4700, -2.2312	1-Jun-02	3.51	-27.20	nd	nd	nd
Agrostis capillaris (fine, living root)	3	Scotland	Sourhope	55.4700, -2.2312	1-Jun-02	3.47	-27.40	nd	nd	nd
Rhytiadiadelphus squarrosus	1	Scotland	Sourhope	55.4700, -2.2312	30-Oct-01	-2.27	-31.12	nd	nd	nd
Luzula campestris (leaf)	1	England	Lundy	51.1694,-4.6720	11-Nov-04	1.58	-29.98	2.47	43.99	17.81
Luzula campestris (leaf)	1	Wales	Aberystwyth	52.4194,-4.0683	6-Apr-05	1.94	-30.37	2.77	44.78	16.17
Centaurea nigra (leaf)	1	Germany	Amorbach	49.6460, 9.2010	12-Apr-09	-0.15	-29.44	2.43	41.95	17.25
Centaurea nigra (leaf)	2	Germany	Amorbach	49.6460, 9.2010	12-Apr-09	-0.28	-29.07	2.41	40.99	17.01
Centaurea nigra (root)	1	Germany	Amorbach	49.6460, 9.2010	12-Apr-09	1.25	-28.32	1.02	41.38	40.52
Centaurea nigra (root)	2	Germany	Amorbach	49.6460, 9.2010	12-Apr-09	2.08	-28.07	1.00	41.60	41.66
Plantago lanceolata (leaf)	1	Germany	Amorbach	49.6460, 9.2010	12-Apr-09	-1.01	-28.10	1.43	44.36	31.01
Plantago lanceolata (leaf)	2	Germany	Amorbach	49.6460, 9.2010	12-Apr-09	-1.23	-28.08	1.45	44.51	30.80
Plantago lanceolata (leaf)	3	Germany	Amorbach	49.6460, 9.2010	12-Apr-09	-1.12	-28.09	nd	nd	nd
Plantago lanceolata (root)	1	Germany	Amorbach	49.6460, 9.2010	12-Apr-09	-1.12	-28.68	0.88	39.27	44.71
Plantago lanceolata (root)	2	Germany	Amorbach	49.6460, 9.2010	12-Apr-09	-0.97	-28.55	0.85	39.08	45.75
Leontodon sp. (leaf)	1	Germany	Amorbach	49.6460, 9.2010	12-Apr-09	1.50	-31.64	1.75	41.89	23.91
Leontodon sp. (leaf)	2	Germany	Amorbach	49.6460, 9.2010	12-Apr-09	1.92	-31.76	1.82	42.34	23.25
Leontodon sp. (leaf)	3	Germany	Amorbach	49.6460, 9.2010	12-Apr-09	1.71	-31.70	nd	nd	nd
Leontodon sp. (root)	1	Germany	Amorbach	49.6460, 9.2010	12-Apr-09	-1.33	-29.42	0.68	44.17	65.36
Leontodon sp. (root)	2	Germany	Amorbach	49.6460, 9.2010	12-Apr-09	-1.30	-29.39	0.67	44.34	66.22
Rhytiadiadelphus squarrosus	1	Germany	Amorbach	49.6460, 9.2010	12-Apr-09	-2.34	-31.09	1.35	38.32	28.39
Rhytiadiadelphus squarrosus	2	Germany	Amorbach	49.6460, 9.2010	12-Apr-09	-2.20	-31.14	1.34	38.07	28.47
Hieracium sp. (leaf)	1	Iceland	Dalvík	65.8912,-18.6200	1-Sep-16	-4.29	-29.33	1.77	43.21	24.43
Alchemilla mollis (leaf)	1	Iceland	Seyðisfjörður	65.2735,-14.0124	12-Sep-16	-3.83	-28.18	0.62	29.55	47.37
Gentiana sp. (leaf)	1	Iceland	Seyðisfjörður	65.2735,-14.0124	12-Sep-16	-1.47	-30.70	1.71	36.29	21.18

Suppdata 3. Raw data for isotopic analyses

	Genus	Species	Country		Lat/Long	Coll Date	Cap δ15N (‰)	Cap d13C (‰)	Cap % total N	Cap % total C	Cap C/N ratio	Notes
	SOURHOPE											
1	Cuphophyllus	pratensis	Scotland	Sourhope	55.4700, -2.2312	26-Sep-02	16.04	P-29.2	6.21	44.22	7.12	13C-pulse enriched
2	Cuphophyllus	pratensis	Scotland	Sourhope	55.4700, -2.2312	11-Oct-02	16.28	P-28.7	5.86	42.93	7.33	13C-pulse enriched
3	Cuphophyllus	pratensis	Scotland	Sourhope	55.4700, -2.2312	11-Oct-02	16.77	P-28.96	6.45	35.11	5.44	13C-pulse enriched
4	Cuphophyllus	pratensis	Scotland	Sourhope	55.4700, -2.2312	11-Oct-02	16.54	P-28.77	7.46	41.24	5.53	13C-pulse enriched
5	Cuphophyllus	pratensis	Scotland	Sourhope	55.4700, -2.2312	30-Oct-01	16.09	-27.66	6.40	41.78	6.53	
6	Cuphophyllus	pratensis	Scotland	Sourhope	55.4700, -2.2312	30-Oct-01	14.23	-27.93	5.60	41.57	7.43	
7	Cuphophyllus	pratensis	Scotland	Sourhope	55.4700, -2.2312	26-Sep-02	14.28	P-92.79	5.56	38.63	6.95	13C-pulse enriched
8	Cuphophyllus	pratensis	Scotland	Sourhope	55.4700, -2.2312	25-Oct-02	14.63	P-28.26	4.50	41.26	9.17	13C-pulse enriched
9	Cuphophyllus	pratensis	Scotland	Sourhope	55.4700, -2.2312	11-Oct-02	19.81	P-29.18	7.18	43.43	6.05	13C-pulse enriched
10	Cuphophyllus	pratensis	Scotland	Sourhope	55.4700, -2.2312	30-Oct-01	16.17	-28.04	6.39	41.84	6.55	
11	Cuphophyllus	pratensis	Scotland	Sourhope	55.4700, -2.2312	30-Oct-01	17.52	-28.75	7.18	43.33	6.04	
12	Cuphophyllus	pratensis	Scotland	Sourhope	55.4700, -2.2312	30-Oct-01	15.58	-29.43	4.88	42.83	8.78	
13	Cuphophyllus	pratensis	Scotland	Sourhope	55.4700, -2.2312	30-Oct-01	11.72	-28.17	6.28	44.03	7.01	
14	Cuphophyllus	pratensis	Scotland	Sourhope	55.4700, -2.2312	28-Aug-02	14.14	-28.61	5.19	43.83	8.45	
15	Cuphophyllus	pratensis	Scotland	Sourhope	55.4700, -2.2312	02-Oct-01	16.58	-29.37	7.59	44.73	5.89	
16	Cuphophyllus	pratensis	Scotland	Sourhope	55.4700, -2.2312	02-Oct-01	14.44	-28.36	7.08	45.48	6.42	
17	Cuphophyllus	pratensis	Scotland	Sourhope	55.4700, -2.2312	02-Oct-01	18.08	-29.37	6.40	45.60	7.13	
18	Cuphophyllus	pratensis	Scotland	Sourhope	55.4700, -2.2312	18-Oct-01	F14.89	-27.62	7.12	42.02	5.90	**Fertiliser plot**
19	Cuphophyllus	pratensis	Scotland	Sourhope	55.4700, -2.2312	18-Oct-01	F15.61	-28.38	6.40	45.64	7.13	**Fertiliser plot**
20	Cuphophyllus	pratensis	Scotland	Sourhope	55.4700, -2.2312	18-Oct-01	F15.73	-28.63	5.19	43.91	8.47	**Fertiliser plot**
21	Cuphophyllus	pratensis	Scotland	Sourhope	55.4700, -2.2312	13-Sep-01	F16.43	-29.21	6.69	44.19	6.60	**Fertiliser plot**
22	Cuphophyllus	pratensis	Scotland	Sourhope	55.4700, -2.2312	01-Oct-01	12.15	-29.10	6.28	43.08	6.86	
23	Cuphophyllus	pratensis	Scotland	Sourhope	55.4700, -2.2312	12-Sep-02	18.23	-29.29	7.29	43.13	5.92	
24	Cuphophyllus	pratensis	Scotland	Sourhope	55.4700, -2.2312	30-Oct-01	14.78	-28.54	5.05	42.95	8.50	
25	Cuphophyllus	pratensis	Scotland	Sourhope	55.4700, -2.2312	30-Oct-01	15.08	-28.24	5.60	43.31	7.73	
26	Cuphophyllus	pratensis	Scotland	Sourhope	55.4700, -2.2312	30-Oct-01	15.22	-28.13	5.92	43.89	7.41	
27	Cuphophyllus	pratensis	Scotland	Sourhope	55.4700, -2.2312	30-Oct-01	15.73	-28.55	6.85	41.66	6.08	
28	Cuphophyllus	pratensis	Scotland	Sourhope	55.4700, -2.2312	30-Oct-01	20.56	-29.24	6.83	42.85	6.27	
29	Cuphophyllus	pratensis	Scotland	Sourhope	55.4700, -2.2312	30-Oct-01	16.50	-29.19	6.20	43.95	7.09	
30	Cuphophyllus	pratensis	Scotland	Sourhope	55.4700, -2.2312	30-Oct-01	12.82	-28.80	6.64	41.37	6.23	
31	Cuphophyllus	pratensis	Scotland	Sourhope	55.4700, -2.2312	30-Oct-01	13.23	-28.84	6.41	42.91	6.69	
32	Cuphophyllus	pratensis	Scotland	Sourhope	55.4700, -2.2312	30-Oct-01	13.97	-27.81	7.01	42.59	6.08	
33	Cuphophyllus	pratensis	Scotland	Sourhope	55.4700, -2.2312	30-Oct-01	14.38	-28.50	5.98	41.16	6.89	
34	Cuphophyllus	virgineus	Scotland	Sourhope	55.4700, -2.2312	18-Oct-01	F17.07	-26.86	6.16	42.11	6.83	**Fertiliser plot**
35	Cuphophyllus	virgineus	Scotland	Sourhope	55.4700, -2.2312	01-Oct-01	F16.53	-27.99	6.08	43.76	7.20	**Fertiliser plot**
36	Cuphophyllus	virgineus	Scotland	Sourhope	55.4700, -2.2312	18-Oct-01	16.32	-27.66	5.44	45.77	8.41	
37	Gliophorus	laetus	Scotland	Sourhope	55.4700, -2.2312	02-Oct-01	16.78	-27.59	5.49	42.02	7.65	
38	Gliophorus	laetus	Scotland	Sourhope	55.4700, -2.2312	02-Oct-01	14.77	-28.56	5.85	48.52	8.29	
39	Gliophorus	laetus	Scotland	Sourhope	55.4700, -2.2312	01-Oct-01	F13.11	-27.89	5.39	43.30	8.04	**Fertiliser plot**
40	Gliophorus	laetus	Scotland	Sourhope	55.4700, -2.2312	18-Oct-01	F14.99	-28.17	4.17	41.70	10.00	**Fertiliser plot**
41	Gliophorus	laetus	Scotland	Sourhope	55.4700, -2.2312	02-Oct-01	14.12	-28.96	4.95	48.61	9.82	
42	Gliophorus	psittacinus	Scotland	Sourhope	55.4700, -2.2312	26-Sep-02	16.57	-27.61	5.62	44.40	7.90	
43	Gliophorus	psittacinus	Scotland	Sourhope	55.4700, -2.2312	13-Sep-01	18.99	-27.21	5.75	41.89	7.29	
44	Gliophorus	psittacinus	Scotland	Sourhope	55.4700, -2.2312	30-Oct-01	15.45	-27.72	6.07	41.68	6.87	
45	Hygrocybe	ceracea	Scotland	Sourhope	55.4700, -2.2312	11-Oct-02	11.58	nd	5.81	nd	nd	
46	Hygrocybe	ceracea	Scotland	Sourhope	55.4700, -2.2312	27-Sep-05	13.06	-27.74	7.19	42.81	5.95	
47	Hygrocybe	ceracea	Scotland	Sourhope	55.4700, -2.2312	01-Oct-01	F11.48	-28.48	5.45	43.60	8.00	**Fertiliser plot**
48	Hygrocybe	conica	Scotland	Sourhope	55.4700, -2.2312	02-Oct-01	14.23	-28.85	5.96	45.86	7.69	
49	Hygrocybe	conica	Scotland	Sourhope	55.4700, -2.2312	02-Oct-01	11.57	-29.28	5.45	49.19	9.03	
50	Hygrocybe	conica	Scotland	Sourhope	55.4700, -2.2312	02-Oct-01	12.49	-30.09	5.41	47.66	8.81	
51	Hygrocybe	splendidissima	Scotland	Sourhope	55.4700, -2.2312	02-Oct-01	12.09	-28.42	6.05	48.66	8.04	
						Mean	15.23	-28.44	6.08	43.44	7.27	
						SD	2.16	0.69	0.78	2.48	1.10	
						COUNT	42	43	51	50	50	
						Min	11.57	-30.09	4.17	35.11	5.44	
						Max	20.56	-26.86	7.59	49.19	10.00	
	NORTHERN EUROPE A (WALES) UK											
52	Cuphophyllus	lacmus	Wales	Maes Meddygon, Rhiwlas	53.1610,-4.1223	09-Nov-10	13.70	-29.62	3.29	39.13	11.90	
53	Cuphophyllus	pratensis	Wales	Bronydd Mawr	51.9840,-3.6318	31-Oct-02	13.54	-29.51	6.16	42.70	6.93	
54	Cuphophyllus	pratensis	Wales	Bronydd Mawr	51.9840,-3.6318	31-Oct-02	15.53	-29.48	5.42	42.82	7.90	
55	Cuphophyllus	pratensis	Wales	Bronydd Mawr	51.9840,-3.6318	31-Oct-02	15.71	-30.24	5.95	44.08	7.41	
56	Cuphophyllus	pratensis	Wales	Waunfawr	52.4149,-4.0476	02-Nov-01	14.14	-27.95	6.10	44.45	7.29	
57	Cuphophyllus	pratensis	Wales	Waunfawr	52.4149,-4.0476	02-Nov-01	15.16	-28.23	7.84	45.26	5.77	
58	Cuphophyllus	pratensis	Wales	Waunfawr	52.4149,-4.0476	02-Nov-01	15.33	-28.55	6.21	41.61	6.70	
59	Cuphophyllus	pratensis	Wales	Waunfawr	52.4149,-4.0476	02-Nov-01	15.46	-28.91	5.25	42.89	8.17	
60	Cuphophyllus	pratensis	Wales	Waunfawr	52.4149,-4.0476	02-Nov-01	16.37	-28.16	7.33	44.49	6.07	
61	Cuphophyllus	pratensis	Wales	Ystumtuen	52.4021,-3.8672	06-Nov-02	16.04	-29.59	5.25	43.97	8.38	
62	Cuphophyllus	pratensis	Wales	Ystumtuen	52.4021,-3.8672	06-Nov-02	16.91	-29.52	5.12	43.25	8.45	
63	Cuphophyllus	pratensis	Wales	Ystumtuen	52.4021,-3.8672	06-Nov-02	17.40	-29.30	7.05	42.39	6.01	
64	Cuphophyllus	virgineus	Wales	Bronydd Mawr	51.9840,-3.6318	16-Oct-03	14.44	-30.02	4.50	45.65	10.14	
65	Cuphophyllus	virgineus	Wales	Bronydd Mawr	51.9840,-3.6318	16-Oct-03	14.51	-30.40	5.97	43.80	7.34	
66	Cuphophyllus	virgineus	Wales	Bronydd Mawr	51.9840,-3.6318	16-Oct-04	15.39	-29.81	5.81	46.51	8.01	
67	Cuphophyllus	virgineus	Wales	Bronydd Mawr	51.9840,-3.6318	16-Oct-03	17.06	-28.55	5.66	45.06	7.96	
68	Cuphophyllus	virgineus	Wales	Waunfawr	52.4149,-4.0476	02-Nov-01	10.90	-26.54	5.16	41.83	8.11	
69	Cuphophyllus	virgineus	Wales	Waunfawr	52.4149,-4.0476	02-Nov-01	11.77	-27.73	4.61	45.62	9.89	
70	Cuphophyllus	virgineus	Wales	Waunfawr	52.4149,-4.0476	02-Nov-01	12.11	-27.04	6.07	44.10	7.26	
71	Cuphophyllus	virgineus	Wales	Waunfawr	52.4149,-4.0476	02-Nov-01	12.76	-28.17	4.80	44.27	9.22	
72	Cuphophyllus	virgineus	Wales	Porthmadog	52.9115,-4.0997	20-Sep-03	W14.77	W-26.56	W5.36	W43.30	W8.08	WOODLAND
73	Hygrocybe	cantharellus	Wales	Llyn Cwm Dulyn	53.0214,-4.2421	05-Sep-10	M0.50	M-28.78	M8.00	M43.91	M5.90	MOSS
74	Hygrocybe	cantharellus	Wales	Llyn Cwm Dulyn	53.0214,-4.2421	05-Sep-10	M1.82	M-27.23	M10.07	M43.73	M4.34	MOSS
75	Hygrocybe	chlorophana	Wales	Aberystwyth University	52.4194,-4.0683	01-Oct-01	14.19	-29.05	8.78	44.80	5.10	
76	Hygrocybe	chlorophana	Wales	Aberystwyth University	52.4194,-4.0683	01-Oct-01	15.97	-28.75	7.88	41.74	5.30	
77	Hygrocybe	chlorophana	Wales	Aberystwyth University	52.4194,-4.0683	01-Oct-01	16.48	-28.74	8.75	44.61	5.10	
78	Hygrocybe	chlorophana	Wales	Aberystwyth University	52.4194,-4.0683	01-Oct-01	17.09	-29.24	9.12	44.10	4.84	
79	Hygrocybe	chlorophana	Wales	Llanigon	52.0527,-3.1463	05-Jun-04	W12.21	W-25.79	W7.60	W45.22	W5.95	WOODLAND
80	Hygrocybe	chlorophana	Wales	Bangor	53.2179,-4.1664	14-May-04	W13.22	W-29.01	W7.89	W45.91	W5.82	WOODLAND
81	Hygrocybe	citrinovirens	Wales	Ystumtuen	52.4021,-3.8672	17-Oct-02	13.63	-28.85	5.89	42.17	7.16	
82	Hygrocybe	coccinea	Wales	Nebo	52.2652,-4.1317	27-Nov-02	10.93	-30.14	6.16	43.32	7.03	
83	Hygrocybe	coccinea	Wales	Waunfawr	52.4149,-4.0476	05-Dec-02	11.13	-28.69	7.14	46.38	6.50	
84	Hygrocybe	coccinea	Wales	Ystumtuen	52.4021,-3.8672	06-Nov-02	13.62	-27.94	7.24	43.26	5.98	
85	Hygrocybe	glutinipes	Wales	Waunfawr	52.4149,-4.0476	11-Feb-01	19.52	-28.06	5.43	44.74	8.24	
86	Hygrocybe	quieta	Wales	Llanigon	52.0527,-3.1463	10-Aug-03	W16.48	W-27.27	W6.23	W44.97	W7.22	WOODLAND

87	Neohygrocybe	nitrata	Wales	Bangor	53.1629, -4.1236	11-Oct-06	15.90	-29.52	8.45	43.40	5.1	
88	Porpolomopsis	calyptriformis	Wales	Abergorlech	51.9834,-4.0615	16-Nov-02	17.07	-29.06	6.96	43.76	6.29	
89	Porpolomopsis	calyptriformis	Wales	Aberystwyth University	52.4194,-4.0683	4-Dec-01	14.93	-27.98	5.33	42.46	7.97	
90	Porpolomopsis	calyptriformis	Wales	Aberystwyth University	52.4194,-4.0683	4-Dec-01	15.48	-27.14	7.41	43.13	5.82	
91	Porpolomopsis	calyptriformis	Wales	Disgwylfa, Epynt	52.0829,-3.4680	23-Nov-02	16.55	-29.55	7.27	44.65	6.14	
92	Porpolomopsis	calyptriformis	Wales	Waunfawr	52.4149,-4.0476	2-Nov-01	14.23	-28.97	4.60	45.13	9.82	
93	Porpolomopsis	calyptriformis	Wales	Y Fron	53.0657,-4.2355	12-Oct-02	14.27	-29.44	7.57	43.62	5.76	
						Mean	14.87	-28.85	6.32	43.75	7.25	
						SD	1.97	0.92	1.37	1.48	1.62	
						COUNT	36	36	36	36	36	
						Min	10.90	-30.40	3.29	39.13	4.84	
						Max	19.52	-26.54	9.12	46.51	11.90	
	NORTHERN EUROPE B (ENGLAND) UK											
94	Cuphophyllus	lacmus	England	Lundy	51.1999,-4.6744	11-Nov-04	H9.47	H-28.92	H6.16	H43.11	H6.99	IN HEATHER
95	Cuphophyllus	lacmus	England	Lundy	51.1999,-4.6738	11-Nov-04	H9.66	H-27.50	H5.83	H39.77	H6.82	IN HEATHER
96	Cuphophyllus	pratensis	England	Cocklepark	55.2174,-1.6838	20-Oct-04	16.23	-29.56	5.64	43.63	7.74	
97	Cuphophyllus	pratensis	England	Park Grass	51.8040,-0.3722	19-Nov-04	17.20	-29.72	5.27	43.41	8.24	
98	Cuphophyllus	pratensis	England	Park Grass	51.8040,-0.3722	19-Nov-04	18.77	-29.45	6.52	41.92	6.43	
99	Cuphophyllus	pratensis	England	Clitheroe	53.8067,-2.4294	24-Oct-02	18.82	-28.29	7.17	43.06	6.01	
100	Cuphophyllus	pratensis	England	Park Grass	51.8040,-0.3722	19-Nov-04	19.64	-30.28	6.35	43.08	6.78	
101	Cuphophyllus	pratensis	England	Clitheroe	53.8067,-2.4294	10-Oct-02	19.71	-27.92	6.35	42.98	6.77	
102	Cuphophyllus	pratensis	England	Clitheroe	53.8067,-2.4294	24-Oct-02	19.76	-29.77	7.43	42.39	5.71	
103	Cuphophyllus	virgineus	England	Cocklepark	55.2174,-1.6838	4-Nov-03	14.26	-28.54	4.97	43.79	8.81	
104	Cuphophyllus	virgineus	England	Cocklepark	55.2174,-1.6838	4-Nov-03	14.82	-29.65	4.83	45.34	9.39	
105	Cuphophyllus	virgineus	England	Cocklepark	55.2174,-1.6838	20-Oct-04	15.96	-30.21	5.92	42.94	7.25	
106	Cuphophyllus	virgineus	England	Lundy	51.1694,-4.6720	10-Nov-04	16.37	-27.39	5.26	43.93	8.35	
107	Gliophorus	psittacinus	England	Cocklepark	55.2174,-1.6838	26-Nov-07	15.85	-29.61	4.84	31.36	6.5	
108	Gliophorus	psittacinus	England	Cocklepark	55.2174,-1.6838	26-Nov-07	20.94	-29.26	8.21	45.70	5.6	
109	Hygrocybe	citrinovirens	England	Clitheroe	53.8067,-2.4294	9-Nov-02	13.68	-28.33	6.90	42.19	6.11	
110	Hygrocybe	coccinea	England	Cocklepark	55.2174,-1.6838	20-Oct-04	14.19	-28.99	7.05	44.43	6.30	
111	Hygrocybe	punicea	England	Cocklepark	55.2174,-1.6838	20-Oct-04	11.86	-28.59	6.35	45.33	7.14	
112	Hygrocybe	punicea	England	Park Grass	51.8040,-0.3722	19-Nov-04	13.09	-29.62	6.98	43.02	6.16	
113	Hygrocybe	punicea	England	Park Grass	51.8040,-0.3722	19-Nov-04	13.81	-29.48	6.72	45.64	6.79	
114	Hygrocybe	punicea	England	Park Grass	51.8040,-0.3722	5-Nov-08	13.93	-29.59	8.86	48.09	5.4	
115	Hygrocybe	punicea	England	Park Grass	51.8040,-0.3722	5-Nov-08	14.01	-29.84	9.72	43.62	4.5	
116	Hygrocybe	punicea	England	Park Grass	51.8040,-0.3722	5-Nov-08	14.83	-30.59	8.35	44.69	5.4	
117	Hygrocybe	punicea	England	Park Grass	51.8040,-0.3722	19-Nov-04	15.20	-28.21	7.24	45.21	6.24	
118	Hygrocybe	punicea	England	Park Grass	51.8040,-0.3722	5-Nov-08	16.90	-29.73	8.72	44.91	5.1	
119	Hygrocybe	punicea	England	Lundy	51.1694,-4.6720	11-Nov-04	18.01	-28.12	7.49	45.69	6.10	
120	Porpolomopsis	calyptriformis	England	Clitheroe	53.8067,-2.4294	10-Oct-02	16.71	-30.20	5.66	41.88	7.40	
121	Porpolomopsis	calyptriformis	England	Clitheroe	53.8067,-2.4294	10-Oct-02	17.83	-28.77	7.09	44.81	6.32	
						Mean	16.25	-29.22	6.77	43.58	6.63	
						SD	2.40	0.83	1.30	2.88	1.16	
						COUNT	26	26	26	26	26	
						Min	11.86	-30.59	4.83	31.36	4.49	
						Max	20.94	-27.39	9.72	48.09	9.39	
	NORTHERN EUROPE (C) GERMANY											
122	Cuphophyllus	flavipes	Germany	Amorbach	49.6460, 9.2010	1-Oct-08	14.39	-30.12	5.80	41.67	7.19	
123	Cuphophyllus	fornicata	Germany	Amorbach	49.6460, 9.2010	2-Oct-08	19.67	-29.98	6.44	43.70	6.78	
124	Cuphophyllus	pratensis	Germany	Amorbach	49.6460, 9.2010	4-Oct-08	15.23	-29.86	6.80	41.61	6.12	
125	Cuphophyllus	virgineus	Germany	Amorbach	49.6460, 9.2010	8-Oct-08	13.71	-28.89	6.89	44.60	6.47	
126	Cuphophyllus	virgineus	Germany	Amorbach	49.6460, 9.2010	6-Oct-08	14.82	-29.16	6.09	44.17	7.26	
127	Gliophorus	psittacinus	Germany	Amorbach	49.6460, 9.2010	12-Oct-08	15.99	-28.56	7.07	45.86	6.49	
128	Hygrocybe	aurantiosplendens	Germany	Amorbach	49.6460, 9.2010	1-Oct-08	13.08	-28.93	6.87	39.80	5.79	
129	Hygrocybe	chlorophana	Germany	Amorbach	49.6460, 9.2010	7-Oct-08	13.62	-29.00	8.78	43.95	5.01	
130	Hygrocybe	coccinea	Germany	Amorbach	49.6460, 9.2010	10-Oct-08	13.81	-28.86	8.18	45.73	5.59	
131	Hygrocybe	insipida	Germany	Amorbach	49.6460, 9.2010	11-Oct-08	12.10	-28.74	7.57	42.86	5.66	
132	Hygrocybe	mucronella	Germany	Amorbach	49.6460, 9.2010	9-Oct-08	19.23	-28.89	6.51	45.71	7.02	
133	Cuphophyllus	virgineus	Germany	Amorbach	49.6460, 9.2010	7-Nov-15	17.43	-30.79	4.94	44.78	9.07	Transect1 A1
134	Cuphophyllus	virgineus	Germany	Amorbach	49.6460, 9.2010	7-Nov-15	16.90	-30.48	5.90	43.81	7.42	Transect1 A2
135	Cuphophyllus	virgineus	Germany	Amorbach	49.6460, 9.2010	7-Nov-15	15.31	-30.06	4.67	44.82	9.60	Transect1 A3
136	Cuphophyllus	virgineus	Germany	Amorbach	49.6460, 9.2010	7-Nov-15	14.67	-30.55	4.65	44.08	9.49	Transect1 A4
137	Cuphophyllus	virgineus	Germany	Amorbach	49.6460, 9.2010	7-Nov-15	15.62	-30.58	4.86	44.70	9.20	Transect1 A5
138	Cuphophyllus	virgineus	Germany	Amorbach	49.6460, 9.2010	7-Nov-15	15.62	-30.14	5.44	43.94	8.08	Transect1 A6
139	Cuphophyllus	virgineus	Germany	Amorbach	49.6460, 9.2010	7-Nov-15	14.24	-30.13	4.82	44.21	9.18	Transect1 A7
140	Cuphophyllus	virgineus	Germany	Amorbach	49.6460, 9.2010	7-Nov-15	15.32	-29.97	5.63	43.79	7.77	Transect1 A8
141	Cuphophyllus	virgineus	Germany	Amorbach	49.6460, 9.2010	7-Nov-15	15.11	-30.02	4.96	44.14	8.90	Transect1 A9
142	Cuphophyllus	virgineus	Germany	Amorbach	49.6460, 9.2010	7-Nov-15	14.70	-29.76	5.34	44.25	8.29	Transect1 A10
143	Cuphophyllus	virgineus	Germany	Amorbach	49.6460, 9.2010	7-Nov-15	15.69	-30.07	5.74	44.10	7.68	Transect1 A11
144	Cuphophyllus	virgineus	Germany	Amorbach	49.6460, 9.2010	7-Nov-15	14.56	-30.05	4.64	43.29	9.34	Transect1 A12
145	Cuphophyllus	virgineus	Germany	Amorbach	49.6460, 9.2010	7-Nov-15	14.30	-30.07	4.75	44.71	9.42	Transect1 A13
146	Cuphophyllus	virgineus	Germany	Amorbach	49.6460, 9.2010	7-Nov-15	14.28	-30.24	4.86	45.71	9.41	Transect1 A14
147	Cuphophyllus	virgineus	Germany	Amorbach	49.6460, 9.2010	7-Nov-15	15.03	-29.88	5.36	44.31	8.26	Transect1 A15
148	Cuphophyllus	virgineus	Germany	Amorbach	49.6460, 9.2010	7-Nov-15	12.99	-30.47	4.97	43.74	8.79	Transect2 B1
149	Cuphophyllus	virgineus	Germany	Amorbach	49.6460, 9.2010	7-Nov-15	14.90	-30.58	4.80	42.32	8.81	Transect2 B2
150	Cuphophyllus	virgineus	Germany	Amorbach	49.6460, 9.2010	7-Nov-15	14.08	-30.39	5.04	44.38	8.80	Transect2 B3
151	Cuphophyllus	virgineus	Germany	Amorbach	49.6460, 9.2010	7-Nov-15	14.41	-30.62	5.02	44.22	8.81	Transect2 B4
152	Cuphophyllus	virgineus	Germany	Amorbach	49.6460, 9.2010	7-Nov-15	14.19	-30.58	5.20	44.63	8.58	Transect2 B5
153	Cuphophyllus	virgineus	Germany	Amorbach	49.6460, 9.2010	7-Nov-15	14.37	-30.83	5.14	44.27	8.61	Transect2 B6
154	Cuphophyllus	virgineus	Germany	Amorbach	49.6460, 9.2010	7-Nov-15	12.96	-31.11	4.86	44.85	9.23	Transect2 B7
155	Cuphophyllus	virgineus	Germany	Amorbach	49.6460, 9.2010	7-Nov-15	12.62	-30.80	4.44	44.52	10.02	Transect2 B8
156	Cuphophyllus	virgineus	Germany	Amorbach	49.6460, 9.2010	7-Nov-15	12.83	-30.97	4.92	45.29	9.21	Transect2 B9
157	Cuphophyllus	virgineus	Germany	Amorbach	49.6460, 9.2010	7-Nov-15	11.53	-30.93	5.99	45.14	7.53	Transect2 B10
158	Hygrocybe	coccinea	Germany	Amorbach	49.6460, 9.2010	7-Nov-15	14.16	-30.65	6.45	46.40	7.19	Fairy ring R1
159	Hygrocybe	coccinea	Germany	Amorbach	49.6460, 9.2010	7-Nov-15	13.85	-30.32	6.91	45.85	6.63	Fairy ring R2
160	Hygrocybe	coccinea	Germany	Amorbach	49.6460, 9.2010	7-Nov-15	13.67	-30.48	6.98	45.12	6.46	Fairy ring R3
161	Hygrocybe	coccinea	Germany	Amorbach	49.6460, 9.2010	7-Nov-15	13.25	-30.88	6.21	45.56	7.34	Fairy ring R4
162	Hygrocybe	coccinea	Germany	Amorbach	49.6460, 9.2010	7-Nov-15	11.77	-30.65	6.11	44.89	7.34	Fairy ring R5
163	Hygrocybe	coccinea	Germany	Amorbach	49.6460, 9.2010	7-Nov-15	14.29	-31.29	5.41	42.89	7.93	Fairy ring R6
164	Hygrocybe	coccinea	Germany	Amorbach	49.6460, 9.2010	7-Nov-15	11.68	-30.70	6.92	46.65	6.74	Fairy ring R7
165	Hygrocybe	coccinea	Germany	Amorbach	49.6460, 9.2010	7-Nov-15	12.05	-31.02	6.01	42.63	7.10	Fairy ring R8
166	Hygrocybe	coccinea	Germany	Amorbach	49.6460, 9.2010	7-Nov-15	12.89	-30.78	6.52	45.93	7.04	Fairy ring R9
167	Hygrocybe	coccinea	Germany	Amorbach	49.6460, 9.2010	7-Nov-15	14.40	-30.45	6.46	45.89	7.11	Fairy ring R10
168	Hygrocybe	coccinea	Germany	Amorbach	49.6460, 9.2010	7-Nov-15	13.50	-30.46	6.62	45.88	6.93	Fairy ring R11
169	Hygrocybe	coccinea	Germany	Amorbach	49.6460, 9.2010	7-Nov-15	13.17	-31.04	5.78	44.84	7.76	Fairy ring R12
170	Hygrocybe	coccinea	Germany	Amorbach	49.6460, 9.2010	7-Nov-15	11.76	-30.85	6.00	45.68	7.61	Fairy ring R13
171	Hygrocybe	coccinea	Germany	Amorbach	49.6460, 9.2010	7-Nov-15	12.37	-31.12	6.02	45.40	7.54	Fairy ring R14
172	Hygrocybe	coccinea	Germany	Amorbach	49.6460, 9.2010	7-Nov-15	13.46	-30.40	7.42	45.87	6.18	Fairy ring R15
173	Hygrocybe	coccinea	Germany	Amorbach	49.6460, 9.2010	7-Nov-15	11.41	-30.61	6.13	45.09	7.36	Fairy ring R16
174	Cuphophyllus	virgineus (ring)	Germany	Amorbach	49.6452,9.2007	31/10/15	13.34	-29.06	4.20	41.28	9.82	Fairy ring

175	Cuphophyllus	virgineus (ring)	Germany	Amorbach	49.6452,9.2007	31/10/15	13.75	-29.36	4.72	42.19	8.93	Fairy ring
176	Cuphophyllus	virgineus (ring)	Germany	Amorbach	49.6452,9.2007	31/10/15	12.51	-29.51	4.27	41.54	9.74	Fairy ring
177	Cuphophyllus	virgineus (ring)	Germany	Amorbach	49.6452,9.2007	31/10/15	11.98	-29.60	3.76	41.00	10.91	Fairy ring
178	Cuphophyllus	virgineus (ring)	Germany	Amorbach	49.6452,9.2007	31/10/15	13.20	-29.03	4.37	42.54	9.74	Fairy ring
179	Cuphophyllus	virgineus (ring)	Germany	Amorbach	49.6452,9.2007	31/10/15	14.21	-29.79	4.33	41.59	9.61	Fairy ring
180	Cuphophyllus	virgineus (ring)	Germany	Amorbach	49.6452,9.2007	31/10/15	13.00	-29.26	4.28	42.70	9.97	Fairy ring
181	Cuphophyllus	virgineus (ring)	Germany	Amorbach	49.6452,9.2007	31/10/15	13.14	-28.98	4.07	41.07	10.10	Fairy ring
182	Cuphophyllus	virgineus (ring)	Germany	Amorbach	49.6452,9.2007	31/10/15	13.05	-29.64	4.41	42.11	9.55	Fairy ring
183	Cuphophyllus	virgineus (ring)	Germany	Amorbach	49.6452,9.2007	31/10/15	12.38	-29.08	4.47	42.15	9.44	Fairy ring
184	Cuphophyllus	virgineus (ring)	Germany	Amorbach	49.6452,9.2007	31/10/15	13.17	-29.05	4.52	42.41	9.37	Fairy ring
185	Cuphophyllus	virgineus (ring)	Germany	Amorbach	49.6452,9.2007	31/10/15	12.91	-29.28	4.38	42.79	9.78	Fairy ring
186	Cuphophyllus	virgineus (ring)	Germany	Amorbach	49.6452,9.2007	31/10/15	12.57	-29.51	3.87	42.50	10.97	Fairy ring
187	Cuphophyllus	virgineus (ring)	Germany	Amorbach	49.6452,9.2007	31/10/15	12.95	-28.98	4.37	42.52	9.73	Fairy ring
188	Cuphophyllus	virgineus (ring)	Germany	Amorbach	49.6452,9.2007	31/10/15	15.21	-29.50	5.04	41.63	8.27	Fairy ring
189	Cuphophyllus	virgineus (ring)	Germany	Amorbach	49.6452,9.2007	31/10/15	13.33	-29.22	4.46	41.78	9.36	Fairy ring
190	Cuphophyllus	virgineus (ring)	Germany	Amorbach	49.6452,9.2007	31/10/15	14.73	-29.45	5.01	42.77	8.54	Fairy ring
191	Cuphophyllus	virgineus (ring)	Germany	Amorbach	49.6452,9.2007	31/10/15	13.82	-29.62	4.33	42.30	9.77	Fairy ring
192	Cuphophyllus	virgineus (ring)	Germany	Amorbach	49.6452,9.2007	31/10/15	13.57	-29.30	4.52	41.55	9.19	Fairy ring
193	Cuphophyllus	virgineus (ring)	Germany	Amorbach	49.6452,9.2007	31/10/15	15.39	-28.96	4.59	41.57	9.06	Fairy ring
194	Cuphophyllus	virgineus	Germany	Amorbach	49.6452,9.2007	31/10/15	16.54	-28.70	4.61	42.19	9.16	
195	Cuphophyllus	virgineus	Germany	Amorbach	49.6452,9.2007	31/10/15	17.52	-28.48	4.77	41.86	8.78	
196	Cuphophyllus	virgineus	Germany	Amorbach	49.6452,9.2007	31/10/15	15.36	-28.65	4.41	42.84	9.72	
197	Cuphophyllus	virgineus	Germany	Amorbach	49.6452,9.2007	31/10/15	15.00	-28.25	4.18	42.87	10.25	
198	Cuphophyllus	virgineus	Germany	Amorbach	49.6452,9.2007	31/10/15	17.01	-28.64	4.17	41.60	9.97	
199	Cuphophyllus	virgineus	Germany	Amorbach	49.6452,9.2007	31/10/15	14.52	-28.24	4.26	42.75	10.03	
200	Cuphophyllus	virgineus	Germany	Amorbach	49.6452,9.2007	31/10/15	15.66	-29.81	4.39	41.41	9.43	
201	Cuphophyllus	virgineus	Germany	Amorbach	49.6452,9.2007	31/10/15	13.19	-28.81	3.91	42.52	10.87	
202	Cuphophyllus	virgineus	Germany	Amorbach	49.6452,9.2007	31/10/15	17.12	-28.73	4.69	41.91	8.94	
203	Cuphophyllus	virgineus	Germany	Amorbach	49.6464,9.2019	7/11/15	14.67	-27.50	4.94	40.02	8.10	
204	Cuphophyllus	virgineus	Germany	Amorbach	49.6464,9.2019	7/11/15	16.57	-28.12	4.74	41.01	8.66	
205	Cuphophyllus	virgineus	Germany	Amorbach	49.6464,9.2019	7/11/15	16.61	-28.36	4.54	41.04	9.03	
206	Cuphophyllus	virgineus	Germany	Amorbach	49.6464,9.2019	7/11/15	16.54	-28.55	4.93	42.27	8.57	
207	Cuphophyllus	virgineus	Germany	Amorbach	49.6464,9.2019	7/11/15	14.92	-28.27	4.64	40.17	8.66	
208	Cuphophyllus	virgineus	Germany	Amorbach	49.6464,9.2019	7/11/15	16.91	-27.71	5.60	42.88	7.65	
209	Cuphophyllus	virgineus	Germany	Amorbach	49.6464,9.2019	7/11/15	17.50	-28.27	5.00	41.20	8.23	
210	Cuphophyllus	virgineus	Germany	Amorbach	49.6464,9.2019	7/11/15	16.31	-28.10	4.27	40.89	9.58	
211	Cuphophyllus	virgineus	Germany	Amorbach	49.6464,9.2019	7/11/15	17.57	-28.29	4.95	41.77	8.44	
212	Cuphophyllus	virgineus	Germany	Amorbach	49.6453,9.2009	7/11/15	15.90	-28.85	5.28	41.37	7.84	
213	Cuphophyllus	virgineus	Germany	Amorbach	49.6453,9.2009	7/11/15	15.20	-28.21	5.39	42.10	7.81	
214	Cuphophyllus	virgineus	Germany	Amorbach	49.6453,9.2009	7/11/15	15.70	-28.70	5.16	42.81	8.30	
215	Cuphophyllus	virgineus	Germany	Amorbach	49.6453,9.2009	7/11/15	15.83	-28.40	4.87	40.97	8.41	
216	Cuphophyllus	virgineus	Germany	Amorbach	49.6453,9.2009	7/11/15	14.68	-29.58	4.43	42.32	9.55	
217	Cuphophyllus	virgineus	Germany	Amorbach	49.6453,9.2009	7/11/15	13.90	-31.19	3.83	39.10	10.22	
218	Cuphophyllus	virgineus	Germany	Amorbach	49.6453,9.2009	7/11/15	15.50	-29.08	4.03	41.96	10.40	
219	Cuphophyllus	virgineus	Germany	Amorbach	49.6453,9.2009	7/11/15	14.55	-28.67	3.71	42.01	11.33	
220	Cuphophyllus	virgineus	Germany	Amorbach	49.6453,9.2009	7/11/15	14.65	-29.11	3.58	39.18	10.96	
221	Cuphophyllus	virgineus	Germany	Amorbach	49.6453,9.2009	7/11/15	13.76	-28.94	3.70	40.74	11.02	
222	Cuphophyllus	virgineus	Germany	Amorbach	49.6453,9.2009	7/11/15	15.20	-29.03	5.17	43.00	8.31	
223	Cuphophyllus	virgineus	Germany	Amorbach	49.6453,9.2009	7/11/15	15.89	-28.33	5.01	41.99	8.39	
224	Cuphophyllus	virgineus	Germany	Amorbach	49.6453,9.2009	7/11/15	14.20	-28.91	3.81	42.32	11.12	
225	Cuphophyllus	virgineus	Germany	Amorbach	49.6453,9.2009	7/11/15	14.87	-28.93	4.43	44.70	10.09	
226	Cuphophyllus	virgineus	Germany	Amorbach	49.6453,9.2009	7/11/15	14.51	-30.09	4.99	42.24	8.47	
227	Cuphophyllus	virgineus	Germany	Amorbach	49.6453,9.2009	7/11/15	13.69	-29.35	3.81	40.42	10.61	
228	Hygrocybe	coccinea	Germany	Amorbach	49.6453,9.2009	7/11/15	13.53	-29.66	5.56	42.78	7.69	
229	Hygrocybe	coccinea	Germany	Amorbach	49.6453,9.2009	7/11/15	13.19	-29.68	5.80	43.35	7.47	
230	Hygrocybe	coccinea	Germany	Amorbach	49.6453,9.2009	7/11/15	14.08	-29.31	5.69	44.07	7.75	
231	Hygrocybe	coccinea	Germany	Amorbach	49.6453,9.2009	7/11/15	14.14	-29.44	4.80	43.04	8.96	
232	Hygrocybe	coccinea	Germany	Amorbach	49.6453,9.2009	7/11/15	12.79	-29.73	5.39	42.95	7.98	
233	Hygrocybe	coccinea	Germany	Amorbach	49.6453,9.2009	7/11/15	13.72	-29.39	4.93	41.69	8.45	
234	Hygrocybe	coccinea	Germany	Amorbach	49.6453,9.2009	7/11/15	13.43	-27.97	7.09	43.56	6.14	
235	Hygrocybe	coccinea	Germany	Amorbach	49.6453,9.2009	7/11/15	13.77	-29.68	6.30	44.55	7.07	
236	Hygrocybe	coccinea	Germany	Amorbach	49.6453,9.2009	7/11/15	13.89	-29.09	5.40	41.95	7.77	
237	Hygrocybe	coccinea	Germany	Amorbach	49.6453,9.2009	7/11/15	14.36	-29.57	5.92	43.80	7.39	
238	Hygrocybe	coccinea	Germany	Amorbach	49.6453,9.2009	7/11/15	14.45	-29.37	5.88	43.00	7.31	
239	Hygrocybe	coccinea	Germany	Amorbach	49.6453,9.2009	7/11/15	13.58	-29.37	6.78	44.83	6.62	
240	Hygrocybe	coccinea	Germany	Amorbach	49.6453,9.2009	7/11/15	13.73	-29.53	5.77	43.16	7.48	
241	Hygrocybe	coccinea	Germany	Amorbach	49.6453,9.2009	7/11/15	12.77	-29.50	6.74	43.88	6.51	
242	Hygrocybe	coccinea	Germany	Amorbach	49.6453,9.2009	7/11/15	13.23	-29.06	6.27	42.45	6.77	
243	Hygrocybe	chlorophana	Germany	Amorbach	49.6453,9.2009	7/11/15	14.53	-28.56	7.21	42.97	5.96	
244	Hygrocybe	chlorophana	Germany	Amorbach	49.6453,9.2009	7/11/15	15.15	-28.29	6.87	41.61	6.06	
245	Hygrocybe	chlorophana	Germany	Amorbach	49.6453,9.2009	7/11/15	14.78	-29.48	6.44	40.54	6.30	
246	Hygrocybe	chlorophana	Germany	Amorbach	49.6453,9.2009	7/11/15	15.24	-28.96	7.56	42.88	5.67	
247	Hygrocybe	chlorophana	Germany	Amorbach	49.6453,9.2009	7/11/15	14.71	-29.27	7.10	42.04	5.92	
248	Hygrocybe	chlorophana	Germany	Amorbach	49.6453,9.2009	7/11/15	14.89	-28.94	7.98	42.29	5.30	
249	Hygrocybe	cantharellus	Germany	Amorbach	49.6452,9.2010	25/10/14	6.08	-31.16	5.91	42.85	7.25	
250	Cuphophyllus	pratensis	Germany	Bischbrunn, Spessart	49.8882,9.4678	6/11/15	17.05	-29.94	4.50	41.57	9.24	
251	Cuphophyllus	pratensis	Germany	Bischbrunn, Spessart	49.8882,9.4678	6/11/15	15.99	-30.36	5.22	41.06	7.86	
252	Cuphophyllus	pratensis	Germany	Bischbrunn, Spessart	49.8882,9.4678	6/11/15	16.73	-29.93	4.44	41.21	9.28	
253	Cuphophyllus	pratensis	Germany	Bischbrunn, Spessart	49.8882,9.4678	6/11/15	15.58	-30.11	4.11	40.15	9.77	
254	Cuphophyllus	pratensis	Germany	Bischbrunn, Spessart	49.8882,9.4678	6/11/15	16.65	-29.85	4.17	41.74	10.02	
255	Cuphophyllus	pratensis	Germany	Bischbrunn, Spessart	49.8882,9.4678	6/11/15	16.73	-29.84	4.33	45.69	10.54	
256	Cuphophyllus	pratensis	Germany	Bischbrunn, Spessart	49.8882,9.4678	6/11/15	15.50	-30.07	4.25	40.85	9.61	
257	Cuphophyllus	pratensis	Germany	Bischbrunn, Spessart	49.8882,9.4678	6/11/15	15.23	-29.89	4.90	41.23	8.41	
258	Cuphophyllus	pratensis	Germany	Bischbrunn, Spessart	49.8882,9.4678	6/11/15	16.53	-29.80	4.03	41.43	10.29	
259	Cuphophyllus	pratensis	Germany	Bischbrunn, Spessart	49.8882,9.4678	6/11/15	16.55	-29.86	4.39	41.57	9.47	
260	Cuphophyllus	pratensis	Germany	Bischbrunn, Spessart	49.8882,9.4678	6/11/15	16.75	-29.78	4.55	41.83	9.20	
261	Cuphophyllus	pratensis	Germany	Bischbrunn, Spessart	49.8882,9.4678	6/11/15	15.56	-29.95	3.82	40.67	10.65	
262	Cuphophyllus	pratensis	Germany	Bischbrunn, Spessart	49.8882,9.4678	6/11/15	17.09	-30.45	5.23	42.87	8.20	
263	Cuphophyllus	pratensis	Germany	Bischbrunn, Spessart	49.8882,9.4678	6/11/15	16.99	-29.71	4.17	40.57	9.73	
264	Cuphophyllus	pratensis	Germany	Bischbrunn, Spessart	49.8882,9.4678	6/11/15	17.77	-29.34	5.39	41.68	7.73	
265	Cuphophyllus	pratensis	Germany	Bischbrunn, Spessart	49.8882,9.4678	6/11/15	16.39	-29.99	4.23	41.19	9.74	
266	Cuphophyllus	pratensis	Germany	Bischbrunn, Spessart	49.8882,9.4678	6/11/15	16.03	-30.18	5.80	41.59	7.16	
267	Cuphophyllus	pratensis	Germany	Bischbrunn, Spessart	49.8882,9.4678	6/11/15	16.55	-30.34	4.64	41.55	8.96	
268	Cuphophyllus	pratensis	Germany	Bischbrunn, Spessart	49.8882,9.4678	6/11/15	14.93	-30.31	3.90	41.27	10.57	
269	Cuphophyllus	pratensis	Germany	Bischbrunn, Spessart	49.8882,9.4678	6/11/15	15.22	-30.09	4.13	40.81	9.87	
270	Cuphophyllus	pratensis	Germany	Bischbrunn, Spessart	49.8882,9.4678	6/11/15	16.54	-30.30	4.51	41.09	9.12	
271	Cuphophyllus	pratensis	Germany	Bischbrunn, Spessart	49.8882,9.4678	6/11/15	16.14	-29.84	4.17	41.10	9.85	
272	Cuphophyllus	pratensis	Germany	Bischbrunn, Spessart	49.8882,9.4678	6/11/15	15.82	-30.16	4.12	40.84	9.91	
273	Cuphophyllus	pratensis	Germany	Bischbrunn, Spessart	49.8882,9.4678	6/11/15	15.68	-30.42	4.59	40.64	8.85	
274	Cuphophyllus	virgineus	Germany	Bischbrunn, Spessart	49.8873,9.4707	6/11/15	12.95	-30.16	3.31	40.32	12.17	
275	Cuphophyllus	virgineus	Germany	Bischbrunn, Spessart	49.8873,9.4707	6/11/15	16.96	-29.41	5.53	43.61	7.89	
276	Cuphophyllus	virgineus	Germany	Bischbrunn, Spessart	49.8873,9.4707	6/11/15	14.32	-30.73	3.76	42.32	11.26	
						Mean	14.63	-29.62	5.20	42.81	8.52	
						SD	1.72	0.84	1.07	1.68	1.45	
						COUNT	155	155	155	155	155	
						Min	6.08	-31.29	3.31	39.10	5.01	

						Max	19.67	-28.56	8.78	46.65	10.02	
	**NORTHERN EUROPE (D) ITALY**											
279	Hygrocybe	quieta	Italy	Teviggio, Liguria	44.3608,9.6004	16/10/15	13.91	-28.19	4.44	39.44	8.89	2x2m quadrat
280	Hygrocybe	quieta	Italy	Teviggio, Liguria	44.3608,9.6004	16/10/15	12.68	-28.35	4.63	39.66	8.56	2x2m quadrat
281	Hygrocybe	quieta	Italy	Teviggio, Liguria	44.3608,9.6004	16/10/15	13.77	-28.17	4.37	41.24	9.44	2x2m quadrat
282	Hygrocybe	quieta	Italy	Teviggio, Liguria	44.3608,9.6004	16/10/15	14.49	-28.31	4.58	41.49	9.05	2x2m quadrat
283	Hygrocybe	quieta	Italy	Teviggio, Liguria	44.3608,9.6004	16/10/15	13.29	-28.31	3.94	37.13	9.42	2x2m quadrat
284	Hygrocybe	quieta	Italy	Teviggio, Liguria	44.3608,9.6004	16/10/15	13.37	-28.42	5.26	40.67	7.74	2x2m quadrat
285	Hygrocybe	quieta	Italy	Teviggio, Liguria	44.3608,9.6004	16/10/15	13.58	-28.46	4.18	40.68	9.74	2x2m quadrat
286	Hygrocybe	quieta	Italy	Teviggio, Liguria	44.3608,9.6004	16/10/15	12.47	-28.42	5.34	41.24	7.72	2x2m quadrat
287	Hygrocybe	quieta	Italy	Teviggio, Liguria	44.3608,9.6004	16/10/15	13.73	-27.77	5.93	40.54	6.84	2x2m quadrat
288	Hygrocybe	quieta	Italy	Teviggio, Liguria	44.3608,9.6004	16/10/15	12.82	-27.86	4.83	40.33	8.36	2x2m quadrat
289	Hygrocybe	quieta	Italy	Teviggio, Liguria	44.3608,9.6004	16/10/15	14.18	-28.32	4.09	39.91	9.77	2x2m quadrat
290	Hygrocybe	quieta	Italy	Teviggio, Liguria	44.3608,9.6004	16/10/15	13.98	-27.90	4.51	41.00	9.08	2x2m quadrat
291	Hygrocybe	quieta	Italy	Teviggio, Liguria	44.3608,9.6004	16/10/15	13.87	-28.04	4.10	40.83	9.96	2x2m quadrat
292	Hygrocybe	quieta	Italy	Teviggio, Liguria	44.3608,9.6004	16/10/15	13.34	-27.89	6.30	42.63	6.77	2x2m quadrat
293	Hygrocybe	quieta	Italy	Teviggio, Liguria	44.3608,9.6004	16/10/15	13.78	-28.10	4.43	41.22	9.30	2x2m quadrat
294	Gliophorus	psittacinus	Italy	Verease, Liguri, Liguria	44.3745,9.5972	16/10/15	17.83	-29.69	5.05	38.01	7.53	
295	Cuphophyllus	virgineus	Italy	Verease, Liguri, Liguria	44.3765,9.6074	16/10/15	13.58	-29.08	4.84	39.36	8.13	
296	Cuphophyllus	virgineus	Italy	Verease, Liguri, Liguria	44.3765,9.6074	16/10/15	14.44	-28.23	6.18	40.82	6.60	
297	Cuphophyllus	virgineus	Italy	Verease, Liguri, Liguria	44.3765,9.6074	16/10/15	13.86	-29.09	4.96	40.22	8.11	
298	Cuphophyllus	virgineus	Italy	Verease, Liguri, Liguria	44.3765,9.6074	16/10/15	13.45	-29.01	4.55	40.38	8.88	
299	Cuphophyllus	virgineus	Italy	Verease, Liguri, Liguria	44.3765,9.6074	16/10/15	13.24	-29.76	4.45	40.43	9.09	
300	Gliophorus	psittacinus	Italy	Verease, Liguri, Liguria	44.3296,9.5786	17/10/15	17.43	-29.13	5.60	39.95	7.13	
301	Gliophorus	psittacinus	Italy	Verease, Liguri, Liguria	44.3296,9.5786	17/10/15	17.08	-29.08	5.34	37.85	7.08	
302	Gliophorus	psittacinus	Italy	Verease, Liguri, Liguria	44.3296,9.5786	17/10/15	17.11	-29.05	5.48	40.50	7.39	
						Mean	14.22	-28.53	4.89	40.23	8.36	
						SD	1.52	0.57	0.66	1.22	1.05	
						COUNT	24	24	24	24	24	
						Min	12.47	-29.76	3.94	37.13	6.60	
						Max	17.83	-27.77	6.30	42.63	9.96	
	**NORTHERN EUROPE (E) ICELAND**											
303	Cuphophyllus	pratensis	Iceland	Dalvík, Northeast Region	65.8912,-18.6200	1/9/16	11.57	-30.79	5.44	41.58	7.65	
304	Hygrocybe	chlorophana	Iceland	Dalvík, Northeast Region	65.8912,-18.6200	1/9/16	9.76	-29.62	5.03	43.93	8.73	
305	Hygrocybe	chlorophana	Iceland	Dalvík, Northeast Region	65.8912,-18.6200	1/9/16	8.08	-28.85	5.88	42.23	7.18	
306	Hygrocybe	coccinea	Iceland	Dalvík, Northeast Region	65.8912,-18.6200	2/9/20	9.00	-29.84	6.10	42.92	7.04	
307	Hygrocybe	punicea	Iceland	Dalvík, Northeast Region	65.8912,-18.6200	2/9/20	9.67	-29.43	4.46	40.18	9.00	
308	Hygrocybe	conica	Iceland	Seyðisfjörðuri, Eastern region	65.2735,-14.01239	15/9/20	11.36	-29.87	5.19	38.40	7.40	
309	Cuphophyllus	virgineus	Iceland	Seyðisfjörðuri, Eastern region	65.2807, -13.9950	12/9/16	12.44	-29.78	4.94	39.54	8.01	
310	Gliophorus	psittacinus	Iceland	Seyðisfjörðuri, Eastern region	65.2807, -13.9950	12/9/16	9.27	-29.72	4.10	40.61	9.91	
311	Hygrocybe	ceracea	Iceland	Seyðisfjörðuri, Eastern region	65.2807, -13.9950	12/9/16	7.95	-30.30	5.40	41.31	7.65	
312	Hygrocybe	quieta	Iceland	Seyðisfjörðuri, Eastern region	65.2807, -13.9950	13/9/20	12.60	-30.17	5.71	41.58	7.28	
						Mean	10.17	-29.84	5.23	41.23	7.98	
						SD	1.71	0.52	0.62	1.63	0.94	
						COUNT	10	10	10	10	10	
						Min	7.95	-30.79	4.10	38.40	7.04	
						Max	12.60	-28.85	6.10	43.93	9.91	
	**NON-EUROPEAN A (LA PALMA)**											
313	Cuphophyllus	colemaniana	La Palma	Los Tilos	28.7899,-17.8057	21-Oct-08	11.71	-28.64	6.19	43.77	7.07	
314	Cuphophyllus	pratensis	La Palma	Los Tilos	28.7899,-17.8057	16-Oct-08	17.46	-29.47	6.56	43.47	6.62	
315	Gliophorus	laeta	La Palma	Los Tilos	28.7899,-17.8057	19-Oct-08	14.12	-28.80	5.36	44.86	8.37	
316	Gliophorus	perplexus	La Palma	Los Tilos	28.7899,-17.8057	22-Oct-08	14.99	-28.46	6.37	47.09	7.39	
317	Gliophorus	perplexus	La Palma	Los Tilos	28.7899,-17.8057	25-Oct-08	15.13	-28.17	7.55	45.57	6.04	
318	Hygrocybe	coccinea	La Palma	Los Tilos	28.7899,-17.8057	26-Oct-08	10.46	-27.88	7.69	44.91	5.84	
319	Hygrocybe	coccinea	La Palma	Los Tilos	28.7899,-17.8057	27-Oct-08	12.16	-27.74	8.43	43.52	5.16	
320	Hygrocybe	coccinea	La Palma	Los Tilos	28.7899,-17.8057	20-Oct-08	17.64	-28.78	7.30	44.55	6.11	
321	Hygrocybe	insipida	La Palma	Los Tilos	28.7899,-17.8057	15-Oct-08	10.03	-29.47	8.24	44.25	5.37	
322	Hygrocybe	miniata	La Palma	Los Tilos	28.7899,-17.8057	18-Oct-08	8.54	-28.90	6.87	41.54	6.05	
323	Hygrocybe	quieta	La Palma	Los Tilos	28.7899,-17.8057	23-Oct-08	12.51	-28.27	6.88	43.64	6.35	
324	Hygrocybe	quieta	La Palma	Los Tilos	28.7899,-17.8057	24-Oct-08	21.00	-28.26	5.91	41.29	6.99	
325	Hygrocybe	reidii	La Palma	Los Tilos	28.7899,-17.8057	13-Oct-08	6.95	-29.83	7.50	44.36	5.91	
326	Neohygrocybe	nitrata	La Palma	Los Tilos	28.7899,-17.8057	14-Oct-08	12.70	-29.53	6.97	43.05	6.17	
327	Neohygrocybe	nitrata	La Palma	Los Tilos	28.7899,-17.8057	17-Oct-08	14.53	-29.04	6.70	42.63	6.36	
328	Hygrocybe	insipida	La Palma	Acropark La Palma	28.6191,-17.8230	6-Dec-15	9.19	-27.62	5.83	42.78	7.34	
						Mean	13.07	-28.68	6.90	43.83	6.45	
						SD	3.71	0.67	0.86	1.46	0.82	
						COUNT	16	16	16	16	16	
						Min	6.95	-29.83	5.36	41.29	5.16	
						Max	21.00	-27.62	8.43	47.09	8.37	
	**NON-EUROPEAN B (NEW ZEALAND)**											
330	Cuphophyllus	aurantiopallens	New Zealand	Puketui Valley	-37.0916,175.7331	May-08	13.99	-29.06	4.35	40.40	9.29	
331	Cuphophyllus	aurantiopallens	New Zealand	Murphy's Bush, Auckland	-36.9796,174.9195	May-08	19.46	-29.15	6.27	42.03	6.70	
332	Cuphophyllus	muritaiensis	New Zealand	Kaimai-Mamaku Forest	-37.5407,175.8706	May-08	14.70	-30.71	6.05	41.33	6.84	
333	Cuphophyllus	virgineus	New Zealand	Kaimai-Mamaku Forest	-37.5220,175.8780	May-02	15.66	-30.52	5.08	45.10	*8.88*	
334	Gliophorus	lilacipes	New Zealand	Haast	-43.8479,169.0581	May-02	13.70	-30.10	4.04	41.51	10.27	
335	Gliophorus	lilacipes	New Zealand	Kaimai-Mamaku Forest	-37.5407,175.8706	May-08	17.21	-31.18	4.73	43.98	9.30	
336	Gliophorus	luteoglutinosus	New Zealand	Kaimai-Mamaku Forest	-37.5407,175.8706	May-08	17.56	-25.47	5.03	41.07	8.17	
337	Gliophorus	versicolor	New Zealand	Kaimai-Mamaku Forest	-37.5407,175.8706	May-08	13.72	-27.19	4.59	39.37	8.58	
338	Gliophorus	viridis	New Zealand	Puketui Valley	-37.0916,175.7331	May-08	11.51	-27.56	5.46	44.43	8.14	
339	Gliophorus	viridis	New Zealand	Murphy's Bush, Auckland	-36.9796,174.9195	May-08	15.76	-28.72	4.62	41.72	9.04	
340	Humidicutis	couspicua	New Zealand	Hunua Ranges	-37.0682,175.0934	May-08	15.87	-27.92	4.19	39.28	9.38	
341	Humidicutis	pura	New Zealand	Kaimai-Mamaku Forest	-37.5407,175.8706	May-08	17.95	-23.93	5.43	38.73	7.14	
342	Humidicutis	rosella	New Zealand	Kaimai-Mamaku Forest	-37.5407,175.8706	May-08	12.90	-28.12	5.33	42.21	7.91	
343	Hygrocybe	blanda	New Zealand	Hunua Ranges	-37.0682,175.0934	May-08	8.93	-27.54	6.41	41.79	6.52	
344	Hygrocybe	blanda	New Zealand	Kaimai-Mamaku Forest	-37.5220,175.8780	May-02	15.46	-28.64	6.28	44.35	*7.06*	
345	Hygrocybe	blanda	New Zealand	Kaimai-Mamaku Forest	-37.5407,175.8706	May-08	20.68	-27.12	9.44	43.00	4.55	
346	Hygrocybe	cantharellus	New Zealand	Kaimai-Mamaku Forest	-37.5407,175.8706	May-08	1.79	-31.18	4.10	39.87	9.72	
347	Hygrocybe	cerinolutea	New Zealand	Kaimai-Mamaku Forest	-37.5407,175.8706	May-08	4.14	-24.50	6.93	43.19	6.23	
348	Hygrocybe	firma (red)	New Zealand	Kaimai-Mamaku Forest	-37.5220,175.8780	May-02	1.90	-30.69	7.74	44.07	*5.69*	
349	Hygrocybe	firma (red)	New Zealand	Kaimai-Mamaku Forest	-37.5407,175.8706	May-08	5.17	-30.61	5.56	42.47	7.64	
350	Hygrocybe	firma (yellow)	New Zealand	Kaimai-Mamaku Forest	-37.5220,175.8780	May-02	3.02	-30.43	7.24	45.05	*6.22*	
351	Hygrocybe	firma (yellow)	New Zealand	Puketui Valley	-37.0916,175.7331	May-08	5.90	-27.86	7.21	49.04	6.80	
352	Hygrocybe	julietae	New Zealand	Kaimai-Mamaku Forest	-37.5220,175.8780	May-02	15.65	-30.20	6.29	46.16	*7.34*	
353	Hygrocybe	julietae	New Zealand	Kaimai-Mamaku Forest	-37.5407,175.8706	May-08	19.40	-27.14	6.58	44.23	6.73	
354	Hygrocybe	keithgeorgei	New Zealand	Kaimai-Mamaku Forest	-37.5407,175.8706	May-08	7.81	-29.37	7.63	43.67	5.72	
355	Hygrocybe	keithgeorgei	New Zealand	Hunua Ranges	-37.0682,175.0934	May-08	10.03	-26.12	8.34	45.29	5.43	
356	Hygrocybe	lilaceolamellata	New Zealand	Hunua Ranges	-37.0682,175.0934	May-08	7.60	-26.31	6.66	42.39	6.36	
357	Hygrocybe	lilaceolamellata	New Zealand	Kaimai-Mamaku Forest	-37.5407,175.8706	May-08	12.72	-26.23	6.04	42.30	7.01	

358	Hygrocybe	miniata	New Zealand	Kaimai-Mamaku Forest	-37.7768,175.9511	May-08	2.38	-27.02	6.43	42.75	6.65	
359	Hygrocybe	procera	New Zealand	Kaimai-Mamaku Forest	-37.5407,175.8706	May-08	5.61	-28.92	5.10	40.19	7.88	
360	Hygrocybe	rubrocarnosa	New Zealand	Kaimai-Mamaku Forest	-37.5220,175.8780	May-02	2.47	-32.10	9.28	46.09	*4.97*	
361	Hygrocybe	rubrocarnosa	New Zealand	Kaimai-Mamaku Forest	-37.5407,175.8706	May-08	6.35	-29.83	7.20	42.45	5.90	
						Mean	11.16	-28.48	6.11	42.80	7.31	
						SD	5.91	2.05	1.42	2.28	1.45	
						COUNT	32	32	32	32	32	
						Min	1.79	-32.10	4.04	38.73	4.55	
						Max	20.68	-23.93	9.44	49.04	10.27	
	**NON-EUROPEAN C (ECUADOR)**											
362	Hygrocybe	sp. JNH698	Ecuador	Cuyabeno Reserve	0.0074,76.1473	14-Nov-88	13.67	-28.47	8.03	44.02	5.48	
363	Hygrocybe	sp. JNH1103	Ecuador	Cuyabeno Reserve	0.0074,76.1473	20-Nov-88	18.44	-24.15	6.93	40.05	5.78	
364	Hygrocybe	firma AFF JNH1079	Ecuador	Cuyabeno Reserve	0.0074,76.1473	20-Nov-88	11.03	-30.74	6.89	39.64	5.76	
						Mean	14.38	-27.79	7.28	41.24	5.67	
						SD	3.75	3.35	0.65	2.42	0.17	
						COUNT	3	3	3	3	3	
						Min	11.03	-30.74	6.89	39.64	5.48	
						Max	18.44	-24.15	8.03	44.02	5.78	
	**NON-EUROPEAN D (USA)**											
353	Cuphophyllus	lacmus	USA	Harvard Forest, MA	42.5129,-72.2138	nd	11.86	-28.59	nd	nd	nd	Seitzmann et al. (201
354	Cuphophyllus	sp. (HM020683) aff c	USA	Harvard Forest, MA	42.5129,-72.2138	nd	17.05	-28.53	nd	nd	nd	Seitzmann et al. (201
355	Gliophorus	laetus	USA	Harvard Forest, MA	42.5129,-72.2138	nd	5.05	-27.64	nd	nd	nd	Seitzmann et al. (201
356	Gliophorus	sp. (HM020676)	USA	Harvard Forest, MA	42.5129,-72.2138	nd	8.43	-27.41	nd	nd	nd	Seitzmann et al. (201
357	Humidicutis	auratocephalus	USA	Harvard Forest, MA	42.5129,-72.2138	nd	13.16	-26.64	nd	nd	nd	Seitzmann et al. (201
358	Humidicutis	auratocephalus	USA	Harvard Forest, MA	42.5129,-72.2138	nd	13.49	-27.31	nd	nd	nd	Seitzmann et al. (201
359	Humidicutis	auratocephalus	USA	Harvard Forest, MA	42.5129,-72.2138	nd	14.09	-27.02	nd	nd	nd	Seitzmann et al. (201
360	Humidicutis	auratocephalus	USA	Harvard Forest, MA	42.5129,-72.2138	nd	14.17	-27.98	nd	nd	nd	Seitzmann et al. (201
361	Humidicutis	auratocephalus	USA	Harvard Forest, MA	42.5129,-72.2138	nd	14.57	-27.10	nd	nd	nd	Seitzmann et al. (201
362	Humidicutis	auratocephalus	USA	Harvard Forest, MA	42.5129,-72.2138	nd	15.21	-27.24	nd	nd	nd	Seitzmann et al. (201
363	Humidicutis	auratocephalus	USA	Harvard Forest, MA	42.5129,-72.2138	nd	15.59	-28.04	nd	nd	nd	Seitzmann et al. (201
364	Humidicutis	auratocephalus	USA	Harvard Forest, MA	42.5129,-72.2138	nd	16.08	-25.62	nd	nd	nd	Seitzmann et al. (201
365	Humidicutis	auratocephalus	USA	Harvard Forest, MA	42.5129,-72.2138	nd	16.84	-27.61	nd	nd	nd	Seitzmann et al. (201
366	Humidicutis	auratocephalus	USA	Harvard Forest, MA	42.5129,-72.2138	nd	17.04	-27.31	nd	nd	nd	Seitzmann et al. (201
367	Hygrocybe	cf cantharellus	USA	Harvard Forest, MA	42.5129,-72.2138	nd	6.60	-28.45	nd	nd	nd	Seitzmann et al. (201
368	Hygrocybe	miniata f. longipes	USA	Harvard Forest, MA	42.5129,-72.2138	nd	6.24	-27.73	nd	nd	nd	Seitzmann et al. (201
369	Hygrocybe	sp.	USA	Harvard Forest, MA	42.5129,-72.2138	nd	10.04	-27.79	nd	nd	nd	Seitzmann et al. (201
370	Hygrocybe	sp.(HM020686) aff re	USA	Harvard Forest, MA	42.5129,-72.2138	nd	10.44	-27.85	nd	nd	nd	Seitzmann et al. (201
371	Hygrocybe	sp. (HM020687) aff r	USA	Harvard Forest, MA	42.5129,-72.2138	nd	7.38	-29.52	nd	nd	nd	Seitzmann et al. (201
372	Hygrocybe	sp.(HM020688) aff re	USA	Harvard Forest, MA	42.5129,-72.2138	nd	6.29	-28.96	nd	nd	nd	Seitzmann et al. (201
						Mean	11.98	-27.72	#DIV/0!	#DIV/0!	#DIV/0!	
						SD	4.08	0.86	#DIV/0!	#DIV/0!	#DIV/0!	
						COUNT	20	20	0	0	0	
						Min	5.05	-29.52	0.00	0.00	0.00	
						Max	17.05	-25.62	0.00	0.00	0.00	
	**NON-EUROPEAN D (GABON)**											
373	Hygrocybe	sp. (TU112116)	Gabon	Mbe National Park, Tchimbele	0.6235,10.3993	nd	8.34	-29.97	4.83	38.90	8.05	Tedersoo et al. (2012)
374	Hygrocybe	sp. (TU112120)	Gabon	Mbe National Park, Tchimbele	0.6235,10.3993	nd	11.00	-31.55	6.22	38.43	6.18	Tedersoo et al. (2012)
375	Hygrocybe	sp. (TU112140)	Gabon	Mbe National Park, Tchimbele	0.6235,10.3993	nd	21.05	-29.69	4.39	39.43	8.99	Tedersoo et al. (2012)
376	Hygrocybe	sp. (TU112152)	Gabon	Mbe National Park, Tchimbele	0.6235,10.3993	nd	21.24	-28.91	6.12	43.84	7.17	Tedersoo et al. (2012)
377	Hygrocybe	sp. (TU112156)	Gabon	Mbe National Park, Tchimbele	0.6235,10.3993	nd	18.01	-29.41	3.53	38.72	10.97	Tedersoo et al. (2012)
378	Hygrocybe	sp. "toe-head"	Guyana	Potaro River Basin, Pakaraima	5.3710,-59.1335	nd	7.99	-28.70	nd	nd	nd	Mayor et al., 2009
						Mean	14.60	-29.71	5.02	39.86	8.27	
						SD	6.22	1.02	1.15	2.25	1.83	
						COUNT	6	6	5	5	5	
						Min	7.99	-31.55	3.53	38.43	6.18	
						Max	21.24	-28.70	6.22	43.84	10.97	
	**HYGROPHORUS SPP.**											
379	Hygrophorus	agathosmus	Sweden	Stadsskogen, Uppsala	59.8394,17.6216	nd	6.40	-28.10	nd	nd	nd	Taylor et al. (2003)
380	Hygrophorus	bakerensis	USA	Deer Park, Seattle WA	47.9833,-123.3167	nd	3.50	-25.50	nd	nd	nd	Trudell et al. (2004)
381	Hygrophorus	camarophyllus	Sweden	Stadsskogen, Uppsala	59.8394,17.6216	nd	3.10	-24.60	nd	nd	nd	Taylor et al. (2003)
382	Hygrophorus	camarophyllus	USA	Deer Park, Seattle WA	47.9833,-123.3167	nd	4.10	-25.50	nd	nd	nd	Trudell et al. (2004)
383	Hygrophorus	camarophyllus	USA	Snowbowl, Flagstaff AZ	35.3210, -111.7198	nd	-0.40	-23.60	3.90	40.60	10.41	Hart et al. (2005)
384	Hygrophorus	chrysodon	USA	Deer Park, Seattle WA	47.9833,-123.3167	nd	1.10	-25.70	nd	nd	nd	Trudell et al. (2004)
385	Hygrophorus	eburneus	USA	Deer Park, Seattle WA	47.9833,-123.3167	nd	3.10	-25.70	nd	nd	nd	Trudell et al. (2004)
386	Hygrophorus	flavodiscus	USA	Harvard Forest, MA	42.5129,-72.2138	nd	5.59	-24.95	nd	nd	nd	Trudell et al. (2004)
387	Hygrophorus	flavodiscus	USA	Harvard Forest, MA	42.5129,-72.2138	nd	8.12	-23.25	nd	nd	nd	Trudell et al. (2004)
388	Hygrophorus	fuligineus	USA	Harvard Forest, MA	42.5129,-72.2138	nd	9.00	-26.52	nd	nd	nd	Trudell et al. (2004)
389	Hygrophorus	hypothejus	Sweden	Betsele, Umea	64.6332,18.5506	nd	9.80	nd	3.90	nd	nd	Taylor et al. (2003)
390	Hygrophorus	hypothejus	Scotland	Stirling	56.1514,-3.9145	22-Nov-73	4.37	-24.57	2.11	42.50	20.14	THIS STUDY
391	Hygrophorus	hypothejus	Wales	Coed Gorllwyn	52.9648,-4.1052	12-Nov-06	4.77	-28.29	4.48	41.77	9.33	THIS STUDY
392	Hygrophorus	involutus	New Zealand	Kaimai-Mamaku Forest	-37.5407,175.8706	May-08	9.93	-29.12	2.19	38.22	17.42	THIS STUDY
393	Hygrophorus	lindtneri	France	Breuil-Chenue, Nièvre	47.3028,4.0789	nd	-6.60	-28.60	nd	nd	nd	Zeller et al. (2007)
394	Hygrophorus	olivaceoalbus	Sweden	Aheden, Umea	63.6828,20.0565	nd	-0.80	nd	1.50	nd	nd	Taylor et al. (2003)
395	Hygrophorus	olivaceoalbus	Sweden	Flakalinden, Umea	64.1134, 19.4737	nd	0.10	nd	3.29	nd	nd	Taylor et al. (2003)
396	Hygrophorus	olivaceoalbus	Sweden	Stadsskogen, Uppsala	59.8394,17.6216	nd	-1.70	-26.00	nd	nd	nd	Taylor et al. (2003)
397	Hygrophorus	olivaceoalbus	USA	Hoh, Seattle WA	47.8333,-124.0333	nd	2.70	-24.90	nd	nd	nd	Trudell et al. (2004)
398	Hygrophorus	piceae	Sweden	Aheden, Umea	63.6828,20.0565	nd	3.10	nd	4.40	nd	nd	Taylor et al. (2003)
399	Hygrophorus	purpurascens	USA	Deer Park, Seattle WA	47.9833,-123.3167	nd	7.60	-24.90	nd	nd	nd	Trudell et al. (2004)
						Mean	3.66	-25.87	3.22	40.77	14.32	
						SD	4.16	1.73	1.14	1.87	5.28	
						COUNT	21	17	8	4	4	
						Min	-6.60	-29.12	1.50	38.22	9.33	
						Max	9.93	-23.25	4.48	42.50	20.14	
	**SAPROTROPH (A) SOURHOPE**											
400	*Calocybe*	*carnea*	Scotland	Sourhope	55.4700, -2.2312	30-Sep-04	3.43	-25.38	7.77	41.38	5.33	
401	*Conocybe*	*subovalis*	Scotland	Sourhope	55.4700, -2.2312	30-Sep-04	3.98	-25.31	7.60	39.29	5.17	
402	*Cystoderma*	*amianthinum*	Scotland	Sourhope	55.4700, -2.2312	18-Oct-01	0.10	-25.02	6.25	44.31	*7.09*	
403	*Galerina*	aff. *clavata*	Scotland	Sourhope	55.4700, -2.2312	26-Sep-02	-0.74	P-26.42	5.92	42.34	7.15	13C-enriched
404	*Galerina*	aff. *vittiformis*	Scotland	Sourhope	55.4700, -2.2312	10-Oct-03	-0.53	-25.68	6.89	39.82	5.78	
405	*Galerina*	aff. *vittiformis*	Scotland	Sourhope	55.4700, -2.2312	10-Oct-03	-1.91	-24.46	5.73	41.52	7.25	
406	*Galerina*	aff. *vittiformis*	Scotland	Sourhope	55.4700, -2.2312	10-Oct-03	2.09	-29.43	5.49	41.66	7.59	
407	*Hygrophoropsis*	aurantiaca	Scotland	Sourhope	55.4700, -2.2312	10-Oct-03	0.33	-24.08	6.86	41.84	6.10	
408	*Hygrophoropsis*	aurantiaca	Scotland	Sourhope	55.4700, -2.2312	10-Oct-03	2.41	-22.76	7.33	43.99	6.00	
409	*Hygrophoropsis*	*aurantiaca*	Scotland	Sourhope	55.4700, -2.2312	10-Oct-03	-1.48	-24.96	7.02	43.19	6.15	
410	*Mycena*	*aetites*	Scotland	Sourhope	55.4700, -2.2312	18-Oct-01	0.78	-24.65	5.23	48.94	*9.36*	

411	*Mycena*	*flavoalba*	Scotland	Sourhope	55.4700, -2.2312	18-Oct-01	2.25	-25.01	6.79	44.10	*6.49*
412	*Agaricus*	*langei*	Scotland	Sourhope		5-Nov-03	12.50	-23.55	9.88	40.54	4.10
413	*Panaeolus*	*acuminatus*	Scotland	Sourhope	55.4700, -2.2312	18-Oct-01	2.87	-25.99	7.21	42.77	*5.93*
414	*Psilocybe*	*semilanceata*	Scotland	Sourhope	55.4700, -2.2312	18-Oct-01	0.98	-24.80	3.76	31.00	*8.24*
415	*Stropharia*	*aeruginosa*	Scotland	Sourhope	55.4700, -2.2312	18-Oct-01	0.49	-25.48	5.36	43.08	*8.04*
416	*Stropharia*	*semiglobata*	Scotland	Sourhope	55.4700, -2.2312	18-Oct-01	3.54	-25.31	5.03	42.01	*8.35*
						Mean	1.83	-25.12	6.48	41.87	6.71
						SD	3.27	1.41	1.38	3.54	1.35
						COUNT	17	16	17	17	17
						Min	-1.91	-29.43	3.76	31.00	4.10
						Max	12.50	-22.76	9.88	48.94	9.36
	**SAPROTROPH (B) OTHER EUROPE SITES**										
417	*Cystoderma*	*amianthinum*	Wales	Bronydd Mawr	51.9840,-3.6318	31-Oct-02	0.79	-25.96	6.70	42.83	*6.39*
418	*Lepiota*	*cristata*	England	St. Mary's Church, Trumpingtor	52.1734,0.1085	17-Oct-04	6.34	-21.41	8.61	38.84	4.51
419	*Lepiota*	*cristata*	Wales	Dinas Dinlle	53.1121,-4.34482	11-Sep-03	2.98	-23.96	7.12	44.01	6.18
420	*Lepista*	*nuda*	Wales	Mynachdy'r Graig	52.3471,-4.1191	15-Nov-06	5.17	-21.13	6.20	37.90	6.11
421	*Lepista*	*nuda*	Wales	Aberystwyth University	52.4186,-4.0702	30-Oct-06	2.21	-22.47	8.11	41.03	5.06
422	*Lepista*	*saeva*	Wales	Dinas Dinlle	53.1121,-4.34482	10-Nov-02	-0.05	-22.91	10.12	33.20	*3.28*
423	*Macrolepiota*	*procera*	Wales	**Y Ffor**	52.9191,-4.3885	23-Oct-04	14.65	-24.73	9.09	40.50	4.46
424	*Macrolepiota*	*procera*	Ireland	Burren	53.0337,-9.0547	6-Sep-00	2.04	-24.71	7.39	48.69	6.59
425	*Macrolepiota*	*rhacodes*	England	Clitheroe	53.8067,-2.4294	2002?	7.23	-23.37	7.46	42.50	*5.70*
426	*Mycena*	*leptocephala*	England	Parkgrass	51.8040,-0.3722	11-Nov-03	2.81	-25.76	6.73	42.35	6.29
427	*Mycena*	*leptocephala*	England	Parkgrass	51.8040,-0.3722	11-Nov-03	3.17	-25.26	6.51	41.62	6.39
428	*Mycena*	*leptocephala*	England	Parkgrass	51.8040,-0.3722	11-Nov-03	4.66	-26.28	6.10	41.05	6.73
429	*Mycena*	*leptocephala*	England	Parkgrass	51.8040,-0.3722	11-Nov-03	-0.05	-24.59	4.51	42.54	9.43
430	*Mycena*	*pura*	Wales	Dinas Dinlle	53.1121,-4.34482	10-Nov-02	3.35	-23.82	7.39	42.44	*5.74*
431	*Panaeolus*	*acuminatus*	Wales	Bronydd Mawr	51.9840,-3.6318	31-Oct-02	2.21	-26.38	6.92	42.01	*6.07*
432	*Psilocybe*	*coprophila*	Wales	Pont y Gromlech	53.0889,-4.0468	27-Oct-04	0.05	-25.72	6.26	39.68	6.34
433	*Psilocybe*	*semilanceata*	England	Clitheroe	53.8067,-2.4294	6-Nov-02	0.31	-26.26	5.23	43.85	*8.38*
434	*Psilocybe*	*semilanceata*	Wales	Bronydd Mawr	51.9840,-3.6318	31-Oct-02	1.09	-26.40	5.96	40.24	*6.75*
435	*Stropharia*	*aeruginosa*	Wales	Bronydd Mawr	51.9840,-3.6318	31-Oct-02	-0.26	-25.65	5.60	42.76	*7.64*
436	*Stropharia*	*semiglobata*	Wales	Bronydd Mawr	51.9840,-3.6318	31-Oct-02	5.05	-26.52	5.25	42.01	*8.00*
437	Lepista	nuda	Germany	Amorbach	49.6473,9.2013	5/11/15	4.74	-22.21	4.92	40.79	5.92
438	Lepista	nuda	Germany	Amorbach	49.6473,9.2013	5/11/15	3.70	-21.33	5.30	40.19	5.42
439	Lepista	nuda	Germany	Amorbach	49.6473,9.2013	5/11/15	1.56	-21.86	4.79	41.47	6.18
440	Lepista	nuda	Germany	Amorbach	49.6473,9.2013	5/11/15	6.01	-22.50	4.48	40.42	6.44
441	Lepista	nuda	Germany	Amorbach	49.6473,9.2013	5/11/15	4.01	-21.82	4.71	40.98	6.22
442	Lepista	nuda	Germany	Amorbach	49.6473,9.2013	5/11/15	3.51	-21.73	4.86	41.45	6.09
443	Lepista	nuda	Germany	Amorbach	49.6473,9.2013	5/11/15	4.54	-22.27	5.00	40.94	5.85
444	Lepista	nuda	Germany	Amorbach	49.6473,9.2013	5/11/15	4.95	-22.61	4.45	40.35	6.47
445	Lepista	nuda	Germany	Amorbach	49.6473,9.2013	5/11/15	3.71	-22.12	4.74	39.83	6.01
446	Lepista	nuda	Germany	Amorbach	49.6473,9.2013	5/11/15	1.23	-22.57	4.52	38.12	6.02
447	Lepista	nuda	Germany	Amorbach	49.6473,9.2013	5/11/15	5.58	-21.68	4.93	40.05	5.81
448	Lepista	nuda	Germany	Amorbach	49.6473,9.2013	5/11/15	4.94	-21.81	5.14	40.28	5.59
449	Lepista	nuda (ring)	Germany	Amorbach	49.6473,9.2013	5/11/15	3.89	-21.46	5.72	41.18	5.14
450	Lepista	nuda (ring)	Germany	Amorbach	49.6473,9.2013	5/11/15	2.77	-21.70	5.04	39.81	5.64
451	Lepista	nuda (ring)	Germany	Amorbach	49.6473,9.2013	5/11/15	4.68	-22.02	5.27	39.67	5.38
452	Lepista	nuda (ring)	Germany	Amorbach	49.6473,9.2013	5/11/15	3.05	-21.89	5.50	39.25	5.10
453	Lepista	nuda (ring)	Germany	Amorbach	49.6473,9.2013	5/11/15	2.92	-21.84	4.34	33.93	5.58
454	Lepista	nuda (ring)	Germany	Amorbach	49.6473,9.2013	5/11/15	4.54	-21.81	5.13	41.17	5.73
455	Lepista	nuda (ring)	Germany	Amorbach	49.6473,9.2013	5/11/15	4.38	-21.82	4.82	39.29	5.82
456	Lepista	nuda (ring)	Germany	Amorbach	49.6473,9.2013	5/11/15	5.03	-21.79	4.91	39.53	5.75
457	Lepista	nuda (ring)	Germany	Amorbach	49.6473,9.2013	5/11/15	4.32	-21.42	4.86	39.02	5.74
458	Lepista	nuda (ring)	Germany	Amorbach	49.6473,9.2013	5/11/15	4.00	-21.37	5.37	40.73	5.42
459	Pseudoclitocybe	cyathiformis	Germany	Bischbrunn, Spessart	49.8873,9.4707	6/11/15	-0.36	-28.10	4.85	42.55	6.27
460	Pseudoclitocybe	cyathiformis	Germany	Bischbrunn, Spessart	49.8873,9.4707	6/11/15	-0.36	-28.52	4.24	41.74	7.04
461	Pseudoclitocybe	cyathiformis	Germany	Bischbrunn, Spessart	49.8873,9.4707	6/11/15	0.39	-27.97	4.93	41.62	6.03
462	Pseudoclitocybe	cyathiformis	Germany	Bischbrunn, Spessart	49.8873,9.4707	6/11/15	-0.05	-28.02	4.57	41.15	6.43
463	Pseudoclitocybe	cyathiformis	Germany	Bischbrunn, Spessart	49.8873,9.4707	6/11/15	-0.04	-27.83	4.68	38.67	5.90
464	Pseudoclitocybe	cyathiformis	Germany	Bischbrunn, Spessart	49.8873,9.4707	6/11/15	-0.10	-27.75	4.19	39.94	6.80
465	Pseudoclitocybe	cyathiformis	Germany	Bischbrunn, Spessart	49.8873,9.4707	6/11/15	-0.01	-28.02	4.28	40.88	6.82
466	Pseudoclitocybe	cyathiformis	Germany	Bischbrunn, Spessart	49.8873,9.4707	6/11/15	-0.66	-27.59	5.68	41.44	5.21
						Mean	3.01	-23.97	5.67	40.73	6.08
						SD	2.72	2.43	1.34	2.29	0.97
						COUNT	50	50	50	50	50
						Min	-0.66	-28.52	4.19	33.20	3.28
						Max	14.65	-21.13	10.12	48.69	9.43
	**ENTOMOPATHOGEN**										
467	*Cordyceps*	*militaris*		Sourhope	55.4700, -2.2312	15-Nov-03	10.25	-30.93	6.98	43.94	6.30
468	*Cordyceps*	*militaris*		Sourhope	55.4700, -2.2312	16-Oct-03	5.46	-30.47	7.32	43.09	5.89
469	*Cordyceps*	*militaris*		Sourhope	55.4700, -2.2312	24-Sep-03	8.30	-29.31	7.62	44.23	5.80
						Mean	8.00	-30.24	7.31	43.75	6.00
						SD	2.41	0.83	0.32	0.59	0.26

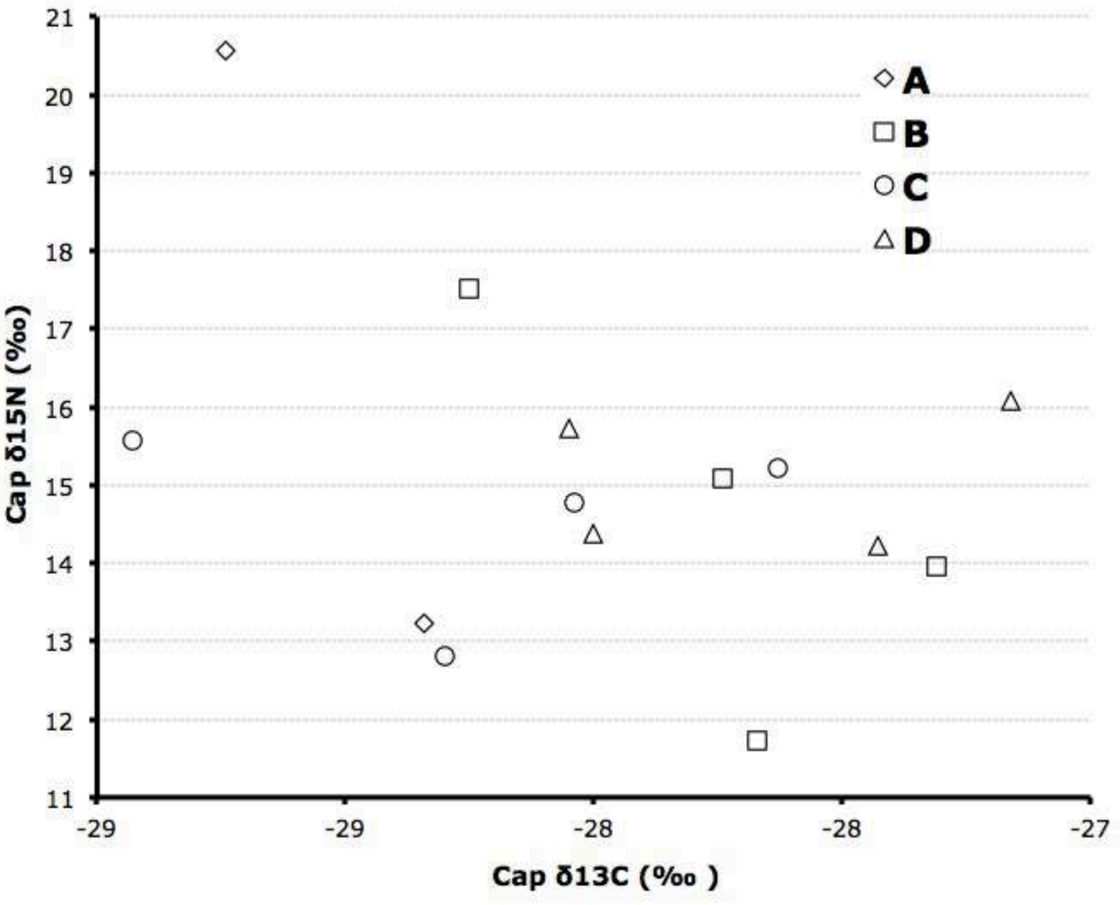

Suppdata 4a. $\delta^{15}N$ and $\delta^{13}C$ values for cap tissues of basidiocarps from different age classes, ranging from A (button) to D (fully mature)

Suppdata 4b. $\delta^{15}N$ and $\delta^{13}C$ values for cap and stipe tissues

Species	Site	Collection Date	Cap δ15N (‰)	Stipe δ15N (‰)	δ15N cap-stipe (‰)	Cap δ13C (‰)	Stipe δ13C (‰)	δ13C cap - stipe (‰)	Cap total N (%)	Cap total C (%)	Stipe total N (%)	Stipe % total C	Cap C/N ratio	Stipe C/N ratio
Hygrocybe conica	Sourhope	02/10/01	11.57	10.64	**0.93**	-29.28	-29.16	**-0.12**	5.45	49.19	4.8	42.93	9.03	8.94
Hygrocybe conica	Sourhope	02/10/01	12.49	11.19	**1.30**	-30.09	-30.36	**0.27**	5.41	47.66	3.72	41.54	8.81	11.17
Hygrocybe conica	Sourhope	02/10/01	14.23	12.8	**1.43**	-28.85	-28.99	**0.14**	5.96	45.86	4.23	41.16	7.69	9.73
Gliophorus laeta	Sourhope	02/10/01	14.12	11.32	**2.80**	-28.96	-29.3	**0.34**	4.95	48.61	2.3	38.2	9.82	16.61
Gliophorus laeta	Sourhope	02/10/01	14.77	11.86	**2.91**	-28.56	-28.82	**0.26**	5.85	48.52	2.72	39.37	8.29	14.47
Gliophorus laeta	Sourhope	02/10/01	16.78	12.63	**4.15**	-27.59	-27.82	**0.23**	5.49	42.02	2.46	37.53	7.65	15.26
Cuphophyllus pratensis	Sourhope	02/10/01	14.44	10.11	**4.33**	-28.36	-28.56	**0.20**	7.08	45.48	3.64	41.11	6.42	11.29
Cuphophyllus pratensis	Sourhope	02/10/01	16.58	11.04	**5.54**	-29.37	-29.23	**-0.14**	7.59	44.73	2.93	41.37	5.89	14.12
Cuphophyllus pratensis	Sourhope	02/10/01	18.08	12.31	**5.77**	-29.37	-29.1	**-0.27**	6.40	45.60	3.06	42.49	7.13	13.89
Hygrocybe splendidissima	Sourhope	02/10/01	12.09	11.37	**0.72**	-28.42	-29.06	**0.64**	6.05	48.66	3.56	41.53	8.04	11.67
Cuphophyllus virgineus	Sourhope	18/10/01	16.32	12.12	**4.20**	-27.66	-28.03	**0.37**	5.44	45.77	2.19	40.44	8.41	18.47
Hygrocybe punicea	Parkgrass	05/11/08	13.93	13.50	**0.42**	-29.59	-30.54	**0.95**	8.86	48.09	3.62	40.20	5.43	11.10
Hygrocybe punicea	Parkgrass	05/11/08	14.01	11.46	**2.55**	-29.84	-31.62	**1.78**	9.72	43.62	4.58	43.53	4.49	9.49
Hygrocybe punicea	Parkgrass	05/11/08	14.83	12.35	**2.47**	-30.59	-30.72	**0.13**	8.35	44.69	3.53	38.31	5.35	10.86
Hygrocybe punicea	Parkgrass	05/11/08	16.90	15.54	**1.36**	-29.73	-30.26	**0.53**	8.72	44.91	3.82	41.36	5.15	10.81
		**Mean**	14.74	12.02	2.73	-29.08	-29.44	0.35	6.75	46.23	3.41	40.74	7.17	12.53
		**SD**	1.89	1.32	1.74	0.86	1.05	0.50	1.53	2.13	0.79	1.75	1.63	2.79
		**Min**	11.57	10.11	0.42	-30.59	-31.62	-0.27	4.95	42.02	2.19	37.53	4.49	8.94
		**Max**	18.08	15.54	5.77	-27.59	-27.82	1.78	9.72	49.19	4.80	43.53	9.82	18.47

Suppdata 5. Basidiocarps of *Cuphophyllus lacmus* (A/B/C; Lundy Island, Devon, England) growing in a pure stand of *Calluna vulgaris* and *Hygrocybe cantharellus* (D; Cwm Dulyn, Gwynedd, Wales) growing amongst bog vegetation mostly comprised of *Sphagnum*. These basidiocarps were unusual in having atypically low $\delta^{15}N$ signatures (ca. +9.5‰ for *C. lacmus* and ca. +1‰ for *H. cantharellus*; see Suppdata 2)

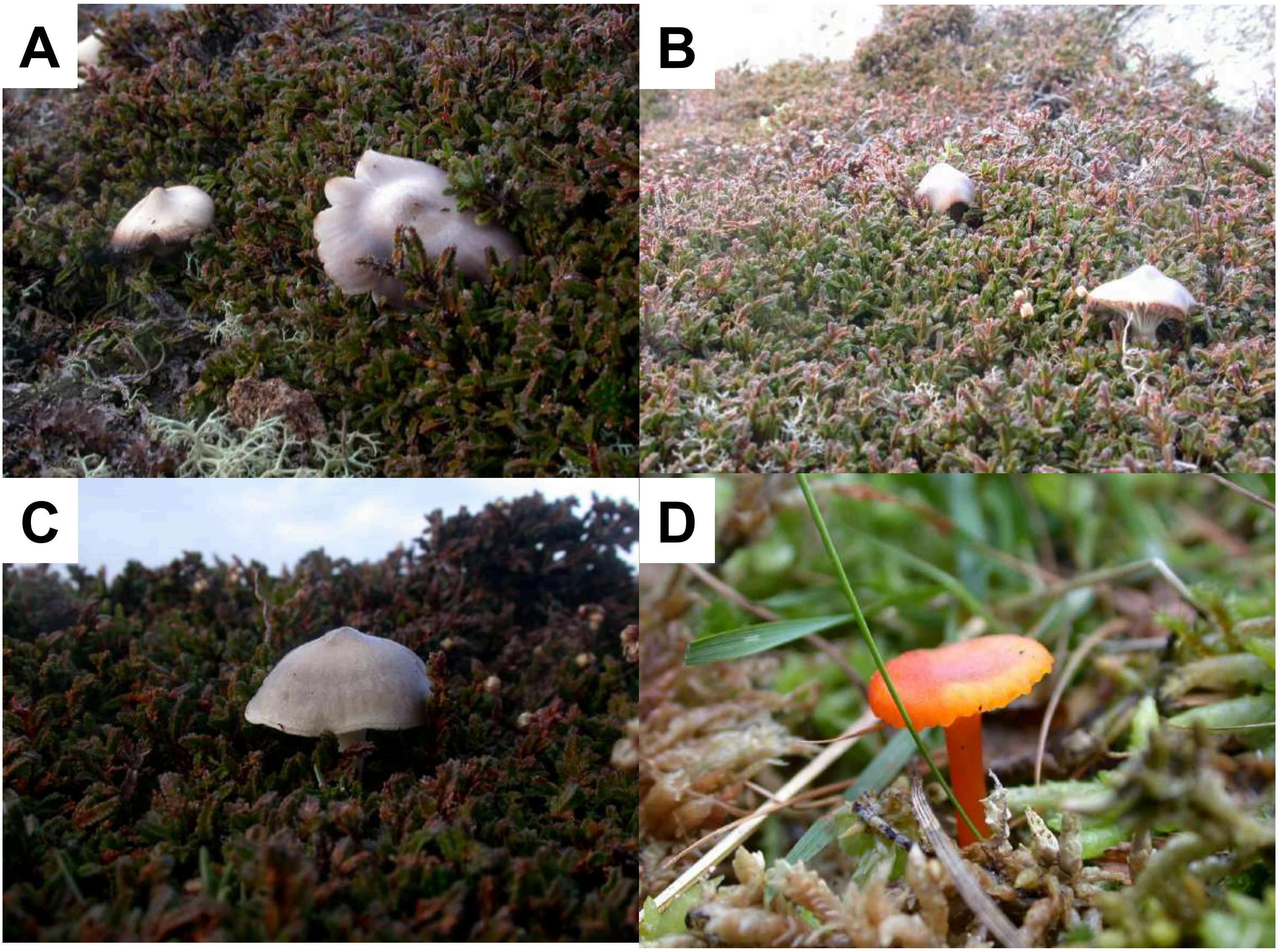

Suppdata 6. Distribution of *C. pratensis* FBs and δ^{15}N values

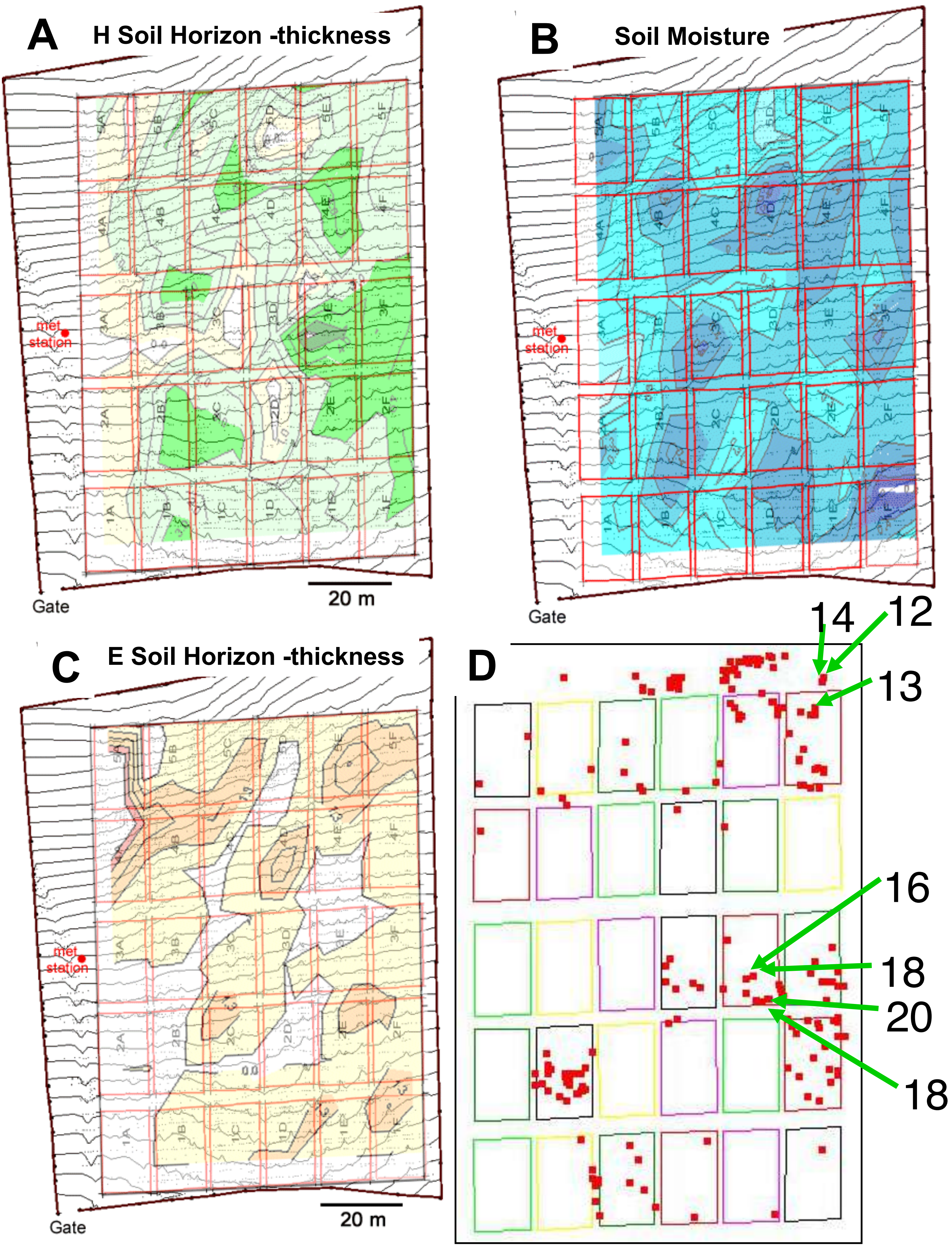

A, B, C- Soil data from http://soilbio.nerc.ac.uk/Extranet/data_smaps.htm#Soil Moisture Content

D. Location of *Cuphophyllus pratensis* basidiocarps at Sourhope (squares) indicating δ^{15}N for selected samples.

Suppdata 7. $^{13}CO_2$ pulse labelling of vegetation at Sourhope and examination of basidiocarps subsequently formed near to pulse sites.

Method: In order to examine the potential flow of fresh photosynthate to waxcap basidiocarps, pulses of ^{13}C-enriched CO_2 (50 atom%) at 370 ppm were supplied using a mobile Stable Isotope Delivery (SID) system (Ostle et al., 2000). This system comprised an isotope-mixing unit with flow control to a series of clear acrylic labelling chambers (20 cm high and 40 cm diam), which were pushed approximately 10 mm into the surface soil F horizon. A flow rate of 6 $l.min^{-1}.chamber^{-1}$ provided a gas residence time of ca. 5 mins. Seven pulse sites were set up on 12/9/02 each placed on plots undisturbed by earlier SoilBiodiversity sampling etc.. Pulse duration was for 6 hrs. Basidiocarps formed within 150 cm of the pulse site were collected over succeeding weeks.

Results: Following the pulse labelling of several points at the Sourhope fieldsite in 2001 with ^{13}C-enriched CO_2, all basidiocarps within 150 cm of pulse points during subsequent weeks were subjected to isotope analysis after recording their exact position in relation to the pulse domes. Seven basidiocarps (all *C. pratensis*) were observed within 100 cm of the edge of pulse domes and one of these (growing 20 cm from the edge of the dome 14 d after the pulse date) was found to be highly enriched (δ^{13}C +92.8‰; 0.4018 atom%). Other basidiocarps showed no ^{13}C enrichment (all < δ^{13}C -28.3‰) but were all located >60 cm from the edge of the pulse dome.

Species	Plot number	Time since pulse (d)	Distance from edge of pulse dome (cm)	δ13C
Cuphophyllus pratensis	**2F**	**14**	**20**	**92.79**
Cuphophyllus pratensis	2F	43	80	-28.26
Cuphophyllus pratensis	2B	14	70	-29.20
Cuphophyllus pratensis	2B	29	90	-28.70
Cuphophyllus pratensis	2B	29	120	-28.77
Cuphophyllus pratensis	2B	29	110	-28.96
Cuphophyllus pratensis	3E	29	130	-29.18

Ostle N, Ineson P, Benham D, Sleep D. 2000. Carbon assimilation and turnover in grassland vegetation using an in situ $^{13}CO_2$ pulse labelling system. *Rapid Communications in Mass Spectrometry* **14,** 1345-1350.

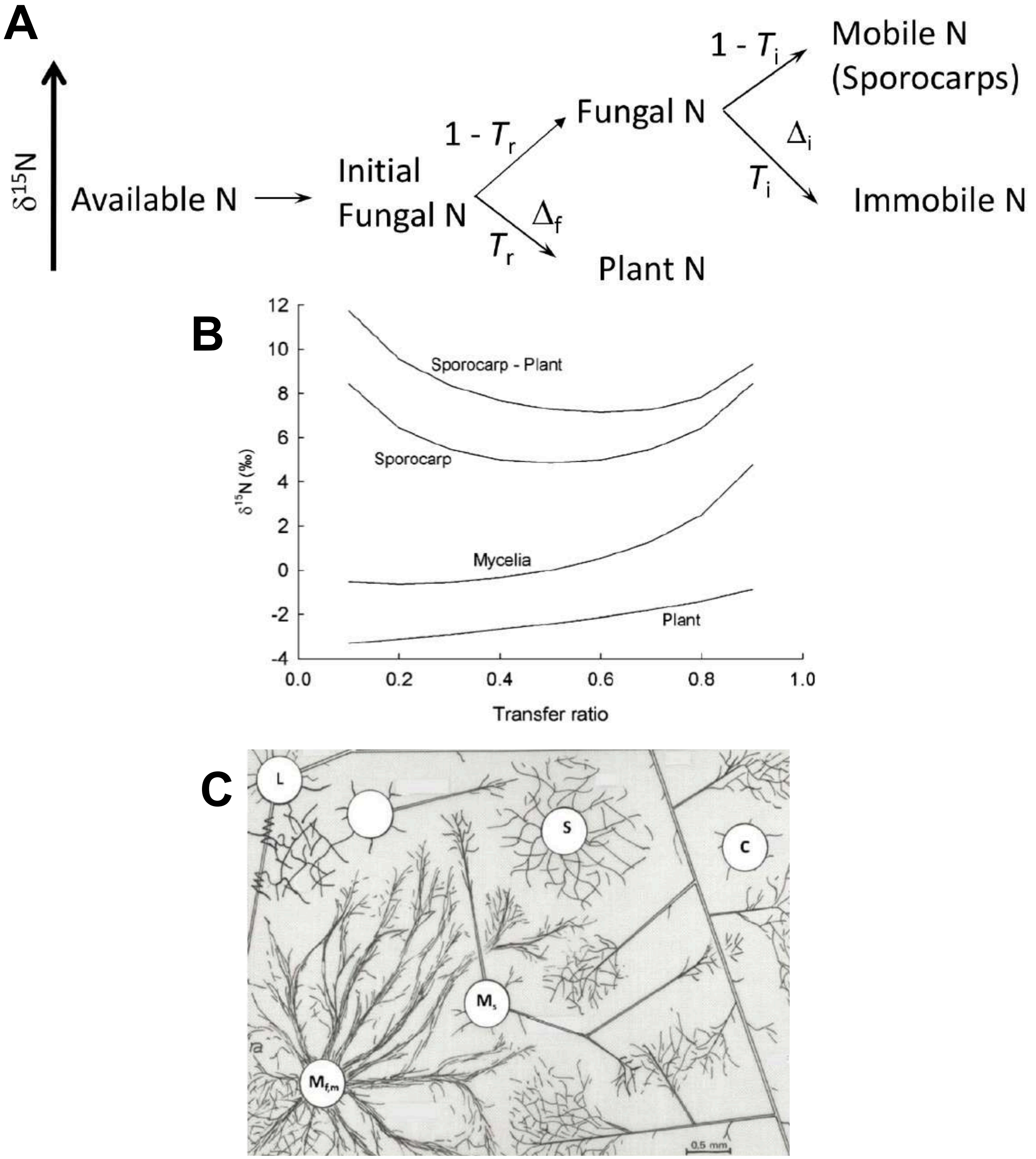

Suppdata 8. The Rayleigh distillation model of Hobbie and Agerer (2009), illustrating (A) how N taken up by fungi is partitioned between the fungus and associated plant and then within the fungal mycelium as mobile N (which contributes to sporocarp formation) and immobile N (which is sequestered into cell wall components, including chitin and cell wall proteins). The extent of ^{15}N enrichment in plant mycelium and sporocarp depends on the relative proportions of total N distributed to these various. sinks (B). Thus species with more extensive or restricted mycelial systems differ in the amount of immobile N sequestered into cell wall material. Thus species with extensive mycelial systems are predicted to exhibit greater levels of ^{15}N enrichment in sporocarps.

Suppdata 9. δ15N values of selected soil invertebrate guilds and taxa

Soil invertebrates	$\delta^{15}N$ ‰ relative to litter	Reference
Saprotrophs (meadow)	-0.6 to +4.8	Tiunov et al. (2007)
Predators (meadow)	+6.5 to +9.3	Tiunov et al. (2007)
Dipteran larvae	-2.9 to +7.7	Seeber et al. (2005)
Endogeic earthworms	+3.3 to +10.6	Neilson et al. (2000)
Enchytraeidae worms	+7.0 to +13.9	Schmidt et al. (2004)

Neilson, R., Boag, B., and Smith, M. (2000) Earthworm δ13C and δ15N analyses suggest that putative functional classifications of earthworms are site-specific and may also indicate habitat diversity. Soil Biol Biochem 32: 1053-1061.

Schmidt, O., Curry, J.P., Dyckmans, J., Rota, E., and Scrimgeour, C.M. (2004) Dual stable isotope analysis (δ13C and δ15N) of soil invertebrates and their food sources. Pedobiologia 48: 171-180.

Seeber, J., Seeber, G.U.H., Kessler, W., Langel, R., Scheu, S., and Meyer, E. (2005) Abundance and trophic structure of macro-decomposers on alpine pastureland (Central Alps, Tyrol): effects of abandonment of pasturing. Pedobiologia 49: 221-228.

Tiunov, A.V. (2007) Stable isotopes of carbon and nitrogen in soil ecological studies. Biol Bull 34: 395-407.

Suppdata 10. Raw data and references for published studies relating to Fig. 3

	Study	Hygrophoraceae				
**This study**	abbrev.	d13C	SE	d15N	SE	n
Cuphophyllus	Cu	-29.3	0.07	15.2	0.13	178
Gliophorus	Gl	-28.4	0.22	15.1	0.66	24
Humidicutis	Hu	-27.1	0.32	15.2	0.44	13
Hygrocybe	Hc	-29.0	0.11	12.5	0.34	127
Hygrophorus	Hg	-25.9	0.42	3.7	0.91	21
Neohygrocybe	Ne	-29.4	0.28	14.4	1.61	3
Porpolomopsis	Po	-28.9	0.34	15.9	0.48	8
Grassland Saprotrophs	GS	-24.3	0.39	2.6	0.34	63

		Ectomycorrhizal					Saprotrophic				
**Published studies**	abbrev.	d13C	SE	d15N	SE	n	d13C	SE	d15N	SE	n
Agerer et al. 2011	Ag	-24.5	0.23	11.8	0.65	33	-22.8	0.34	-1.5	0.78	15
Averill & Finzi 2011	Av	-25.9	0.19	3.5	0.47	39	-21.9	1.32	8.5	0.43	2
Clemmensen et al. 2006	C	-26.9	0.18	4.1	1.38	13	-24.2	0.31	1.7	1.16	9
Hart et al. 2006	Ha	-24.0	0.18	3.8	0.70	21	-22.0	0.26	2.2	0.49	26
Hasselquist & Hogberg, 2014	Hs	-26.2	0.05	5.9	0.25	99					
Henn & Chapela 2001	He	-25.8	0.30	9.0	0.93	18	-22.5	0.25	-0.1	0.43	25
Hobbie et al. 1999	H9	-25.4	0.22	4.5	1.23	4	-22.9	0.28	-1.9	1.22	4
Hobbie et al. 2001	H1	-26.2	0.29	3.9	0.50	20	-22.8	0.32	-1.8	0.43	25
Kohzu et al. 1999	K	-25.0	0.17	5.9	0.57	90	-23.1	0.18	0.1	0.33	90
Hobbie et al. (2005)	H5	-25.7	0.20	-1.6	0.33	54	-21.4	0.29	-2.3	0.20	55
M. Trappe (2014)	Tp	-24.2	0.37	10.6	1.44	11	-20.2	0.65	0.7	1.44	10
Mayor et al 2009	M	-26.0	0.20	5.7	0.45	29	-24.9	0.30	1.6	0.49	20
Seitzman et al. 2011	S	-26.0	0.15	6.7	0.82	44	-26.8	0.74	3.2	1.60	12
Taylor et al. 2003	Ta	-25.7	0.11	6.2	0.29	139	-23.1	0.28	1.1	0.75	17
Trudell et al. 2004	Tr	-25.3	0.07	5.1	0.32	118	-23.1	0.17	-1.9	0.21	61
Wilson et al. 2007	W	-23.2	0.17	8.1	0.72	4	-21.0	0.67	0.5	0.47	4
Zeller et al 2007	Z	-25.4	0.19	3.3	0.52	53	-22.7	0.21	-1.6	0.39	31

Agerer et al. 2011	Agerer, R., Christan, J., Mayr, C., Hobbie, E.A., 2011. Isotopic signatures and trophic status of Ramaria. Mycol. Prog. 11, 1147-1159.
Averill & Finzi 2011	Averill, C., Finzi, A., 2011. Increasing plant use of organic nitrogen with elevation is reflected in nitrogen uptake rates and ecosystem δ15N. Ecology 92, 883-891.
Clemmensen et al. 2006	Clemmensen, K.E., Michelsen, A., Jonasson, S., Shaver, G.R., 2006. Increased ectomycorrhizal fungal abundance after long-term fertilization and warming of two arctic tundra ecosystems. New Phytol. 171, 391-404.
Hart et al. 2006	Hart, S., Gehring, C., Selmants, P., Deckert, R., 2006. Carbon and nitrogen elemental and isotopic patterns in macrofungal sporocarps and trees in semiarid forests of the south-western USA. Funct. Ecol. 20, 42-51.
Hasselquist & Hogberg, 2014	Hasselquist, N.J., Högberg, P., 2014. Dosage and duration effects of nitrogen additions on ectomycorrhizal sporocarp production and functioning: an example from two N-limited boreal forests. Ecology and evolution 4, 3015-
Henn & Chapela 2001	Henn, M.R., Chapela, I.H., 2001. Ecophysiology of C-13 and N-15 isotopic fractionation in forest fungi and the roots of the saprotrophic-mycorrhizal divide. Oecologia 128, 480-487.
Hobbie et al. 1999	Hobbie, E.A., Macko, S.A., Shugart, H.H., 1999. Insights into nitrogen and carbon dynamics of ectomycorrhizal and saprotrophic fungi from isotopic evidence. Oecologia 118, 353-360.
Hobbie et al. 2001	Hobbie, E.A., Weber, N.S., Trappe, J.M., 2001. Mycorrhizal vs. saprotrophic status of fungi: the isotopic evidence. New Phytol. 150, 601-610.
Kohzu et al. 1999	Kohzu, A., Yoshioka, T., Ando, T., Takahashi, M., Koba, K., Wada, E., 1999. Natural 13C and 15N abundance of field-collected fungi and their ecological implications. New Phytol. 144, 323-330.
Hobbie et al. (2005)	Hobbie, E.A., Jumpponen, A., Trappe, J., 2005. Foliar and fungal (15) N :(14) N ratios reflect development of mycorrhizae and nitrogen supply during primary succession: testing analytical models. Oecologia 146, 258-268.
M. Trappe (2014)	Trappe, M.J., Smith, M.E., Hobbie, E.A., 2015. Exploring the phylogenetic affiliations and the trophic mode of Sedecula pulvinata (Sedeculaceae). Mycologia 107, 688-696.
Mayor et al 2009	Mayor, J.R., Schuur, E.A., Henkel, T.W., 2009. Elucidating the nutritional dynamics of fungi using stable isotopes. Ecol. Lett. 12, 171-183.
Seitzman et al. 2011	Seitzman, B.H., Ouimette, A., Mixon, R.L., Hobbie, E.A., Hibbett, D.S., 2011. Conservation of biotrophy in Hygrophoraceae inferred from combined stable isotope and phylogenetic analyses. Mycologia 103, 280-290
Taylor et al. 2003	Taylor, A.F.S., Fransson, P.M., Hogberg, P., Hogberg, M.N., Plamboeck, A.H., 2003. Species level patterns in 13C and 15N abundance of ectomycorrhizal and saprotrophic fungal sporocarps. New Phytol. 159, 757-774.
Trudell et al. 2004	Trudell, S.A., Rygiewicz, P.T., Edmonds, R.L., 2004. Patterns of nitrogen and carbon stable isotope ratios in macrofungi, plants and soils in two old growth conifer forests. New Phytol. 164, 317-335.
Wilson et al. 2007	Wilson, A.W., Hobbie, E.A., Hibbett, D.S., 2007. The ectomycorrhizal status of Calostoma cinnabarinum determined using isotopic, molecular, and morphological methods. Botany 85, 385-393.
Zeller et al 2007	Zeller, B., Brechet, C., Maurice, J.-P., Le Tacon, F., 2007. δ13C and δ15N isotopic fractionation in trees, soils and fungi in a natural forest stand and a Norway spruce plantation. Annals of Forest Science 64, 419-429.
